# Effect of high-fat diet and morning or evening exercise on lipoprotein subfraction profiles: secondary analysis of a randomised trial

**DOI:** 10.1038/s41598-023-31082-0

**Published:** 2023-03-10

**Authors:** Trine Moholdt, Evelyn B. Parr, Brooke L. Devlin, Guro F. Giskeødegård, John A. Hawley

**Affiliations:** 1 Department of Circulation and Medical Imaging, Norwegian University of Science and Technology, Trondheim, Norway; 2 Women’s Clinic, St. Olavs Hospital, Trondheim, Norway; 3 Exercise and Nutrition Research Program, Mary MacKillop Institute for Health Research, Australian Catholic University, Fitzroy, VIC 3065 Australia; 4 School of Human Movement and Nutrition Sciences, University of Queensland, Brisbane, QLD Australia; 5 K.G. Jebsen Center for Genetic Epidemiology, Department of Public Health and Nursing, Norwegian University of Science and Technology, Trondheim, Norway

**Keywords:** Dyslipidaemias, Obesity

## Abstract

We investigated the effect of a high-fat diet (HFD) on serum lipid subfractions in men with overweight/obesity and determined whether morning or evening exercise affected these lipid profiles. In a three-armed randomised trial, 24 men consumed an HFD for 11 days. One group of participants did not exercise (*n* = 8, CONTROL), one group trained at 06:30 h (*n* = 8, EXam), and one group at 18:30 h (*n* = 8, EXpm) on days 6–10. We assessed the effects of HFD and exercise training on circulating lipoprotein subclass profiles using NMR spectroscopy. Five days of HFD induced substantial perturbations in fasting lipid subfraction profiles, with changes in 31/100 subfraction variables (adjusted *p* values [*q*] < 0.05). Exercise training induced a systematic change in lipid subfraction profiles, with little overall difference between EXam and EXpm. Compared with CONTROL, exercise training reduced serum concentrations of > 20% of fasting lipid subfractions. EXpm reduced fasting cholesterol concentrations in three LDL subfractions by ⁓30%, while EXam only reduced concentration in the largest LDL particles by 19% (all *q* < 0.05). Lipid subfraction profiles changed markedly after 5 days HFD in men with overweight/obesity. Both morning and evening exercise training impacted subfraction profiles compared with no exercise.

## Introduction

High levels of circulating low-density lipoprotein (LDL) cholesterol is a major risk factor predisposing to atherosclerotic cardiovascular disease (ASCVD), and the primary target for lipid-lowering therapies^1,2^. Furthermore, the inverse relationship between plasma high-density lipoprotein (HDL) cholesterol and ASCVD risk is among the most robust and reproducible associations in observational epidemiology^3^. Thus, HDL cholesterol is included as a critical component in ASCVD risk prediction guidelines from both the European Society of Cardiology and the American Heart Association^4,5^. However, lipid fractions in the blood vary in particle size, density, and in concentrations and composition of lipoproteins. Conventional measures of circulating lipids cannot differentiate between the various subfractions, many of which may have contrasting relationships with risk of ASCVD. For example, small dense LDL particles are associated with incident ASCVD, independent of traditional risk factors including total LDL cholesterol concentrations^6^. Moreover, only the largest subclasses of HDL, and not small HDL, were inversely associated with risk of myocardial infarction in the China Kadoorie Biobank^7^.

Lifestyle modification is the cornerstone of ASCVD prevention. Guidelines for lipid modification to reduce cardiovascular risk advocate diets low in saturated fat with a focus on wholegrain products, vegetables, fruit, and fish^4^. However, the results of several systematic reviews and meta-analyses^8,9^, as well as one of the most extensive studies in recent years on the association of fats and carbohydrate intake with ASCVD and mortality (PURE)^10^, do not support the guidelines that advocate low consumption of total saturated fats. Furthermore, a recent systematic review and meta-analysis reported that dietary interventions which restricted carbohydrate intake (and were high in dietary fat) decreased the numbers of total and small LDL particles^11^.

A physically active lifestyle is associated with substantially reduced ASCVD mortality^12^. Aerobic exercise training can improve lipid profiles, inducing reductions in overall concentrations of LDL and triglycerides, and increased HDL concentrations^13^, but current evidence is equivocal^14^. We reported substantial alterations in lipid-related serum metabolites and elevations in LDL cholesterol after a short-term high-fat diet (HFD) intervention in men with overweight/obesity, and showed that some of these alterations were reversed after daily exercise training performed in the evening for just 5 days^15^. In this secondary analysis of a randomised trial, we determined the lipoprotein subclass profile using nuclear magnetic resonance (NMR) spectroscopy after 5 days of HFD, and assessed whether exercise training undertaken in the morning or in the evening would modulate the effects of the HFD on lipoprotein subclass profiles.

## Results

### Participants

**Figure 1:**
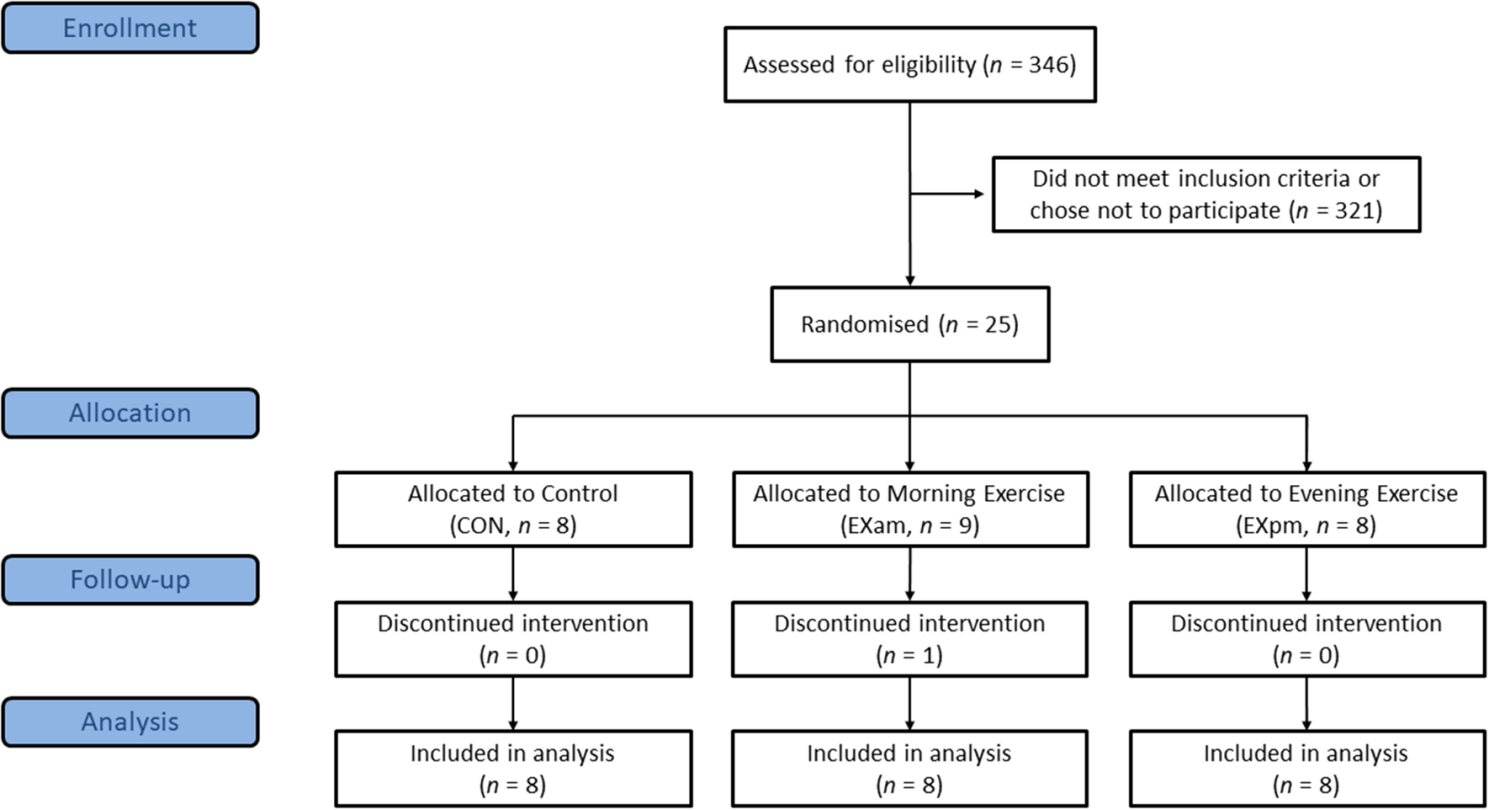
Flowchart of participants.

Twenty-four of the 25 participants completed the full protocol (Fig. 1). Participants were aged 36 ± 4 years and had a body mass index (BMI) 31.2 ± 2.3 kg/m^2^ at baseline. Table 1 shows baseline characteristics of participants, according to group. Data collection commenced in March 2017 and was completed in August 2017, with the lipid NMR analyses undertaken in February 2021. Primary findings from the trial are published elsewhere^15^. There were no unintended or adverse effects of the intervention.

**Table 1:** Baseline characteristics of participants included in analyses, according to group. Lipids measured by clinical chemistry. Data are mean ± SD.

	Morning exercise (*n* = 8)	Evening exercise (*n* = 8)	Control group (*n* = 8)
Age, years	35 ± 4	36 ± 5	36 ± 4
Body mass, kg	103.1 ± 16.1	99.3 ± 8.1	94.5 ± 10.4
BMI, kg/m^2^	31.9 ± 1.9	31.0 ± 2.8	30.7 ± 2.3
Fat mass, kg	36.1 ± 7.0	32.9 ± 5.3	31.3 ± 3.0
Fat mass, %	36.0 ± 3.1	34.1 ± 3.4	33.6 ± 3.6
Fat-free mass, kg	67.2 ± 10.1	66.7 ± 3.9	65.4 ± 9.0
Visceral fat mass, g	2013 ± 1424	1480 ± 573	1969 ± 495
Systolic blood pressure, mm Hg	126 ± 10	131 ± 9	129 ± 8.6
Diastolic blood pressure, mm Hg	81 ± 5	84 ± 7	85 ± 8.6
Peak oxygen uptake, mL·kg^−1^·min^−1^	28.5 ± 6.1	31.2 ± 4.4	29.0 ± 4.3
Cholesterol (mmol/L)	5.0 ± 0.8	4.3 ± 0.6	4.9 ± 0.8
HDL-cholesterol (mmol/L)	1.01 ± 0.2	1.10 ± 0.2	0.99 ± 0.2
LDL-cholesterol (mmol/L)	3.3 ± 0.6	2.7 ± 0.5	3.1 ± 0.8
Triglycerides (mmol/L)	1.63 ± 0.7	1.23 ± 0.6	1.78 ± 0.8

### Changes in lipoprotein profiles after 5 days of high-fat diet

**Figure 2:**
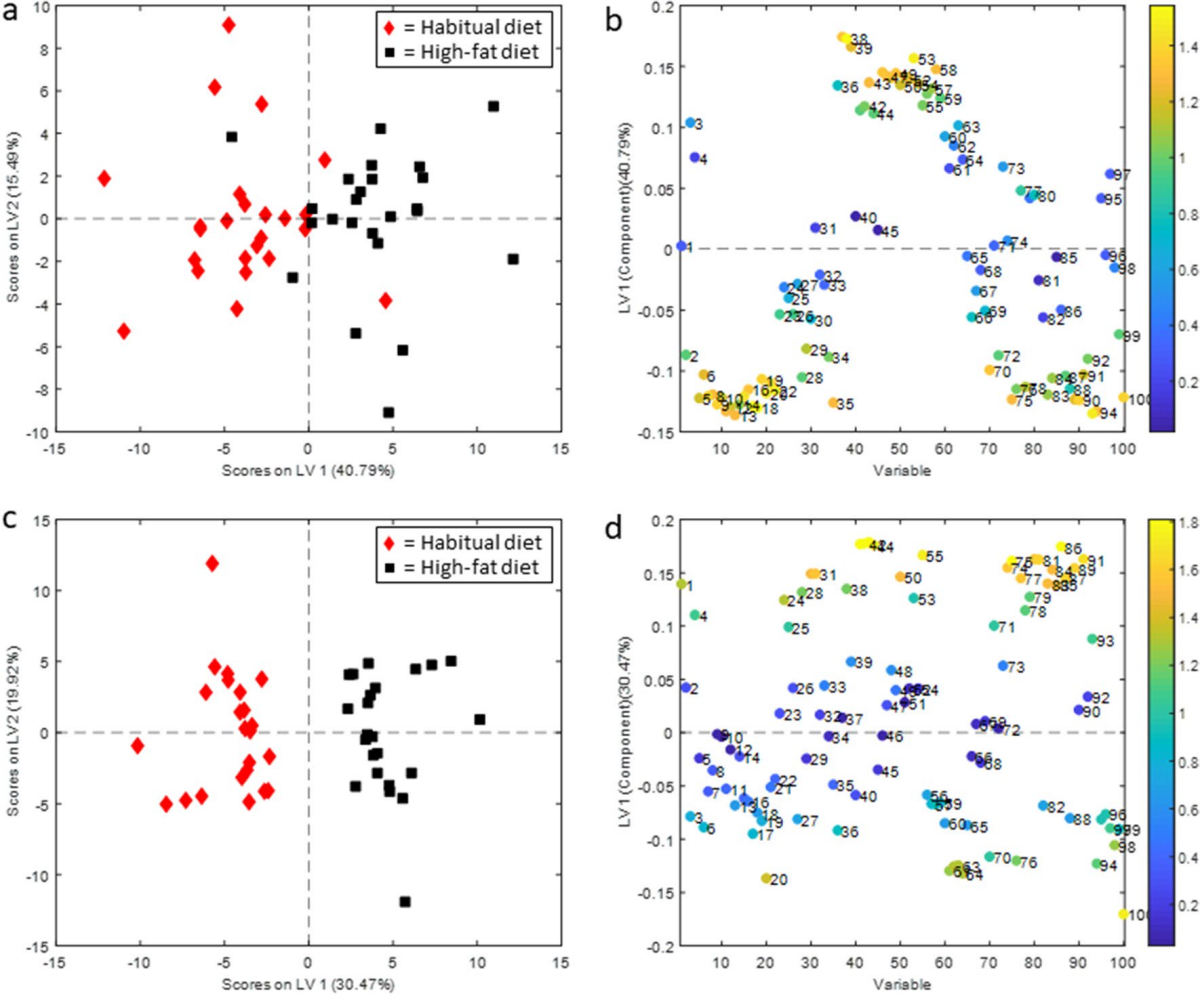
Scores and loading plots from multilevel partial least squares discriminant analyses for discriminating lipoprotein profiles at baseline (habitual diet) from those after 5 days of high-fat diet. (**a, b**) in fasted samples (**c, d**) in postprandial samples. LV = latent variable, A1 = apolipoprotein A1, A2 = apolipoprotein A2, AB = apolipoprotein B100, CH = cholesterol, TG = triglycerides, VLDL = very-low-density lipoprotein, FC = free cholesterol, PL = phospholipids, IDL = intermediate-density lipoprotein, LDL = low-density lipoprotein, HDL = High-density lipoprotein. 1: Total Serum A1, 2: Total Serum A2, 3: Total Serum AB, 4: Total Serum CH, 5: Total Serum TG, 6: VLDL AB, 7: VLDL CH, 8: VLDL FC, 9: VLDL PL, 10: VLDL TG, 11: VLDL1 CH, 12: VLDL-1 FC, 13: VLDL-1 PL, 14: VLDL-1 TG, 15: VLDL-2 CH, 16: VLDL-2 FC, 17: VLDL-2 PL, 18: VLDL-2 TG, 19: VLDL-3 CH, 20: VLDL-3 FC, 21: VLDL-3 PL, 22: VLDL-3 TG, 23: VLDL-4 CH, 24: VLDL-4 FC, 25: VLDL-4 PL, 26: VLDL-4 TG, 27: VLDL-5 CH, 28: VLDL-5 FC, 29: VLDL-5 PL, 30: VLDL-5 TG, 31: IDL AB, 32: IDL CH, 33: IDL FC, 34: IDL PL, 35: IDL TG, 36: LDL AB, 37: LDL CH, 38: LDL FC, 39: LDL PL, 40: LDL TG, 41: LDL-1 AB, 42: LDL-1 CH, 43: LDL-1 FC, 44: LDL-1 PL, 45: LDL-1 TG, 46: LDL-2 AB, 47: LDL-2 CH, 48: LDL-2 FC, 49: LDL-2 PL, 50: LDL-2 TG, 51: LDL-3 AB, 52: LDL-3 CH, 53: LDL-3 FC, 54: LDL-3 PL, 55: LDL-3 TG, 56: LDL-4 AB, 57: LDL-4 CH, 58: LDL-4 FC, 59: LDL-4 PL, 60: LDL-4 TG, 61: LDL-5 AB, 62: LDL-5 CH, 63: LDL-5 FC, 64: LDL-5 PL, 65: LDL-5 TG, 66: LDL-6 AB, 67: LDL-6 CH, 68: LDL-6 FC, 69: LDL-6 PL, 70: LDL-6 TG, 71: HDL A1, 72: HDL A2, 73: HDL CH, 74: HDL FC, 75: HDL PL, 76: HDL TG, 77: HDL-1 A1, 78: HDL-1 A2, 79: HDL-1 CH, 80: HDL-1 FC, 81: HDL-1 PL, 82: HDL-1 TG, 83: HDL-2 A1, 84: HDL-2 A2, 85: HDL-2 CH, 86: HDL-2 FC, 87: HDL-2 PL, 88: HDL-2 TG, 89: HDL-3 A1, 90: HDL-3 A2, 91: HDL-3 CH, 92: HDL-3 FC, 93: HDL-3 PL, 94: HDL-3 TG, 95: HDL-4 A1, 96: HDL-4 A2, 97: HDL-4 CH, 98, HDL-4 FC, 99 HDL-4 PL, 100: HDL-4 TG.

Supplementary Table 1 shows the correlation coefficients of total cholesterol, LDL, HDL, and triglycerides concentrations as measured by clinical biochemistry and NMR spectroscopy. Principal component analysis (PCA) trajectories from before to after 5 days of HFD showed systematic changes in lipoprotein profiles after initiating the HFD. In the fasting samples, there was a tendency of increase along PC1, while these changes were even more evident in the postprandial samples, with a clear increase along PC3 (Supplementary Figure 1). When removing the between-subject variation in a multilevel partial least squares discriminant analyses (PLS-DA), we observed substantial changes in the lipoprotein subfraction profiles after 5 days of HFD (Fig. 2).

**Figure 3:**
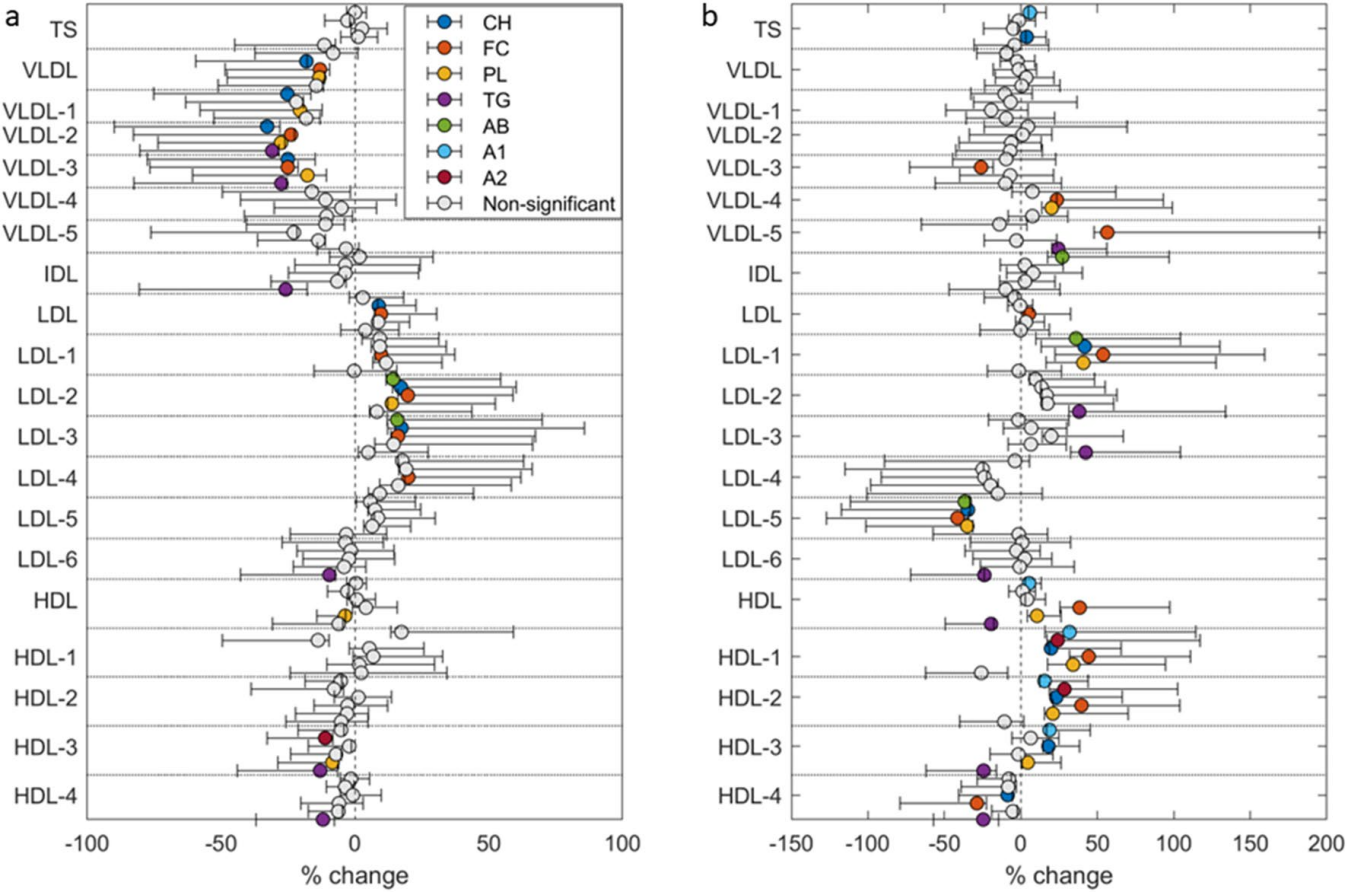
Change in lipoprotein subfractions from baseline to after 5 days of high-fat diet. In (**a**) fasted samples, and (**b**) postprandial samples. Symbols show median percentage change and error bars show interquartile range. TS = total serum, VLDL = very low-density lipoprotein, IDL = intermediate-density lipoprotein, LDL = low-density lipoprotein, HDL = high-density lipoprotein, CH = cholesterol, FC = free cholesterol, PL = phospholipids, TG = triglycerides, Apo-B = apolipoprotein B100, Apo-A1 = apolipoprotein A1, Apo-A2 = apolipoprotein A2.

The classification models separated samples from before to after 5 days of HFD with high classification accuracy (79% and 100% accuracy for fasting and postprandial samples, respectively). In the fasting samples, increased concentrations of several LDL-related variables and decreased concentrations of VLDL- and HDL-related variables were evident after HFD. The postprandial samples showed some deviations from the fasting samples, with reduced concentrations of variables related to LDL-5 and increased concentrations of several HDL-related variables (Fig. 2). Univariate analyses further confirmed significant changes after 5 days of HFD. The HFD induced significant (*q* < 0.05) changes in 31 of the 100 lipid variables in the fasting samples (Supplementary Table 2) and in 41 variables in the postprandial samples (Supplementary Table 3). Figure 3 shows the percentage change in all lipid subfraction variables from before to after the HFD. Fasting total serum VLDL cholesterol concentrations were reduced by ~ 25% (*q* = 0.039), with significant reductions in cholesterol only in the larger VLDL particles (VLDL-1–3). The HFD also decreased enrichment of triglycerides in VLDL-2 and VLDL-3, as well as in IDL, LDL-6, HDL-3, and HDL-4 in the fasting samples (Supplementary Table 2). VLDL profiles changed differently in the postprandial samples, showing increased free cholesterol concentration in the smaller VLDL particles (VLDL-4 and VLDL-5), as well as elevated triglycerides in VLDL-5 (Supplementary Table 3). In the postprandial samples, there was also increased enrichment of triglycerides in some LDL subfractions (LDL-2 and LDL-3), otherwise the enrichment of triglycerides in subfractions showed a similar pattern as in the fasted samples.

**Figure 4:**
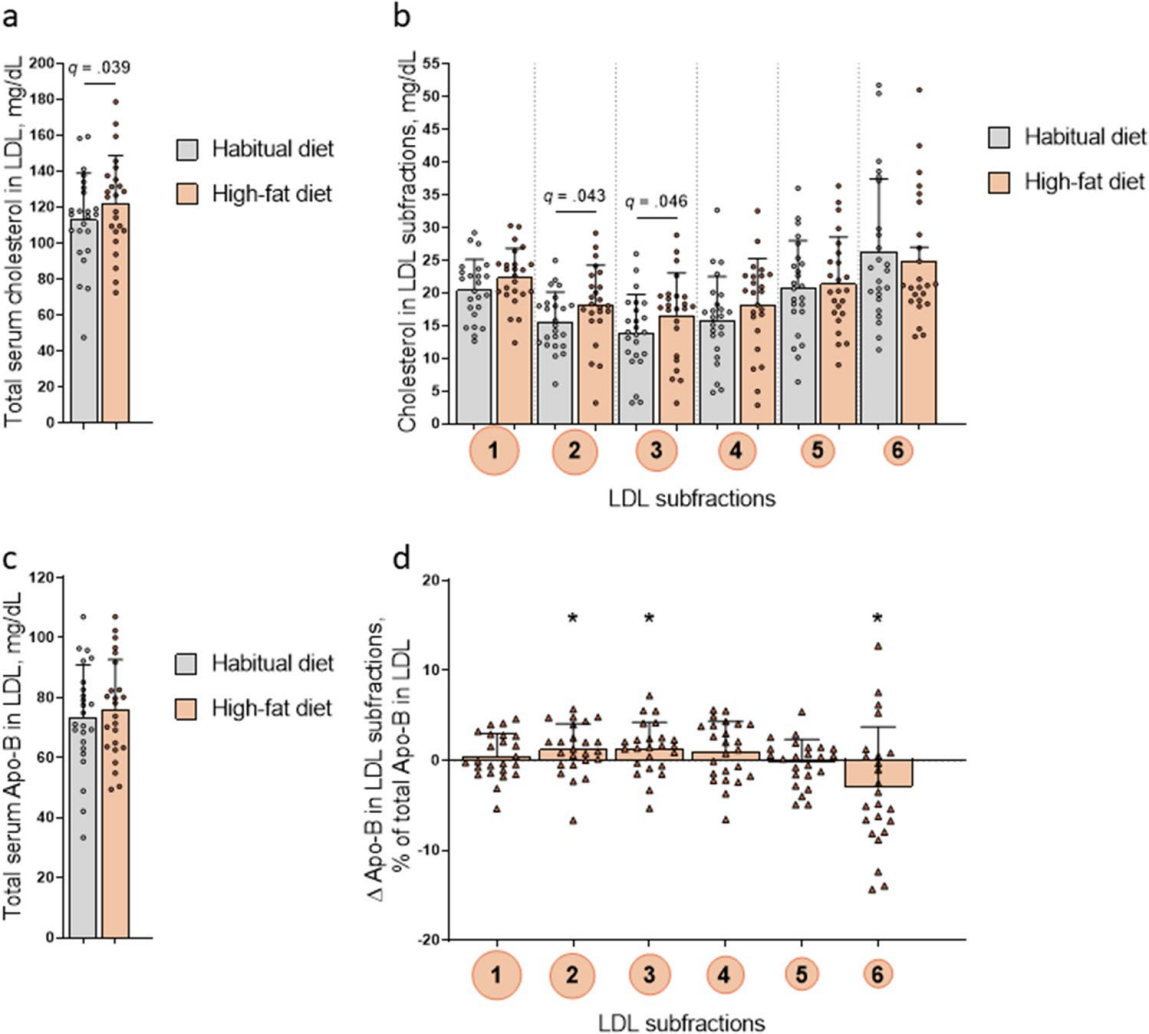
Cholesterol and Apolipoprotein-B100 (Apo-B) in LDL measured in the fasted state. Data from participants (*n* = 24) before (habitual diet) and after 5 days of high-fat diet. (**a**) Total serum cholesterol, (**b**) Cholesterol in LDL subfraction 1–6, (**c**) Total serum Apo-B, (**d**) Change in Apo-B in LDL subfraction 1–6, as percentage of total Apo-B in LDL, after 5 days of high-fat diet. Bars show means, error bars are SD, and symbols show individual values. *p < 0.05.

The HFD increased total fasting serum LDL cholesterol concentration, due to increased cholesterol in larger LDL particles, with significant increases in LDL-2 and LDL-3 (Fig. 4). The HFD induced a change in the distribution of fasting Apo-B concentrations in small versus large LDL subfractions, with increased concentrations in LDL-2 and LDL-3, and a concomitant decrease in LDL-6. These changes in Apo-B distribution within LDL subfractions were seen without any significant change in total LDL Apo-B (Fig. 4).

In the postprandial samples, total serum concentrations of cholesterol and Apo-A1 increased after the HFD (Supplementary Table 3). There was no change in total serum LDL cholesterol concentration in the postprandial samples, but significantly increased LDL-1 cholesterol and reduced LDL-5 cholesterol concentrations after 5 days of HFD (Supplementary Figure 2). Several other alterations in subfractions were evident postprandially after the HFD, with increased Apo-B concentrations in IDL and LDL-1, increased concentrations of free cholesterol and phospholipids in LDL-1, as well as triglyceride enrichment in LDL-2 and LDL-3. Although total serum HDL cholesterol concentration did not change significantly in the postprandial samples after 5 days of HFD, cholesterol concentrations in HDL-1–3 were elevated, whereas the opposite was true for the smallest HDL subfraction (HDL-4). Triglycerides enrichment in the smallest HDL particles (HDL-3–4) was reduced after 5 days of HFD (Supplementary Table 3).

**Figure 5:**
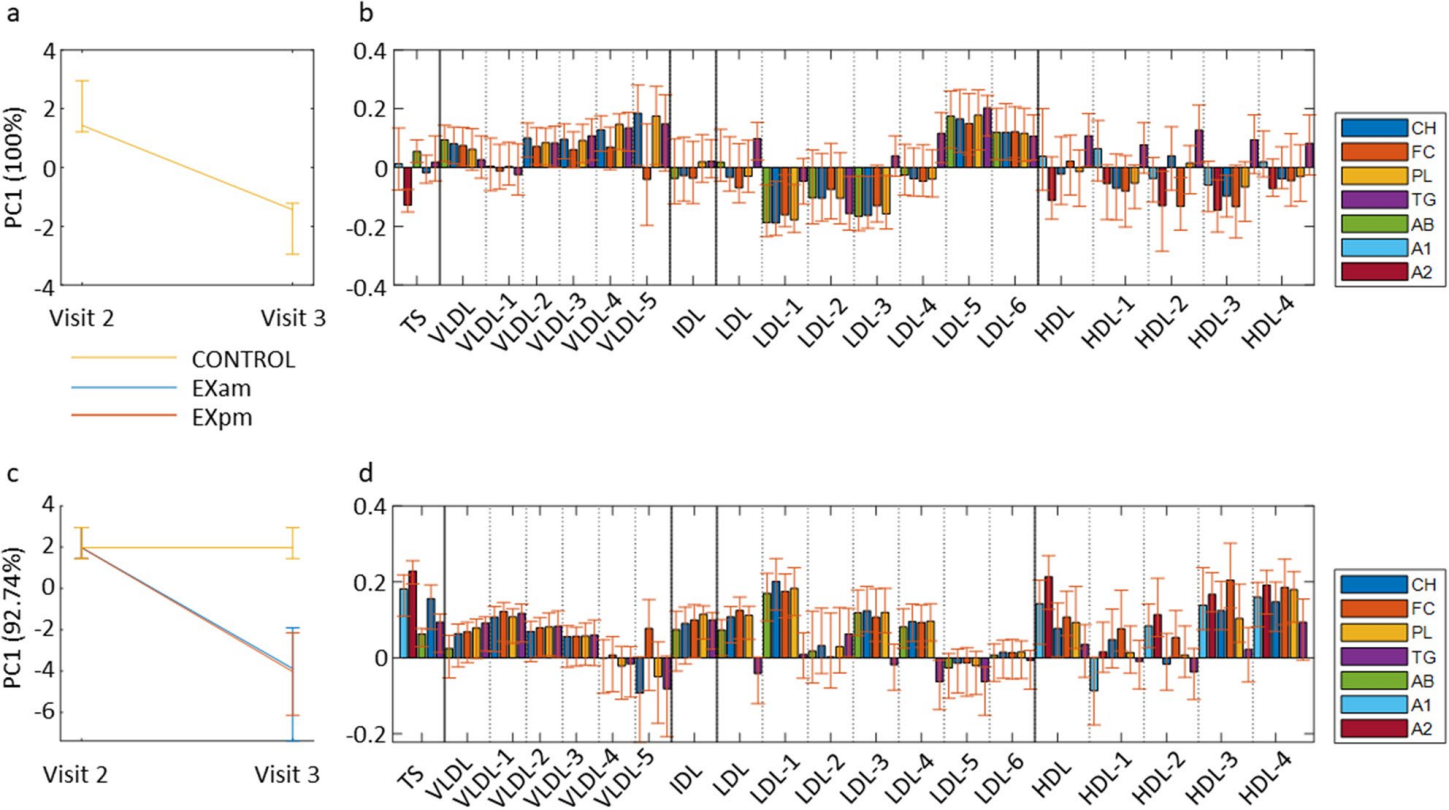
Changes after continued high-fat diet with and without exercise training. Scores and loading plots from repeated measures ANOVA simultaneous component analysis for discriminating lipoprotein profiles after exercise/no exercise for 5 days (Visit 3) from after 5 days of high-fat diet (Visit 2), in the fasted state. (**a**) Scores for changes between Visit 2 and Visit 3 in the control group (CONTROL), (**b**) Loadings for changes between Visit 2 and Visit 3 in the control group, (**c**) Scores for changes after morning exercise (EXam) and evening exercise (EXpm) between Visit 2 and Visit 3, compared with changes in the control group, (**d**), Loadings for changes after morning exercise (EXam) and evening exercise (EXpm) between Visit 2 and Visit 3, compared with changes in the control group. CH = cholesterol, FC = free cholesterol, PL = phospholipids, TG = triglycerides, AB = Apolipoprotein-B100, A1 = Apolipoprotein A-1, A2 = Apolipoprotein A-2.

When comparing the effect of continued HFD from 5 days (at Visit 2) to 11 days (Visit 3) we observed a decreased PC1 score at the last assessment (Fig. 5). Continued HFD for 11, compared with 5 days, induced an increase in the variables with negative loadings and a decrease in variables with positive loadings.

Despite no further elevation of total serum LDL cholesterol, cholesterol concentration in the larger LDLs (LDL-1–4) increased, while it decreased in the smaller LDLs (LDL-5–6) after continued HFD. Most of the HDL-related variables increased, except for triglycerides which decreased in all subfractions (Supplementary Table 4). Corresponding development in postprandial samples are shown in Supplementary Figure 3 and Supplementary Table 5.

### Changes in lipoprotein profiles after exercise training

The development in lipoprotein profile between after 5 days of HFD to after 11 days deviated clearly from the changes in CONTROL in the exercise groups, with similar development over time in EXam and EXpm (Fig. 5). In agreement with the repeated measures ANOVA simultaneous component (RM-ASCA +) analysis, univariate analyses showed a significant (*q* < 0.05) decrease in 20 of the lipid variables after EXam and in 24 after EXpm (15 of these in common) (Supplementary Table 4) in fasting blood. For those variables that changed significantly only in one of the exercise trained groups, the changes in both exercise groups were in the same direction (albeit not statistically significant in the other exercise group). Supplementary Figure 3 shows the development in postprandial lipoprotein profiles.

As also evident from the RM-ASCA + analysis, total serum cholesterol and Apo-A2 concentrations decreased in both exercise trained groups, compared with CONTROL (Supplementary Table 4). Univariate analysis revealed that EXam decreased the fasting concentrations of free cholesterol, phospholipids, and triglycerides in VLDL-1, as well as total serum Apo-A1, and concentration of phospholipids in IDL (all *q* < 0.05). Even if there were no significant reduction in total LDL cholesterol concentration in either of the exercise groups after adjusting for multiple comparisons, EXpm decreased the total fasting concentration of LDL free cholesterol (Supplementary Table 4). EXpm additionally affected several LDL-related variables, with reductions in fasting cholesterol concentrations in LDL-1 (*q* = 0.002), LDL-3 (*q* = 0.044), and LDL-4 (*q* = 0.013), whereas EXam only reduced LDL-1 cholesterol concentrations (*q* = 0.020) (Supplementary Table 4).

Independent of time-of-day, exercise training induced several changes in fasting HDL subfraction concentrations, without a statistically significant change in total serum HDL cholesterol concentration (Supplementary Table 4). Exercise training affected the smaller HDL particles (HDL-3 and HDL-4), with lower cholesterol concentrations in these particles after both EXam (*q* = 0.040 for both HDL-3 and HDL-4) and EXpm (*q* = 0.032 for HDL-3 and *q* = 0.034 for HDL-4) at study completion, compared with CONTROL. As evident from both the RM-ASCA + analysis and univariate analyses, exercise had less effect on the postprandial samples (Supplementary Figure 3, Supplementary Table 5). There were no statistically significant univariate differences in any fasting or postprandial lipoprotein subfraction variables between the EXam and EXpm (Supplementary Table 6).

## Discussion

We determined the effect of consumption of an HFD and exercise training performed in the morning or in the evening on circulating lipoprotein subfractions in men with overweight/obesity. We report that 5 days of HFD induced substantial changes in lipoprotein subfraction profiles, with alterations in VLDL, IDL, LDL, and HDL subfractions. Based on previous evidence of associations between lipoprotein subfractions and risk of cardiometabolic diseases^6,7,16–21^, we interpret the effect of the HFD on lipoprotein subfractions as mostly beneficial for cardiometabolic health. Daily exercise training for 5 days also induced distinct and favourable changes in lipoprotein subfraction profiles, with no clear difference between morning and evening exercise.

Consumption of an HFD for 5 days reduced the total fasting concentrations of cholesterol, free cholesterol, and phospholipids in VLDL, as well as cholesterol and triglycerides in several VLDL subfractions. Higher levels of large VLDL particles are associated with insulin resistance^16^ and incident type 2 diabetes^17^, independent of established risk factors such as circulating glucose and insulin concentrations. Furthermore, cholesterol in VLDL explained 40% of the excess risk of myocardial infarction associated with obesity in 29,010 individuals from the Copenhagen General Population Study^18^. In our study, consumption of the HFD primarily impacted the larger VLDL particles (VLDL-1–3), with no significant changes in VLDL-4 and VLDL-5. Fasting concentrations of VLDL-1 phospholipids were decreased after the HFD, with a tendency (*q* = 0.060) of decreased free cholesterol in VLDL-1. Streese and colleagues reported that both VLDL-1 phospholipids and free cholesterol concentrations were inversely associated with retinal arterio-to-venular diameter ratio, an independent measure of cardiovascular outcomes^19^. There were no effects of the HFD on main VLDL composition in postprandial samples after the HFD, but some of the subfraction components changed (Fig. 3). The timing of blood collection after a meal has implications on VLDL concentrations^22^, but in our study the timing of these collections was standardised on measurement days. We found that exercise training undertaken in the morning, but not in the evening, further decreased the concentrations of VLDL-1 free cholesterol, phospholipids, and triglycerides. There was also a tendency (*q* = 0.055) of reduced total triglycerides in VLDL after morning exercise training. The beneficial effect of exercise training on large VLDL particles is in agreement with a recent study reporting an inverse correlation between cardiorespiratory fitness (maximum oxygen uptake) and several VLDL subfractions (e.g., VLDL-1 free cholesterol, VLDL-1 phospholipids, and VLDL-1 triglycerides)^23^. It has been suggested that high intensity exercise is necessary to decrease the hepatic release of VLDL, and lower VLDL concentrations were only associated with a moderate-to-high intensity, and not low-intensity, aerobic physical activity in 509 participants with increased risk of impaired glucose regulation in the Walking Away from Diabetes study^24^. The exercise training protocol in the current study included three high-intensity interval training sessions, which may explain the beneficial effect of exercise on VLDL subfractions.

The increase in total serum cholesterol concentrations in LDL after 5 days of HFD was due to increased concentrations of cholesterol in the larger LDL subfractions, with no changes in small, dense LDL particles. We found no increase in the fasting concentrations of Apo-B, an indirect measure of LDL particle number, in total serum or in LDL. There was increased concentrations of Apo-B in larger LDL particles (LDL-2 and LDL-3), again revealing a shift towards larger, more buoyant LDL particles after the HFD. These findings are in line with a systematic review and meta-analysis showing an overall trend for an increase in the larger subclasses of LDL and a decrease in the smaller, more dense LDL subclasses, after carbohydrate-restricted dietary interventions^11^. The circulation time of smaller LDL particles is longer than that of large LDL particles, with small dense LDL particles being more susceptible to atherogenic modifications, including glycation and oxidation^25^. Indeed, several studies have shown a strong association between small dense LDL particles and incidence of cardiovascular disease^6,20,21^. For example, larger LDL particles showed no association with future coronary heart disease events in the Atherosclerosis Risk in Communities (ARIC) study, whereas the concentration of small-density LDL cholesterol predicted later incidence of such disease, independent of traditional cardiovascular risk factors in this large cohort study with 11,419 participants^21^.

We found that exercise training mostly affected large LDL particles, with reductions in fasting Apo-B, cholesterol, free cholesterol, and phospholipids concentrations in LDL-1 after both morning and evening exercise. There were additional reductions in some LDL-3 and LDL-4 subfractions after evening exercise training. These findings, in part, contrast with the results of a meta-analysis of 10 exercise interventions lasting 20–26 weeks which showed that large LDL particles increased and small dense LDL particles decreased after endurance exercise training^26^. The reason for these discrepant findings may be that the exercise intervention period in our study was short term (5 days).

HDL particles are heterogeneous in their size and composition and standard clinical measurement of HDL cholesterol concentrations is unable to capture this diversity. The findings of recent studies indicate that the inverse associations of cholesterol in HDL particles and incident type 2 diabetes and ASCVD are limited to large and medium subclasses^7,27–30^. In fact, some studies show that concentrations of smaller HDL particles are associated with *higher* risk of developing type 2 diabetes^28,30^. Diets high in fat (41–62% of total energy intake, TEI) generally increase total HDL cholesterol^31^, but this effect may be mediated by a concomitant reduction in body mass^32^. In our study, there was no change in total body mass and no change in total HDL cholesterol after 5 days of HFD. However, the HFD induced significant reductions in several HDL-related variables. There was reduced triglyceride enrichment in HDL-3 and HDL-4, indicating a beneficial effect of HFD since triglyceride concentrations in these small HDL particles are associated with risk of myocardial infarction^7^. Moreover, triglycerides concentration in HDL, most prominently HDL-3, is associated with reduced microvascular health (retinal arterio-to-venular diameter ratio)^19^ and low cardiorespiratory fitness^23^, both of which are important predictors of ASCVD events and mortality^33–35^.

Exercise training also primarily affected the smallest HDL particles, producing reduced fasting concentrations of Apo-A1, Apo-A2, and free cholesterol in both HDL-3 and HDL-4, and additionally lower HDL-4 phospholipids concentrations, after both morning and evening exercise. These findings are in agreement with a previous study that reported reduced concentrations of small HDL particles after 4 days of daily exercise (20 min of moderate intensity endurance exercise) in sedentary but otherwise healthy men, despite no change in total HDL concentrations^36^.

Strengths of the current study include its randomised design, rigorous dietary control with all meals provided to the participants, along with prescribed times for eating, and supervised exercise sessions in the laboratory (i.e., little uncontrolled environmental stimuli). The comprehensive analyses of lipoprotein particle profile quantified using NMR spectroscopy, with examination of different lipoprotein attributes not measured in a standard lipid profile, is a major strength of our study. However, there is no standardised method to analyse lipoprotein subfractions, with different methods utilising different techniques for separation of subfractions, making it challenging to directly compare our results with those of others. The timing of blood collection post-exercise can affect circulating concentrations of lipoproteins. For example, decreased total plasma triglyceride concentrations and increased concentrations of cholesterol in HDL were reported 24 h after exercise, and lasted through 48 h, after a single session of moderate-intensity treadmill walking in physically inactive men^37^. Due to the design of our study, which aimed to compare the effect of morning versus evening exercise, there was a difference in the timing of biological sampling since the last exercise session (12 vs 24 h for fasting samples and 24 vs 36 h for postprandial samples). This is a limitation in our study and such discrepancy is inherent in any investigation of the effects of the time of day of exercise. We only included men in our study and the results cannot be generalised to women. Furthermore, the small sample size per group may limit the interpretation of the findings due to large variability between individuals for some of the variables.

Our results are from a short-term experimental intervention albeit with strict control of the participants’ dietery intake, and accordingly, our findings should not be taken as clinical recommendations. We acknowledge that clinical guidelines for primary prevention of ASCVD from the major cardiology associations (ESC and AHA/ACC), recommend to reduce the intake of saturated fats^38,39^. However, several meta-analyses and recent studies indicate that there is no clear association between intake of total fat, or saturated fat, and risk of ASCVD^8–10,40^. Indeed, there is a current spirited debate in the scientific community about the scientific basis for the concept that fat in general, and saturated fat in particular, causes ASCVD^41,42^.

Short-term consumption of a HFD in men with overweight/obesity induced marked alterations in lipoprotein subfraction profiles, including reductions in several large VLDL subfractions and reduced triglycerides concentrations in small HDL particles. Daily exercise while consuming the HFD, undertaken either in the morning or in the evening, resulted in a distinctive lipoprotein subfraction signature, compared with no exercise. The effect of exercise was particularly evident for the largest LDL particles, with lower concentrations of Apo-B, cholesterol, free cholesterol, and phospholipids in LDL-1, as well as for several subfractions in the smallest HDL particles. Taken collectively, we interpret the overall effects of both the HFD and exercise intervention on lipoprotein subfractions as beneficial for cardiovascular disease prevention.

## Methods

### Trial design and participants

This was a randomised trial with three parallel groups, undertaken at the St Patrick’s (Fitzroy, VIC) campus of the Australian Catholic University. To be eligible for inclusion, the participants had to fulfil the following criteria: male sex; aged 30–45 years; BMI 27.0–35.0 kg/m^2^; and sedentary lifestyle (< 150 min/week moderate-intensity exercise for > 3 months and sitting for > 5 h each day). The exclusion criteria were known cardiovascular disease or type 2 diabetes; major chronic illness that impairs mobility or eating/digestion; taking prescription medications (i.e., β-blockers, anti-arrhythmic drugs, statins, or insulin sensitising drugs); previous bariatric surgery; shift-work; smoking; strict dietary intake regimes (e.g., vegan, not regularly consuming three meals per day, actively trying to lose weight); or not being weight stable (± 5 kg) for the last 3 months. The experimental protocol and methodology for the trial are published previously^15^. In brief, all participants consumed an HFD for 11 days and after the first 5 days of HFD, participants were randomly allocated (1:1:1) to one of three groups: One group of participants did not exercise (CONTROL), while one group trained in the morning (EXam) and one group in the evening (EXpm) on days 6–10. We used a computer random number generator developed and administered by the Unit for Applied Clinical Research at the Norwegian University of Science and Technology to allocate participants into groups. The randomisation had varying block sizes, with the computer technician defining the first, the smallest, and the largest block. The researcher who enrolled the participants (T.M.) was unaware of the size of the blocks.

The trial was approved by the Human Research Ethics Committee of the Australian Catholic University (2016-254H) and was performed in accordance with the Decleration of Helsinki. The trial was registered with the Australian New Zealand Clinical Trials Registry (registration no. ACTRN12617000304336) on 27/02/17, prior to inclusion of the first participant. Participants provided written informed consent before participation.

### Experimental protocol and interventions

**Figure 6:**
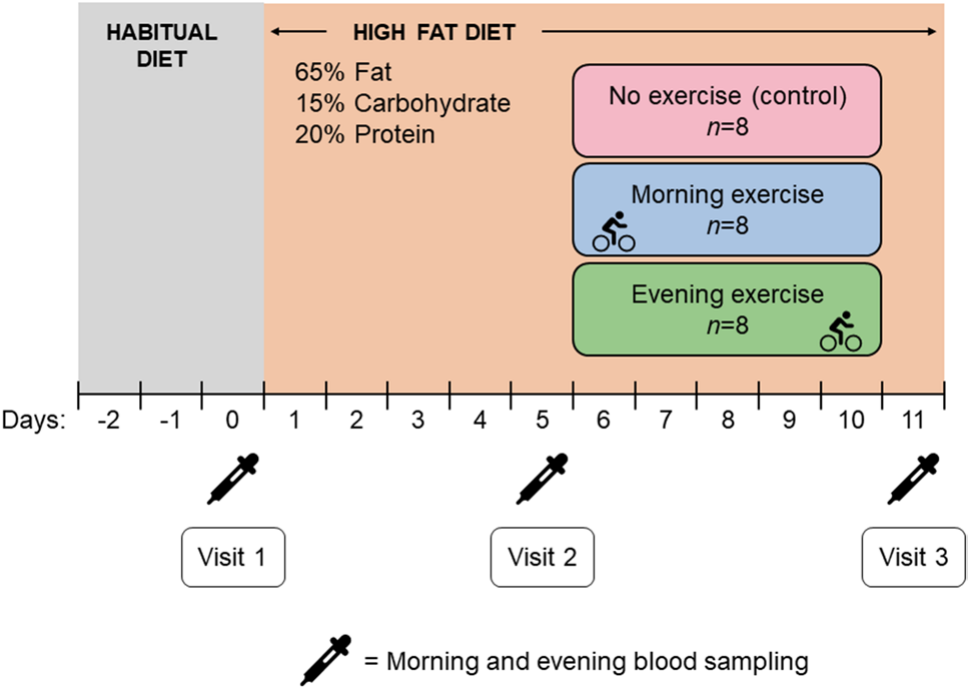
Experimental design. All participants consumed a high-fat diet for 11 days. Two groups of participants exercised daily on day 6–10, either in the morning (*n* = 8) or in the evening (*n* = 8), and one group of participants (*n* = 8) remained inactive during the whole study period (control group). Blood samples were obtained in the morning (fasted) and in the evening (postprandial) at baseline (Visit 1), after 5 days of high-fat diet (Visit 2), and at study completion (Visit 3).

Figure 6 shows an schematic of the experimental protocol. All participants consumed a HFD consisting of 65% TEI from fat, 15% TEI from carbohydrate and 20% TEI from protein for 11 days. The breakdown of the fat component of the diet was 52% saturated fat, 10% polyunsaturated fat and 38% monounsaturated fat (Supplementary Table 7). The HFD consisted of three pre-packed meals (breakfast, lunch, and dinner) each day, which were to be consumed at prescribed times (07:30, 13:00, and 19:00 h). Each of these meals contained 33.3% of TEI, with individualised TEI based on resting metabolic rate measurements^15^. Supplementary Table 8 shows examples of high-fat meals the participants ate. Participants could drink water, coffee/tea (without sugar/milk) ad libitum.

After the initial 5 days, two groups of participants exercised daily on days 6–10, either at 06:30 h (EXam) or at 18:30 h (EXpm), whereas one group of participants did not exercise (CONTROL). The exercise protocols were identical for the EXam and EXpm groups and consisted of a combination of high-intensity interval training and moderate-intensity continuous cycling. The high-intensity interval training sessions (completed on day 6, 8 and 10) consisted of a 10-min warm-up followed by ten 1-min work-bouts at 95–120% of individual peak power output, separated by 1-min low-intensity cycling. The moderate-intensity continuous sessions comprised cycling at 60–65% of individual peak power output for 40 min (on day 7) and 60 min (on day 9). In the meal following each exercise session, the participants consumed a 419 kJ snack, with the same macronutrient composition as in the HFD, to maintain energy balance. Participants allocated to CONTROL maintained their habitual activities of daily living.

### Blood sampling and clinical chemistry

We sampled venous blood before breakfast and after dinner, at baseline (Visit 1), after 5 days of HFD (Visit 2), and after a further 5 days on the HFD, either with daily exercise or no exercise (Visit 3) (Fig. 6). The participants fasted from 22:00 h the night before the blood sampling on all visits, with blood obtained between 07:15 and 07:45. The evening (postprandial) samples were obtained 34 (SD 7) minutes after eating dinner, between 19:20 and 20:00 h. The time since the last bout of exercise at Visit 3 was (by design) different between the EXam and EXpm group: For fasted samples this time was ⁓24 h for EXam and ⁓12 h for EXpm, and for postprandial samples the time was ⁓36 h for EXam and ⁓24 h for EXpm. Circulating concentrations of total cholesterol, HDL cholesterol, LDL cholesterol, and triglycerides were analysed in whole blood using Cobas b 101 (Roche Diagnostics Ltd, Switzerland), and have been reported previously^15^.

### Nuclear magnetic resonance spectroscopy and lipid subfraction quantification

Serum samples were kept at − 80 °C until shipment on dry ice to the NTNU MR Core Facility in Trondheim, Norway. The samples were thawed at room temperature prior to the NMR analysis. Serum (320 µL) was mixed with equal volume of buffer (20%D_2_O in 0.075 M Na_2_HPO_4_, 6 mM NaN_3_, 4.6 mM 3-(trimethylsilyl)propionic-2,2,3,3-tetradeutero-acid (TSP-d_4_), pH 7.4) and analysed in 5 mm tubes at 310 K. For 16 of the samples, the volume of serum was < 300 µL and these samples were analysed in 3 mm tubes, using 120 µL serum and buffer. Samples were analysed using a Bruker Avance III 600 MHz spectrometer (Bruker BioSpin GmbH, Germany), equipped with a BBI probe. Data acquisition and sample handling were automated (SampleJet with Icon-NMR on TopSpin 3.6). Lipoprotein subclassification was performed using Bruker BioSpin (Bruker IVDr Lipoprotein Subclass Analysis B.I.LISA™), based on one-dimensional NOESY NMR spectra^43^. This model provides information on concentrations of cholesterol, free cholesterol, phospholipids, and apolipoprotein A1 (Apo-A1), apolipoprotein A2 (Apo-A2) and apolipoprotein B-100 (Apo-B) in serum, as well as in each of the lipoprotein classes (very-low-density lipoprotein (VLDL), intermediate-density lipoprotein (IDL), LDL, and HDL). Each lipoprotein class was additionally subdivided into subfractions according to their density. VLDL was divided into VLDL 1–5, LDL into LDL 1–6, and HDL into HDL 1–4, with increasing density. In addition, their concentrations of cholesterol, free cholesterol, phospholipids, Apo-A1, Apo-A2, Apo-B, and triglycerides were estimated.

### Statistical analysis

We did not complete a formal sample size calculation for this study due to the exploratory nature of the research question. Fasted (morning) and postprandial (evening) blood samples were analysed separately. We calculated Pearson’s correlation coefficients between total cholesterol, LDL cholesterol, HDL cholesterol, and triglycerides measured by standard clinical chemistry and NMR spectroscopy in the fasted and postprandial samples. Data are expressed as means with standard deviation SD and estimates with 95% confidence intervals.

As a first step in the exploratory analysis, we performed a PCA comparing samples from baseline to after 5 days on the HFD. We then employed multilevel PLS-DA for supervised analysis^44^. In multilevel PLS-DA, we utilised the multilevel structure of the data to remove the between-subject variation, thereby focussing on the within-subject variation. Multilevel PLS-DA was validated by leave-one-patient-out cross-validation, and the resulting model was orthogonalized for increased interpretation. The loading plots of the orthogonalized PLS-DA were coloured according to the lipoprotein variable importance score (VIP score), which indicates how important each variable was for creating the discrimination model. The PCA and PLS-DA analyses were undertaken in Matlab R2018b using the PLS_Toolbox 8.7.2 (Eigenvector Research, Wenatchee, WA, USA), and variables were autoscaled before analysis.

We used RM-ASCA+^45^ to determine multivariate changes in lipoprotein profiles between EXam, EXpm, and CONTROL. In RM-ASCA+ the effect matrices resulting from univariate linear mixed models are analysed by PCA to assess overall effects. Linear mixed models were performed using time (visit 2 and visit 3) and the time*group interaction as fixed effects, while participant was used as random effect. Fixed effect variables were reference coded with visit 2 and CONTROL as reference for time and group, respectively. We analysed the time effect and time*group interactions separately in the RM-ASCA+ analysis, and the results are visualized as scores and loadings. Due to the reference coding, the time effect will represent time changes in CONTROL. The time*group interactions plots show how EXam and EXpm deviate from CONTROL. Non-parametric bootstrapping was used to construct 95% confidence intervals.

We also used univariate analyses to investigate each of the 100 lipoprotein subfraction variables. To determine the effect of 5 days of HFD on lipoprotein subclasses, we used paired samples t-tests, with adjustments for multiple comparisons using the Benjamini–Hochberg procedure^46^. For these comparisons, we consider adjusted *p-*values (*q*-values) < 0.05 to be statistically significant. To determine the between-group difference in lipoprotein subclasses after exercise in the morning, exercise in the evening, or no exercise, we used linear mixed models. These models were executed for each of the 100 lipid variables from the NMR spectroscopy as dependent variables, with adjustments for baseline values (the values at Visit 2, prior to the exercise intervention), with participant as random effect, and time and time*group interactions as fixed effects. We built separate models for the comparison of EXam and EXpm versus the control group, and for EXpm versus EXam. *P-*values were adjusted as described above.

## Supplementary Information

Supplementary Information 1.

Supplementary Information 2.

Supplementary Information 3.

Supplementary Information 4.

## Data Availability

The datasets generated during and/or analysed during the current study are available from the corresponding author on reasonable request.
