# Supplementary figures and images for: Effect of high-fat diet and morning or evening exercise on lipoprotein subfraction profiles: secondary analysis of a randomised trial
Source: Sci Rep. 2023 Mar 10;13:4008. doi: 10.1038/s41598-023-31082-0 (PMC10006421; doi:10.1038/s41598-023-31082-0)

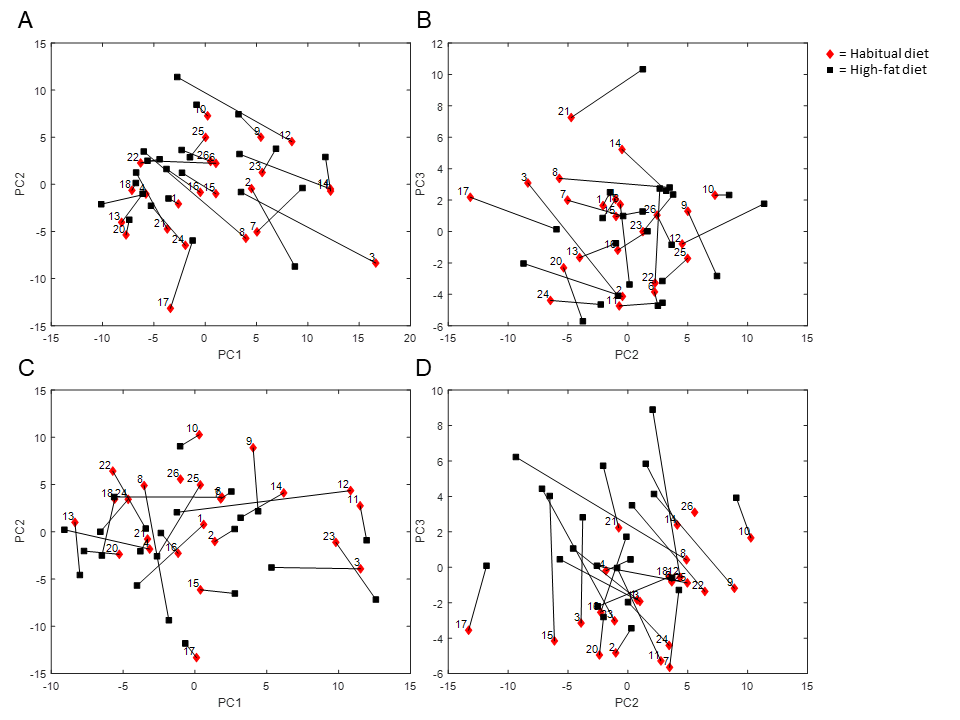

Supplement: Supplementary file 1 — Supplementary Information 1. [file 41598_2023_31082_MOESM1_ESM.tif]

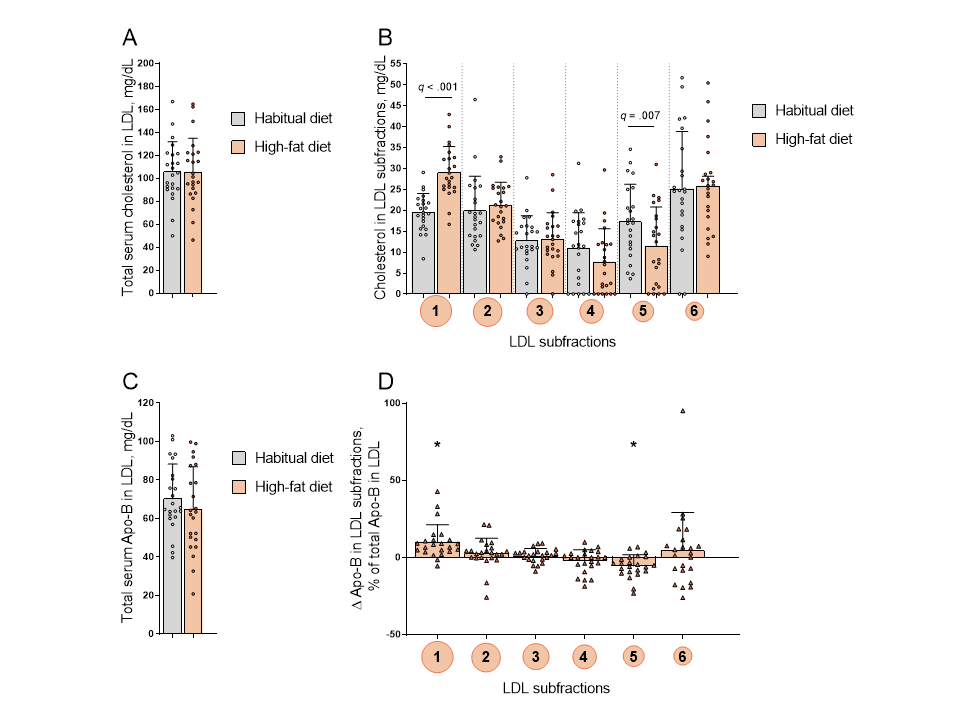

Supplement: Supplementary file 2 — Supplementary Information 2. [file 41598_2023_31082_MOESM2_ESM.tif]

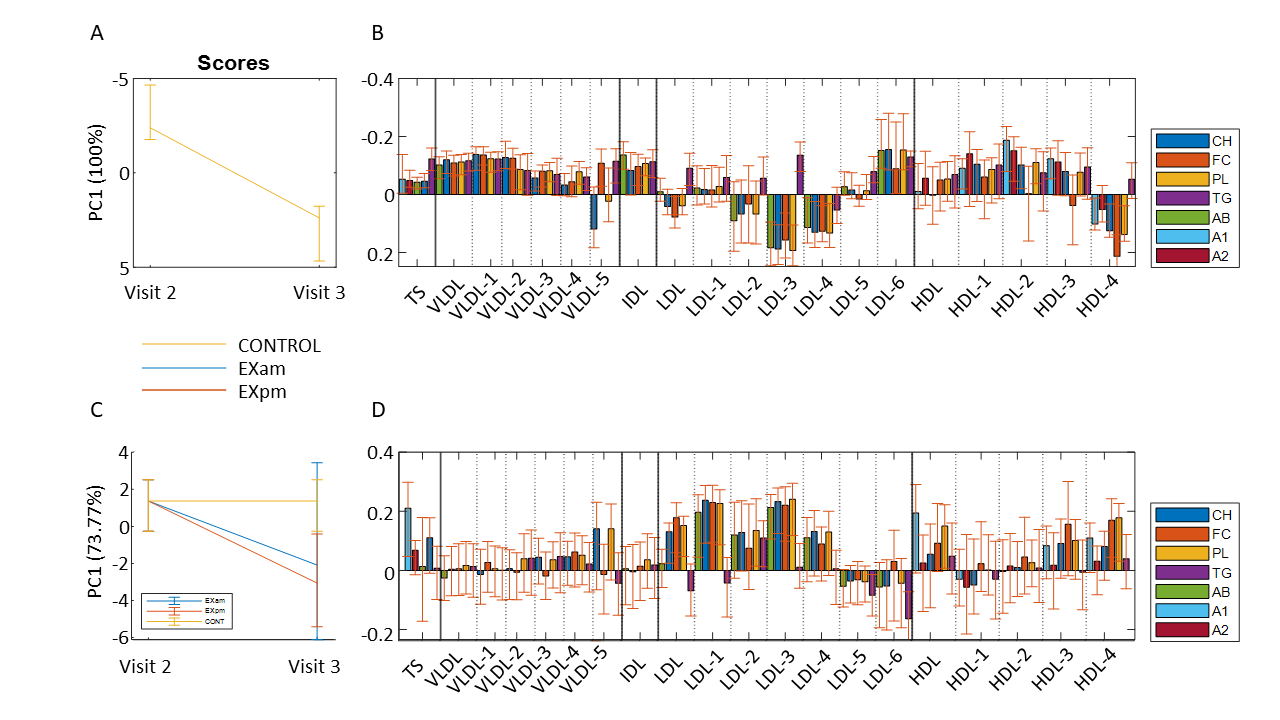

Supplement: Supplementary file 3 — Supplementary Information 3. [file 41598_2023_31082_MOESM3_ESM.tif]
