# Supplementary material for: Effect of high-fat diet and morning or evening exercise on lipoprotein subfraction profiles: secondary analysis of a randomised trial
Source: Sci Rep. 2023 Mar 10;13:4008. doi: 10.1038/s41598-023-31082-0 (PMC10006421; doi:10.1038/s41598-023-31082-0)
Supplement: Supplementary file 4 — Supplementary Information 4. [file 41598_2023_31082_MOESM4_ESM.docx]

**Supplementary material**

**Supplementary Table 1.** Correlation coefficients of lipids measured by clinical chemistry and NMR spectroscopy. Data are means ± standard deviation.

| **Lipid** | **Clinical Chemistry** | **NMR Spectroscopy** | **Pearson Correlation Coefficient** |
| --- | --- | --- | --- |
| ***Fasting samples*** |  |  |  |
| Total cholesterol, mmol/L | 4.66 ± 0.78 | 4.98 ± 0.89 | 0.97 |
| LDL-cholesterol, mmol/L | 3.04 ± 0.69 | 3.01 ± 0.69 | 0.94 |
| HDL-cholesterol, mmol/L | 1.03 ± 0.16 | 1.25 ± 0.14 | 0.86 |
| Triglycerides, mmol/L | 1.26 ± 0.65 | 1.32 ± 0.68 | 0.99 |
| ***Postprandial samples*** |  |  |  |
| Total cholesterol, mmol/L | 4.81 ± 0.80 | 5.03 ± 0.96 | 0.96 |
| LDL-cholesterol, mmol/L | 2.78 ± 0.72 | 2.70 ± 0.70 | 0.92 |
| HDL-cholesterol, mmol/L | 1.04 ± 0.16 | 1.24 ± 0.14 | 0.81 |
| Triglycerides, mmol/L | 2.13 ± 1.02 | 2.14 ± 0.97 | 0.95 |

Original values from the NMR spectroscopy data file were in mg/dL and converted to mmol/L; for total cholesterol, HDL-cholesterol, and LDL-cholesterol by multiplication by 0.0259; for triglycerides by 0.01129.

**Supplementary Table 2.** Fasting lipid main- and subfractions and their contents of lipids and lipoproteins before and after five days of high-fat diet in men with overweight/obesity. Data are observed means ± SD and observed change. *P* values from paired samples t-tests. *Q* values are from Benjamini-Hochberg correction. Variable number (#) refer to the same numbers as used in PLSDA plots.

|  | **#** | **Habitual diet**  **(*n* = 24)** | **High-fat diet**  **(*n* =24)** | **Change** | ***p* value** | ***q* value** |
| --- | --- | --- | --- | --- | --- | --- |
| **Main Parameters** | | | | | | |
| Total Apo-A1, mg/dL | 1 | 129.3 ± 8.7 | 130.0 ± 9.0 | 0.8 ± 6.6 | 0.582 | 0.638 |
| Total Apo-A2, mg/dL | 2 | 31.8 ± 3.0 | 30.8 ± 2.6 | -1.1 ± 2.7 | 0.069 | 0.114 |
| Total Apo B100, mg/dL | 3 | 89.9 ± 20.7 | 92.2 ± 18.7 | 2.3 ± 10.0 | 0.273 | 0.368 |
| Total Cholesterol, mg/dL | 4 | 197.5 ± 31.2 | 198.7 ± 31.2 | 1.2 ± 16.0 | 0.727 | 0.765 |
| Total Triglycerides, mg/dL | 5 | 144.2 ± 65.9 | 115.9 ± 56.0 | -28.2 ± 54.7 | **0.019** | 0.053 |
| **VLDL Main and Subfraction Composition** | | | | | | |
| VLDL Apo-B100, mg/dL | 6 | 9.5 ± 3.8 | 8.0 ± 3.2 | -1.4 ± 2.7 | **0.016** | 0.050 |
| VLDL Cholesterol, mg/dL | 7 | 23.9 ± 12.5 | 18.0 ± 9.6 | -6.0 ± 9.6 | **0.006** | **0.039** |
| VLDL Free Cholesterol, mg/dL | 8 | 11.1 ± 5.0 | 9.0 ± 4.3 | -2.1 ± 3.7 | **0.010** | **0.043** |
| VLDL Phospholipids, mg/dL | 9 | 26.1 ± 10.8 | 21.1 ± 9.7 | -5.0 ± 7.9 | **0.005** | **0.039** |
| VLDL Triglycerides, mg/dL | 10 | 99.9 ± 49.3 | 80.3 ± 43.3 | -19.6 ± 38.9 | **0.021** | 0.054 |
| VLDL-1 Cholesterol, mg/dL | 11 | 9.4 ± 5.8 | 6.6 ± 4.6 | -2.8 ± 5.0 | **0.012** | **0.043** |
| VLDL-1 Free Cholesterol, mg/dL | 12 | 3.7 ± 2.1 | 2.8 ± 1.9 | -0.9 ± 1.8 | **0.024** | 0.060 |
| VLDL-1 Phospholipids, mg/dL | 13 | 9.2 ± 4.9 | 7.0 ± 4.3 | -2.2 ± 4.0 | **0.011** | **0.043** |
| VLDL-1 Triglycerides, mg/dL | 14 | 55.7 ± 30.5 | 44.7 ± 27.4 | -11.0 ± 24.9 | **0.041** | 0.089 |
| VLDL-2 Cholesterol, mg/dL | 15 | 3.8 ± 2.5 | 2.4 ± 1.8 | -1.4 ± 2.1 | **0.003** | **0.039** |
| VLDL-2 Free Cholesterol, mg/dL | 16 | 1.6 ± 1.1 | 1.0 ± 0.8 | -0.6 ± 0.9 | **0.007** | **0.039** |
| VLDL-2 Phospholipids, mg/dL | 17 | 4.4 ± 2.3 | 3.1 ± 1.8 | -1.3 ± 1.8 | **0.002** | **0.039** |
| VLDL-2 Triglycerides, mg/dL | 18 | 16.8 ± 9.2 | 11.5 ± 7.4 | -5.3 ± 7.4 | **0.002** | **0.039** |
| VLDL-3 Cholesterol, mg/dL | 19 | 4.0 ± 2.6 | 2.8 ± 1.9 | -1.2 ± 2.0 | **0.006** | **0.039** |
| VLDL-3 Free Cholesterol, mg/dL | 20 | 1.8 ± 1.2 | 1.2 ± 0.9 | -0.6 ± 0.9 | **0.004** | **0.039** |
| VLDL-3 Phospholipids, mg/dL | 21 | 4.4 ± 2.2 | 3.3 ± 1.7 | -1.1 ± 1.7 | **0.005** | **0.039** |
| VLDL-3 Triglycerides, mg/dL | 22 | 12.8 ± 7.2 | 9.0 ± 5.6 | -3.8 ± 5.6 | **0.003** | **0.039** |
| VLDL-4 Cholesterol, mg/dL | 23 | 5.1 ± 2.3 | 4.5 ± 2.0 | -0.7 ± 1.7 | 0.077 | 0.127 |
| VLDL-4 Free Cholesterol, mg/dL | 24 | 1.9 ± 1.0 | 1.8 ± 0.8 | -0.1 ± 0.8 | 0.374 | 0.480 |
| VLDL-4 Phospholipids, mg/dL | 25 | 4.7 ± 1.7 | 4.3 ± 1.5 | -0.4 ± 1.3 | 0.194 | 0.277 |
| VLDL-4 Triglycerides, mg/dL | 26 | 8.9 ± 3.5 | 8.0 ± 3.2 | -1.0 ± 2.6 | 0.081 | 0.131 |
| VLDL-5 Cholesterol, mg/dL | 27 | 1.4 ± 0.4 | 1.3 ± 0.5 | -0.1 ± 0.4 | 0.233 | 0.324 |
| VLDL-5 Free Cholesterol, mg/dL | 28 | 0.7 ± 0.4 | 0.6 ± 0.4 | -0.1 ± 0.3 | 0.067 | 0.114 |
| VLDL-5 Phospholipids, mg/dL | 29 | 1.8 ± 0.5 | 1.6 ± 0.5 | -0.2 ± 0.4 | **0.026** | 0.063 |
| VLDL-5 Triglycerides, mg/dL | 30 | 2.8 ± 0.6 | 2.7 ± 0.6 | -0.1 ± 0.5 | 0.192 | 0.277 |
| **IDL Main and Subfraction Composition** | | | | | | |
| IDL Apo-B100, mg/dL | 31 | 5.1 ± 1.8 | 5.2 ± 1.4 | 0.1 ± 1.3 | 0.680 | 0.724 |
| IDL Cholesterol, mg/dL | 32 | 12.5 ± 5.7 | 12.0 ± 4.2 | -0.5 ± 3.8 | 0.501 | 0.589 |
| IDL Free Cholesterol, mg/dL | 33 | 3.6 ± 1.6 | 3.4 ± 1.3 | -0.2 ± 1.1 | 0.482 | 0.581 |
| IDL Phospholipids, mg/dL | 34 | 8.1 ± 3.7 | 7.0 ± 3.2 | -1.1 ± 2.5 | 0.052 | 0.100 |
| IDL Triglycerides, mg/dL | 35 | 15.7 ± 11.0 | 10.5 ± 9.2 | -5.2 ± 9.3 | **0.012** | **0.043** |
| **LDL Main and Subfraction Composition** | | | | | | |
| LDL Apo-B100, mg/dL | 36 | 73.5 ± 17.5 | 76.2 ± 16.5 | 2.7 ± 8.7 | 0.145 | 0.220 |
| LDL Cholesterol, mg/dL | 37 | 113.4 ± 25.6 | 122.3 ± 26.4 | 8.9 ± 14.7 | **0.007** | **0.039** |
| LDL Free Cholesterol, mg/dL | 38 | 32.1 ± 6.8 | 35.4 ± 6.8 | 3.3 ± 4.3 | **0.001** | **0.039** |
| LDL Phospholipids, mg/dL | 39 | 63.0 ± 12.0 | 66.7 ± 12.4 | 3.7 ± 7.0 | **0.017** | 0.051 |
| LDL Triglycerides, mg/dL | 40 | 17.1 ± 5.0 | 17.2 ± 4.2 | 0.1 ± 3.9 | 0.907 | 0.917 |
| LDL-1 Apo-B100, mg/dL | 41 | 10.8 ± 2.2 | 11.7 ± 2.1 | 0.9 ± 2.1 | 0.054 | 0.101 |
| LDL-1 Cholesterol, mg/dL | 42 | 20.5 ± 4.6 | 22.5 ± 4.4 | 1.9 ± 4.5 | **0.043** | 0.090 |
| LDL-1 Free Cholesterol, mg/dL | 43 | 5.9 ± 1.3 | 6.6 ± 1.2 | 0.7 ± 1.2 | **0.012** | **0.043** |
| LDL-1 Phospholipids, mg/dL | 44 | 11.8 ± 2.3 | 12.7 ± 2.2 | 0.9 ± 2.2 | 0.059 | 0.103 |
| LDL-1 Triglycerides, mg/dL | 45 | 4.7 ± 1.7 | 4.7 ± 1.6 | 0.0 ± 1.4 | 0.908 | 0.917 |
| LDL-2 Apo-B100, mg/dL | 46 | 8.9 ± 2.1 | 10.2 ± 2.8 | 1.3 ± 2.2 | **0.008** | **0.039** |
| LDL-2 Cholesterol, mg/dL | 47 | 15.7 ± 4.4 | 18.3 ± 5.9 | 2.6 ± 4.5 | **0.009** | **0.043** |
| LDL-2 Free Cholesterol, mg/dL | 48 | 4.8 ± 1.2 | 5.6 ± 1.7 | 0.9 ± 1.4 | **0.007** | **0.039** |
| LDL-2 Phospholipids, mg/dL | 49 | 8.7 ± 2.2 | 10.0 ± 2.8 | 1.3 ± 2.2 | **0.008** | **0.039** |
| LDL-2 Triglycerides, mg/dL | 50 | 1.8 ± 0.6 | 2.0 ± 0.5 | 0.2 ± 0.4 | **0.019** | 0.053 |
| LDL-3 Apo-B100, mg/dL | 51 | 8.4 ± 3.0 | 9.8 ± 3.2 | 1.4 ± 2.6 | **0.013** | **0.043** |
| LDL-3 Cholesterol, mg/dL | 52 | 14.0 ± 5.8 | 16.7 ± 6.4 | 2.7 ± 5.0 | **0.014** | **0.046** |
| LDL-3 Free Cholesterol, mg/dL | 53 | 4.2 ± 1.4 | 5.1 ± 1.6 | 0.9 ± 1.3 | **0.002** | **0.039** |
| LDL-3 Phospholipids, mg/dL | 54 | 8.0 ± 2.8 | 9.2 ± 3.1 | 1.2 ± 2.5 | **0.021** | 0.054 |
| LDL-3 Triglycerides, mg/dL | 55 | 2.3 ± 0.5 | 2.4 ± 0.4 | 0.1 ± 0.3 | **0.046** | 0.094 |
| LDL-4 Apo-B100, mg/dL | 56 | 10.0 ± 4.0 | 11.3 ± 4.2 | 1.3 ± 2.9 | **0.042** | 0.089 |
| LDL-4 Cholesterol, mg/dL | 57 | 16.0 ± 6.5 | 18.3 ± 7.0 | 2.3 ± 5.0 | **0.035** | 0.079 |
| LDL-4 Free Cholesterol, mg/dL | 58 | 4.6 ± 1.4 | 5.3 ± 1.6 | 0.7 ± 1.2 | **0.012** | **0.043** |
| LDL-4 Phospholipids, mg/dL | 59 | 8.8 ± 3.3 | 9.9 ± 3.5 | 1.0 ± 2.5 | 0.056 | 0.102 |
| LDL-4 Triglycerides, mg/dL | 60 | 2.0 ± 0.9 | 2.1 ± 0.9 | 0.2 ± 0.6 | 0.249 | 0.341 |
| LDL-5 Apo-B100, mg/dL | 61 | 14.5 ± 5.1 | 14.8 ± 4.8 | 0.3 ± 2.9 | 0.619 | 0.666 |
| LDL-5 Cholesterol, mg/dL | 62 | 20.8 ± 7.2 | 21.5 ± 7.1 | 0.7 ± 4.0 | 0.418 | 0.522 |
| LDL-5 Free Cholesterol, mg/dL | 63 | 5.5 ± 1.7 | 5.8 ± 1.6 | 0.3 ± 1.1 | 0.219 | 0.308 |
| LDL-5 Phospholipids, mg/dL | 64 | 11.2 ± 3.5 | 11.4 ± 3.5 | 0.2 ± 2.0 | 0.583 | 0.628 |
| LDL-5 Triglycerides, mg/dL | 65 | 2.6 ± 1.2 | 2.5 ± 1.1 | -0.1 ± 0.8 | 0.447 | 0.547 |
| LDL-6 Apo-B100, mg/dL | 66 | 21.6 ± 9.2 | 19.9 ± 8.2 | -1.7 ± 5.7 | 0.151 | 0.226 |
| LDL-6 Cholesterol, mg/dL | 67 | 26.4 ± 11.0 | 25.0 ± 9.7 | -1.4 ± 6.3 | 0.290 | 0.381 |
| LDL-6 Free Cholesterol, mg/dL | 68 | 6.4 ± 2.6 | 6.2 ± 2.1 | -0.2 ± 1.5 | 0.550 | 0.632 |
| LDL-6 Phospholipids, mg/dL | 69 | 14.4 ± 5.3 | 13.5 ± 4.8 | -0.9 ± 3.1 | 0.170 | 0.251 |
| LDL-6 Triglycerides, mg/dL | 70 | 4.7 ± 1.9 | 3.9 ± 1.5 | -0.8 ± 1.3 | **0.008** | **0.039** |
| **HDL Main and Subfraction Composition** | | | | | | |
| HDL Apo-A1, mg/dL | 71 | 129.6 ± 9.8 | 130.6 ± 9.4 | 0.9 ± 7.1 | 0.536 | 0.624 |
| HDL Apo-A2, mg/dL | 72 | 32.4 ± 3.0 | 31.3 ± 2.6 | -1.1 ± 2.7 | 0.057 | 0.102 |
| HDL Cholesterol, mg/dL L | 73 | 48.0 ± 5.8 | 48.9 ± 5.5 | 0.9 ± 3.9 | 0.284 | 0.379 |
| HDL Free Cholesterol, mg/dL | 74 | 10.2 ± 1.6 | 10.5 ± 1.5 | 0.3 ± 1.3 | 0.358 | 0.465 |
| HDL Phospholipids, mg/dL | 75 | 64.5 ± 7.2 | 61.4 ± 6.8 | -3.1 ± 5.4 | **0.010** | **0.043** |
| HDL Triglycerides, mg/dL | 76 | 8.9 ± 2.2 | 8.0 ± 2.2 | -0.9 ± 2.2 | 0.051 | 0.100 |
| HDL-1 Apo-A1, mg/dL | 77 | 12.2 ± 6.1 | 13.9 ± 4.4 | 1.7 ± 4.6 | 0.087 | 0.139 |
| HDL-1 Apo-A2, mg/dL | 78 | 1.4 ± 0.5 | 1.0 ± 0.5 | -0.3 ± 0.6 | **0.015** | 0.062 |
| HDL-1 Cholesterol, mg/dL | 79 | 10.2 ± 4.0 | 10.6 ± 2.8 | 0.5 ± 2.9 | 0.448 | 0.547 |
| HDL-1 Free Cholesterol, mg/dL | 80 | 2.7 ± 0.7 | 2.9 ± 0.7 | 0.2 ± 0.6 | 0.138 | 0.212 |
| HDL-1 Phospholipids, mg/dL | 81 | 11.4 ± 3.9 | 11.3 ± 3.2 | -0.1 ± 2.8 | 0.851 | 0.877 |
| HDL-1 Triglycerides, mg/dL | 82 | 1.9 ± 0.7 | 1.8 ± 0.8 | -0.0 ± 0.6 | 0.737 | 0.768 |
| HDL-2 Apo-A1, mg/dL | 83 | 14.9 ± 2.5 | 13.9 ± 1.9 | -1.0 ± 2.3 | **0.033** | 0.076 |
| HDL-2 Apo-A2, mg/dL | 84 | 2.7 ± 0.8 | 2.3 ± 0.6 | -0.4 ± 0.9 | **0.033** | 0.076 |
| HDL-2 Cholesterol, mg/dL | 85 | 6.0 ± 1.5 | 6.0 ± 1.2 | 0.0 ± 1.1 | 0.971 | 0.971 |
| HDL-2 Free Cholesterol, mg/dL | 86 | 1.4 ± 0.4 | 1.4 ± 0.4 | 0.0 ± 0.3 | 0.488 | 0.581 |
| HDL-2 Phospholipids, mg/dL | 87 | 9.5 ± 2.5 | 8.7 ± 1.9 | -0.7 ± 1.9 | 0.067 | 0.114 |
| HDL-2 Triglycerides, mg/dL | 88 | 1.3 ± 0.4 | 1.2 ± 0.4 | -0.1 ± 0.4 | 0.130 | 0.202 |
| HDL-3 Apo-A1, mg/dL | 89 | 23.4 ± 4.2 | 21.6 ± 3.2 | -1.8 ± 3.4 | **0.017** | 0.050 |
| HDL-3 Apo-A2, mg/dL | 90 | 6.0 ± 1.3 | 5.2 ± 0.9 | -0.8 ± 1.3 | **0.005** | **0.039** |
| HDL-3 Cholesterol, mg/dL | 91 | 8.8 ± 1.5 | 8.2 ± 1.3 | -0.5 ± 1.1 | **0.021** | 0.054 |
| HDL-3 Free Cholesterol, mg/dL | 92 | 2.0 ± 0.4 | 1.8 ± 0.4 | -0.2 ± 0.4 | 0.052 | 0.100 |
| HDL-3 Phospholipids, mg/dL | 93 | 14.2 ± 2.8 | 12.6 ± 2.3 | -1.7 ± 2.3 | **0.002** | **0.039** |
| HDL-3 Triglycerides, mg/dL | 94 | 2.0 ± 0.7 | 1.6 ± 0.5 | -0.3 ± 0.6 | **0.010** | **0.043** |
| HDL-4 Apo-A1, mg/dL | 95 | 80.1 ± 7.7 | 80.9 ± 8.3 | 0.9 ± 7.2 | 0.556 | 0.632 |
| HDL-4 Apo-A2, mg/dL | 96 | 21.9 ± 2.4 | 21.7 ± 2.5 | -0.2 ± 2.0 | 0.587 | 0.638 |
| HDL-4 Cholesterol, mg/dL | 97 | 21.7 ± 2.3 | 22.0 ± 2.7 | 0.3 ± 2.3 | 0.572 | 0.638 |
| HDL-4 Free Cholesterol, mg/dL | 98 | 4.0 ± 0.8 | 3.9 ± 0.6 | -0.1 ± 0.8 | 0.386 | 0.489 |
| HDL-4 Phospholipids, mg/dL | 99 | 29.4 ± 3.1 | 28.1 ± 3.0 | -1.3 ± 3.1 | 0.054 | 0.101 |
| HDL-4 Triglycerides, mg/dL | 100 | 4.1 ± 1.0 | 3.5 ± 0.8 | -0.5 ± 0.8 | **0.006** | **0.039** |

Apo-A1 = apolipoprotein A1, Apo-A2 = apolipoprotein A2, Apo-B = apolipoprotein B100, VLDL = very-low-density lipoprotein, IDL = intermediate-density lipoprotein, LDL = low-density lipoprotein, HDL = High-density lipoprotein.

**Supplementary Table 3.** Evening (postprandial) lipid main- and subfractions and their contents of lipids and lipoproteins before and after five days of high-fat diet in men with overweight/obesity. Data are observed means ± SD. *P* values from paired samples t-tests. *Q* values are from Benjamini-Hochberg correction. Variable number (#) refer to the same numbers as used in PLSDA plots.

|  | **#** | **Habitual diet**  **(*n* = 23)** | **High-fat diet**  **(*n* = 23)** | **Change** | ***p* value** | ***q* value** |
| --- | --- | --- | --- | --- | --- | --- |
| **Main Parameters** | | | | | | |
| Total Apo-A1, mg/dL | 1 | 128.7 ± 9.9 | 136.5 ± 9.7 | 7.8 ± 10.0 | **0.001** | **0.005** |
| Total Apo-A2, mg/dL | 2 | 31.2 ± 4.0 | 31.7 ± 4.1 | 0.5 ± 4.1 | 0.542 | 0.695 |
| Total Apo B100, mg/dL | 3 | 89.4 ± 19.6 | 84.7 ± 24.9 | -4.7 ± 12.5 | 0.087 | 0.167 |
| Total Cholesterol, mg/dL | 4 | 194.6 ± 33.4 | 205.2 ± 39.6 | 10.7 ± 19.2 | **0.014** | **0.039** |
| Total Triglycerides, mg/dL | 5 | 212.1 ± 92.9 | 206.2 ± 91.9 | -5.9 ± 72.9 | 0.700 | 0.803 |
| **VLDL Main and Subfraction Composition** | | | | | | |
| VLDL Apo-B100, mg/dL | 6 | 10.8 ± 4.2 | 9.8 ± 4.1 | -1.0 ± 2.4 | 0.058 | 0.120 |
| VLDL Cholesterol, mg/dL | 7 | 29.5 ± 14.6 | 28.0 ± 12.5 | -1.5 ± 9.2 | 0.445 | 0.610 |
| VLDL Free Cholesterol, mg/dL | 8 | 13.1 ± 5.9 | 12.6 ± 5.7 | -0.6 ± 3.7 | 0.466 | 0.630 |
| VLDL Phospholipids, mg/dL | 9 | 30.5 ± 12.6 | 30.2 ± 13.0 | -0.3 ± 9.1 | 0.862 | 0.909 |
| VLDL Triglycerides, mg/dL | 10 | 142.6 ± 65.1 | 141.6 ± 69.1 | -1.0 ± 50.0 | 0.925 | 0.944 |
| VLDL-1 Cholesterol, mg/dL | 11 | 15.5 ± 8.7 | 14.4 ± 7.6 | -1.1 ± 5.5 | 0.343 | 0.497 |
| VLDL-1 Free Cholesterol, mg/dL | 12 | 6.4 ± 3.3 | 6.3 ± 2.9 | -0.1 ± 2.3 | 0.908 | 0.936 |
| VLDL-1 Phospholipids, mg/dL | 13 | 15.0 ± 7.8 | 13.5 ± 6.7 | -1.5 ± 5.0 | 0.156 | 0.262 |
| VLDL-1 Triglycerides, mg/dL | 14 | 94.1 ± 49.4 | 90.0 ± 48.1 | -4.1 ± 33.3 | 0.559 | 0.699 |
| VLDL-2 Cholesterol, mg/dL | 15 | 4.4 ± 2.9 | 4.0 ± 2.1 | -0.5 ± 2.5 | 0.387 | 0.544 |
| VLDL-2 Free Cholesterol, mg/dL | 16 | 2.1 ± 1.3 | 1.8 ± 1.0 | -0.2 ± 1.0 | 0.268 | 0.403 |
| VLDL-2 Phospholipids, mg/dL | 17 | 5.3 ± 2.8 | 4.4 ± 2.2 | -0.9 ± 2.3 | 0.062 | 0.124 |
| VLDL-2 Triglycerides, mg/dL | 18 | 20.9 ± 11.1 | 17.9 ± 9.7 | -3.0 ± 9.9 | 0.157 | 0.262 |
| VLDL-3 Cholesterol, mg/dL | 19 | 4.3 ± 2.6 | 3.6 ± 2.2 | -0.7 ± 2.0 | 0.096 | 0.179 |
| VLDL-3 Free Cholesterol, mg/dL | 20 | 2.3 ± 1.4 | 1.7 ± 1.3 | -0.6 ± 0.8 | **0.001** | **0.005** |
| VLDL-3 Phospholipids, mg/dL | 21 | 4.9 ± 2.2 | 4.5 ± 2.1 | -0.5 ± 2.0 | 0.270 | 0.403 |
| VLDL-3 Triglycerides, mg/dL | 22 | 14.4 ± 7.6 | 12.8 ± 7.7 | -1.6 ± 8.3 | 0.367 | 0.525 |
| VLDL-4 Cholesterol, mg/dL | 23 | 4.4 ± 2.5 | 4.6 ± 2.2 | 0.2 ± 1.7 | 0.553 | 0.699 |
| VLDL-4 Free Cholesterol, mg/dL | 24 | 1.9 ± 0.9 | 2.4 ± 1.1 | 0.5 ± 0.7 | **0.001** | **0.005** |
| VLDL-4 Phospholipids, mg/dL | 25 | 4.3 ± 1.7 | 5.4 ± 1.9 | 1.1 ± 2.0 | **0.017** | **0.044** |
| VLDL-4 Triglycerides, mg/dL | 26 | 8.5 ± 3.2 | 9.0 ± 4.3 | 0.5 ± 3.8 | 0.523 | 0.688 |
| VLDL-5 Cholesterol, mg/dL | 27 | 1.6 ± 0.9 | 1.3 ± 0.5 | -0.3 ± 0.9 | 0.117 | 0.209 |
| VLDL-5 Free Cholesterol, mg/dL | 28 | 0.8 ± 0.4 | 1.2 ± 0.7 | 0.4 ± 0.6 | **0.005** | **0.018** |
| VLDL-5 Phospholipids, mg/dL | 29 | 2.0 ± 0.7 | 1.9 ± 0.5 | -0.1 ± 0.6 | 0.655 | 0.777 |
| VLDL-5 Triglycerides, mg/dL | 30 | 2.8 ± 0.7 | 3.3 ± 0.7 | 0.5 ± 0.5 | **<0.001** | **0.001** |
| **IDL Main and Subfraction Composition** | | | | | | |
| IDL Apo-B100, mg/dL | 31 | 5.5 ± 1.7 | 7.2 ± 2.3 | 1.8 ± 1.7 | **<0.001** | **<0.001** |
| IDL Cholesterol, mg/dL | 32 | 13.9 ± 6.4 | 14.4 ± 7.4 | 0.5 ± 4.9 | 0.624 | 0.761 |
| IDL Free Cholesterol, mg/dL | 33 | 4.3 ± 1.9 | 4.7 ± 2.1 | 0.3 ± 1.4 | 0.248 | 0.387 |
| IDL Phospholipids, mg/dL | 34 | 10.7 ± 4.6 | 10.8 ± 4.7 | 0.1 ± 3.0 | 0.849 | 0.909 |
| IDL Triglycerides, mg/dL | 35 | 27.7 ± 16.2 | 25.4 ± 15.3 | -2.3 ± 12.8 | 0.392 | 0.544 |
| **LDL Main and Subfraction Composition** | | | | | | |
| LDL Apo-B100, mg/dL | 36 | 70.7 ± 17.6 | 64.9 ± 21.9 | -5.8 ± 12.4 | **0.037** | 0.081 |
| LDL Cholesterol, mg/dL | 37 | 106.0 ± 25.8 | 105.8 ± 29.1 | -0.2 ± 15.2 | 0.948 | 0.951 |
| LDL Free Cholesterol, mg/dL | 38 | 28.1 ± 7.8 | 31.4 ± 6.8 | 3.3 ± 5.1 | **0.005** | **0.018** |
| LDL Phospholipids, mg/dL | 39 | 59.1 ± 12.7 | 61.0 ± 13.5 | 1.9 ± 7.0 | 0.206 | 0.327 |
| LDL Triglycerides, mg/dL | 40 | 18.4 ± 4.4 | 17.6 ± 5.6 | -0.8 ± 5.5 | 0.500 | 0.666 |
| LDL-1 Apo-B100, mg/dL | 41 | 10.2 ± 2.2 | 14.5 ± 2.8 | 4.3 ± 2.9 | **<0.001** | **<0.001** |
| LDL-1 Cholesterol, mg/dL | 42 | 19.5 ± 4.5 | 29.1 ± 6.1 | 9.6 ± 6.7 | **<0.001** | **<0.001** |
| LDL-1 Free Cholesterol, mg/dL | 43 | 5.5 ± 1.4 | 8.6 ± 1.7 | 3.1 ± 2.0 | **<0.001** | **<0.001** |
| LDL-1 Phospholipids, mg/dL | 44 | 11.2 ± 2.3 | 16.3 ± 3.1 | 5.1 ± 3.5 | **<0.001** | **<0.001** |
| LDL-1 Triglycerides, mg/dL | 45 | 5.9 ± 2.5 | 5.6 ± 2.2 | -0.3 ± 3.0 | 0.661 | 0.777 |
| LDL-2 Apo-B100, mg/dL | 46 | 11.1 ± 4.4 | 11.2 ± 2.5 | 0.1 ± 5.4 | 0.951 | 0.951 |
| LDL-2 Cholesterol, mg/dL | 47 | 20.0 ± 8.2 | 21.3 ± 5.4 | 1.3 ± 10.0 | 0.538 | 0.695 |
| LDL-2 Free Cholesterol, mg/dL | 48 | 5.7 ± 2.3 | 6.6 ± 1.7 | 0.8 ± 2.8 | 0.172 | 0.278 |
| LDL-2 Phospholipids, mg/dL | 49 | 10.7 ± 4.0 | 11.7 ± 2.8 | 1.0 ± 5.0 | 0.326 | 0.480 |
| LDL-2 Triglycerides, mg/dL | 50 | 1.9 ± 0.7 | 2.8 ± 0.7 | 0.9 ± 0.9 | **<0.001** | **0.001** |
| LDL-3 Apo-B100, mg/dL | 51 | 7.8 ± 3.1 | 7.9 ± 3.3 | 0.1 ± 3.1 | 0.873 | 0.909 |
| LDL-3 Cholesterol, mg/dL | 52 | 12.8 ± 5.8 | 13.2 ± 6.3 | 0.4 ± 5.6 | 0.765 | 0.841 |
| LDL-3 Free Cholesterol, mg/dL | 53 | 3.6 ± 1.6 | 4.2 ± 1.4 | 0.7 ± 1.3 | **0.026** | 0.061 |
| LDL-3 Phospholipids, mg/dL | 54 | 7.4 ± 2.9 | 7.6 ± 3.0 | 0.2 ± 2.7 | 0.680 | 0.791 |
| LDL-3 Triglycerides, mg/dL | 55 | 2.1 ± 0.6 | 2.8 ± 0.5 | 0.8 ± 0.5 | **<0.001** | **<0.001** |
| LDL-4 Apo-B100, mg/dL | 56 | 7.0 ± 5.2 | 5.2 ± 5.0 | -1.8 ± 5.1 | 0.105 | 0.191 |
| LDL-4 Cholesterol, mg/dL | 57 | 11.0 ± 8.4 | 7.7 ± 7.8 | -3.3 ± 8.0 | 0.059 | 0.120 |
| LDL-4 Free Cholesterol, mg/dL | 58 | 3.2 ± 2.1 | 2.3 ± 2.0 | -0.9 ± 2.2 | 0.047 | 0.101 |
| LDL-4 Phospholipids, mg/dL | 59 | 6.1 ± 4.4 | 4.4 ± 4.2 | -1.7 ± 4.2 | 0.065 | 0.128 |
| LDL-4 Triglycerides, mg/dL | 60 | 1.4 ± 0.9 | 1.1 ± 1.1 | -0.3 ± 1.0 | 0.128 | 0.225 |
| LDL-5 Apo-B100, mg/dL | 61 | 12.2 ± 6.0 | 8.3 ± 6.5 | -3.9 ± 5.4 | **0.002** | **0.007** |
| LDL-5 Cholesterol, mg/dL | 62 | 17.3 ± 8.8 | 11.5 ± 9.3 | -5.8 ± 7.9 | **0.002** | **0.007** |
| LDL-5 Free Cholesterol, mg/dL | 63 | 4.3 ± 2.2 | 2.7 ± 2.3 | -1.6 ± 2.1 | **0.002** | **0.006** |
| LDL-5 Phospholipids, mg/dL | 64 | 9.6 ± 4.2 | 6.6 ± 4.7 | -3.1 ± 4.0 | **0.001** | **0.006** |
| LDL-5 Triglycerides, mg/dL | 65 | 2.3 ± 1.2 | 2.0 ± 1.4 | -0.4 ± 1.2 | 0.163 | 0.267 |
| LDL-6 Apo-B100, mg/dL | 66 | 22.7 ± 10.7 | 22.1 ± 9.6 | -0.6 ± 9.8 | 0.754 | 0.838 |
| LDL-6 Cholesterol, mg/dL | 67 | 25.2 ± 13.6 | 25.9 ± 10.6 | 0.7 ± 12.7 | 0.799 | 0.868 |
| LDL-6 Free Cholesterol, mg/dL | 68 | 6.8 ± 2.7 | 6.6 ± 2.5 | -0.2 ± 2.8 | 0.706 | 0.803 |
| LDL-6 Phospholipids, mg/dL | 69 | 14.4 ± 6.4 | 14.8 ± 5.4 | 0.4 ± 6.0 | 0.731 | 0.821 |
| LDL-6 Triglycerides, mg/dL | 70 | 4.7 ± 2.1 | 3.6 ± 2.2 | -1.0 ± 1.9 | **0.016** | **0.041** |
| **HDL Main and Subfraction Composition** | | | | | | |
| HDL Apo-A1, mg/dL | 71 | 129.5 ± 10.0 | 134.7 ± 10.0 | 5.2 ± 9.8 | **0.018** | **0.041** |
| HDL Apo-A2, mg/dL | 72 | 31.8 ± 4.2 | 31.7 ± 4.6 | -0.2 ± 4.5 | 0.867 | 0.909 |
| HDL Cholesterol, mg/dL L | 73 | 47.4 ± 6.1 | 49.0 ± 5.8 | 1.6 ± 6.5 | 0.260 | 0.401 |
| HDL Free Cholesterol, mg/dL | 74 | 10.0 ± 3.0 | 13.6 ± 3.3 | 3.6 ± 3.6 | **<0.001** | **0.001** |
| HDL Phospholipids, mg/dL | 75 | 68.2 ± 8.9 | 75.7 ± 7.6 | 7.4 ± 4.9 | **<0.001** | **<0.001** |
| HDL Triglycerides, mg/dL | 76 | 11.4 ± 4.2 | 9.4 ± 2.7 | -2.0 ± 3.2 | **0.006** | **0.018** |
| HDL-1 Apo-A1, mg/dL | 77 | 16.3 ± 8.7 | 22.6 ± 7.5 | 6.3 ± 6.3 | **<0.001** | **0.001** |
| HDL-1 Apo-A2, mg/dL | 78 | 1.7 ± 0.9 | 2.2 ± 0.8 | 0.5 ± 0.9 | **0.009** | **0.026** |
| HDL-1 Cholesterol, mg/dL | 79 | 11.3 ± 4.3 | 14.0 ± 4.0 | 2.7 ± 4.4 | **0.008** | **0.025** |
| HDL-1 Free Cholesterol, mg/dL | 80 | 2.9 ± 1.1 | 4.0 ± 1.1 | 1.1 ± 1.1 | **<0.001** | **0.001** |
| HDL-1 Phospholipids, mg/dL | 81 | 13.5 ± 5.4 | 18.1 ± 5.0 | 4.6 ± 4.2 | **<0.001** | **<0.001** |
| HDL-1 Triglycerides, mg/dL | 82 | 2.9 ± 1.9 | 2.4 ± 1.0 | -0.6 ± 1.6 | 0.097 | 0.179 |
| HDL-2 Apo-A1, mg/dL | 83 | 16.5 ± 3.2 | 18.9 ± 2.8 | 2.4 ± 2.7 | **<0.001** | **0.001** |
| HDL-2 Apo-A2, mg/dL | 84 | 3.3 ± 1.1 | 4.4 ± 1.2 | 1.1 ± 1.2 | **<0.001** | **0.001** |
| HDL-2 Cholesterol, mg/dL | 85 | 7.2 ± 2.5 | 8.6 ± 2.1 | 1.4 ± 1.5 | **<0.001** | **0.001** |
| HDL-2 Free Cholesterol, mg/dL | 86 | 1.4 ± 0.5 | 1.9 ± 0.5 | 0.5 ± 0.4 | **<0.001** | **<0.001** |
| HDL-2 Phospholipids, mg/dL | 87 | 11.8 ± 4.0 | 14.5 ± 3.7 | 2.7 ± 2.4 | **<0.001** | **<0.001** |
| HDL-2 Triglycerides, mg/dL | 88 | 2.0 ± 1.1 | 1.8 ± 0.6 | -0.3 ± 0.8 | 0.136 | 0.234 |
| HDL-3 Apo-A1, mg/dL | 89 | 25.4 ± 4.6 | 29.2 ± 4.7 | 3.8 ± 3.5 | **<0.001** | **<0.001** |
| HDL-3 Apo-A2, mg/dL | 90 | 6.8 ± 1.6 | 7.0 ± 1.4 | 0.1 ± 1.3 | 0.632 | 0.762 |
| HDL-3 Cholesterol, mg/dL | 91 | 9.2 ± 1.7 | 10.4 ± 1.6 | 1.2 ± 1.0 | **<0.001** | **<0.001** |
| HDL-3 Free Cholesterol, mg/dL | 92 | 1.7 ± 0.6 | 1.8 ± 0.4 | 0.1 ± 0.6 | 0.600 | 0.740 |
| HDL-3 Phospholipids, mg/dL | 93 | 15.7 ± 3.2 | 17.0 ± 2.5 | 1.3 ± 2.3 | **0.011** | **0.031** |
| HDL-3 Triglycerides, mg/dL | 94 | 2.7 ± 1.1 | 2.1 ± 0.7 | -0.6 ± 0.9 | **0.009** | **0.026** |
| HDL-4 Apo-A1, mg/dL | 95 | 69.2 ± 14.1 | 63.6 ± 13.1 | -5.7 ± 11.8 | **0.031** | 0.071 |
| HDL-4 Apo-A2, mg/dL | 96 | 19.0 ± 4.4 | 16.9 ± 4.5 | -2.1 ± 4.4 | **0.031** | 0.071 |
| HDL-4 Cholesterol, mg/dL | 97 | 18.0 ± 4.5 | 15.6 ± 4.8 | -2.5 ± 4.5 | **0.015** | **0.041** |
| HDL-4 Free Cholesterol, mg/dL | 98 | 2.9 ± 1.3 | 2.2 ± 0.9 | -0.8 ± 1.1 | **0.003** | **0.011** |
| HDL-4 Phospholipids, mg/dL | 99 | 26.8 ± 4.7 | 25.0 ± 4.2 | -1.8 ± 4.0 | **0.037** | 0.081 |
| HDL-4 Triglycerides, mg/dL | 100 | 4.3 ± 1.0 | 3.3 ± 0.9 | -1.0 ± 0.8 | **<0.001** | **<0.001** |

Apo-A1 = apolipoprotein A1, Apo-A2 = apolipoprotein A2, Apo-B = apolipoprotein B100, VLDL = very-low-density lipoprotein, IDL = intermediate-density lipoprotein, LDL = low-density lipoprotein, HDL = High-density lipoprotein.

**Supplementary Table 4.**  Morning (fasting) lipid main- and subfractions and their contents of lipids and lipoproteins after five days of high-fat diet (Visit 2) and at study completion (Visit 3), according to group, in men with overweight/obesity. Values are observed means ± SD. Estimates (E) with 95% CI and *p* values for the interaction between time and group, from linear mixed models, with *q* values from Benjamini-Hochberg correction. EXam = Morning exercise, EXpm = evening exercise.

|  | **Visit 2** | **Visit 3** | **E** | **95% CI** | ***p* value** | ***q* value** |
| --- | --- | --- | --- | --- | --- | --- |
| **Total Apo-A1, mg/dL** |  |  |  |  |  |  |
| Control | 131 ± 8 | 130 ± 9 |  |  |  |  |
| EXam | 130 ± 7 | 120 ± 10 | -10.2 | -17.7 to -2.8 | **0.008** | **0.042** |
| EXpm | 129 ± 13 | 120 ± 12 | -9.2 | -16.7 to -1.7 | **0.016** | 0.059 |
| **Total Apo-A2, mg/dL** |  |  |  |  |  |  |
| Control | 30.8 ± 1.5 | 31.7 ± 2.8 |  |  |  |  |
| EXam | 31.0 ± 2.6 | 28.3 ± 2.4 | -3.5 | -5.6 to -1.5 | **0.001** | **0.018** |
| EXpm | 30.5 ± 3.7 | 28.0 ± 3.8 | -3.6 | -5.6 to -1.6 | **0.001** | **0.011** |
| **Total Apo-B100, mg/dL** |  |  |  |  |  |  |
| Control | 102 ± 21 | 98 ± 22 |  |  |  |  |
| EXam | 95 ± 17 | 85 ± 19 | -7.5 | -15.8 to 0.8 | 0.070 | 0.168 |
| EXpm | 80 ± 12 | 72 ± 9 | -6.5 | -14.7 to 1.8 | 0.117 | 0.249 |
| **Total Cholesterol, mg/dL** |  |  |  |  |  |  |
| Control | 211 ± 34 | 210 ± 36 |  |  |  |  |
| EXam | 203 ± 27 | 177 ± 36 | -27.3 | -45.4 to -9.1 | **0.004** | **0.031** |
| EXpm | 182 ± 28 | 156 ± 20 | -30.3 | -48.4 to -12.1 | **0.001** | **0.013** |
| **Total Triglycerides, mg/dL** |  |  |  |  |  |  |
| Control | 136 ± 53 | 128 ± 64 |  |  |  |  |
| EXam | 133 ± 71 | 85 ± 23 | -40.4 | -73.8 to -6.9 | **0.018** | 0.069 |
| EXpm | 79 ± 9 | 63 ± 10 | -22.4 | -55.8 to 11.1 | 0.178 | 0.338 |
| **VLDL Apo-B100, mg/dL** |  |  |  |  |  |  |
| Control | 9.8 ± 3.4 | 8.6 ± 3.2 |  |  |  |  |
| EXam | 8.5 ± 3.5 | 6.9 ± 1.7 | -0.6 | -2.3 to 1.0 | 0.432 | 0.547 |
| EXpm | 5.8 ± 0.7 | 5.0 ± 0.8 | -0.3 | -2.0 to 1.4 | 0.723 | 0.820 |
| **VLDL Cholesterol, mg/dL** |  |  |  |  |  |  |
| Control | 22.5 ± 9.7 | 19.0 ± 9.3 |  |  |  |  |
| EXam | 19.4 ± 11.4 | 12.9 ± 4.4 | -3.9 | -9.7 to 1.9 | 0.173 | 0.269 |
| EXpm | 11.9 ± 3.1 | 8.0 ± 2.6 | -3.4 | -9.1 to 2.4 | 0.243 | 0.382 |
| **VLDL Free Cholesterol, mg/dL** |  |  |  |  |  |  |
| Control | 11.2 ± 4.5 | 9.8 ± 4.6 |  |  |  |  |
| EXam | 9.7 ± 4.8 | 6.5 ± 2.1 | -2.1 | -4.5 to 0.4 | 0.088 | 0.177 |
| EXpm | 6.2 ± 1.1 | 4.6 ± 1.0 | -1.4 | -3.8 to 1.1 | 0.252 | 0.382 |
| **VLDL Phospholipids, mg/dL** |  |  |  |  |  |  |
| Control | 25.9 ± 9.9 | 23.1 ± 10.5 |  |  |  |  |
| EXam | 22.9 ± 11.5 | 15.5 ± 4.8 | -5.3 | -10.8 to 0.2 | 0.056 | 0.151 |
| EXpm | 14.7 ± 2.5 | 10.6 ± 2.6 | -3.8 | -9.3 to 1.7 | 0.164 | 0.328 |
| **VLDL Triglycerides, mg/dL** |  |  |  |  |  |  |
| Control | 96.8 ± 44.8 | 89.9 ± 52.8 |  |  |  |  |
| EXam | 92.7 ± 53.3 | 55.0 ± 17.7 | -31.7 | -56.4 to -6.9 | **0.012** | 0.055 |
| EXpm | 51.5 ± 5.6 | 38 ± 7.5 | -16.3 | -41.0 to 8.5 | 0.186 | 0.344 |
| **VLDL-1 Cholesterol, mg/dL** |  |  |  |  |  |  |
| Control | 8.1 ± 4.3 | 7.5 ± 5.8 |  |  |  |  |
| EXam | 7.8 ± 6.3 | 3.9 ± 1.8 | -3.5 | -6.5 to -0.4 | **0.024** | 0.087 |
| EXpm | 3.9 ± 1.0 | 2.3 ± 0.6 | -2.4 | -5.5 to 0.6 | 0.112 | 0.248 |
| **VLDL-1 Free cholesterol, mg/dL** |  |  |  |  |  |  |
| Control | 3.4 ± 1.8 | 3.3 ±2.3 |  |  |  |  |
| EXam | 3.5 ± 2.5 | 1.7 ± 0.8 | -1.7 | -2.8 to -0.5 | **0.005** | **0.034** |
| EXpm | 1.6 ± 0.4 | 0.8 ± 0.4 | -1.1 | -2.3 to 0.04 | 0.055 | 0.150 |
| **VLDL-1 Phospholipids, mg/dL** |  |  |  |  |  |  |
| Control | 8.2 ± 4.1 | 7.9 ± 5.2 |  |  |  |  |
| EXam | 8.3 ± 5.7 | 4.4 ± 2.0 | -3.5 | -6.0 to -1.1 | **0.005** | **0.034** |
| EXpm | 4.4 ± 0.6 | 2.9 ± 0.7 | -2.0 | -4.5 to 0.4 | 0.098 | 0.223 |
| **VLDL-1 Triglycerides, mg/dL** |  |  |  |  |  |  |
| Control | 52.7 ± 27.3 | 52.7 ± 37.6 |  |  |  |  |
| EXam | 53.2 ± 35.1 | 28.6 ± 11.1 | -24.5 | -40.8 to -8.2 | **0.004** | **0.031** |
| EXpm | 28.2 ± 4.1 | 20.2 ± 5.0 | -13.6 | -30.0 to 2.7 | 0.095 | 0.220 |
| **VLDL-2 Cholesterol, mg/dL** |  |  |  |  |  |  |
| Control | 3.1 ± 1.8 | 2.4 ± 1.6 |  |  |  |  |
| EXam | 2.9 ± 2.3 | 1.4 ± 0.7 | -0.9 | -2.0 to 0.2 | 0.104 | 0.198 |
| EXpm | 1.3 ± 0.5 | 0.5 ± 0.4 | -0.6 | -1.7 to 0.5 | 0.250 | 0.382 |
| **VLDL-2 Free cholesterol, mg/dL** |  |  |  |  |  |  |
| Control | 1.3 ± 0.8 | 1.1 ± 0.8 |  |  |  |  |
| EXam | 1.2 ± 0.9 | 0.6 ± 0.3 | -0.4 | -0.9 to 0.04 | 0.069 | 0.168 |
| EXpm | 0.6 ± 0.2 | 0.2 ± 0.2 | -0.3 | -0.7 to 0.1 | 0.178 | 0.338 |
| **VLDL-2 Phospholipids, mg/dL** |  |  |  |  |  |  |
| Control | 3.7 ± 1.9 | 3.1 ± 1.7 |  |  |  |  |
| EXam | 3.6 ± 2.2 | 1.9 ± 0.9 | -1.1 | -2.1 to -0.1 | **0.028** | 0.098 |
| EXpm | 1.9 ± 0.4 | 1.1 ± 0.4 | -0.7 | -1.7 to 0.3 | 0.179 | 0.338 |
| **VLDL-2 Triglycerides, mg/dL** |  |  |  |  |  |  |
| Control | 14.0 ± 7.8 | 11.7 ± 6.8 |  |  |  |  |
| EXam | 13.6 ± 8.9 | 6.6 ± 3.3 | -4.8 | -8.8 to -0.7 | **0.020** | 0.074 |
| EXpm | 6.9 ± 2.0 | 3.6 ± 1.9 | -2.6 | -6.6 to 1.5 | 0.201 | 0.353 |
| **VLDL-3 Cholesterol, mg/dL** |  |  |  |  |  |  |
| Control | 3.7 ± 1.9 | 2.9 ± 1.8 |  |  |  |  |
| EXam | 3.0 ± 2.2 | 1.8 ± 0.9 | -0.6 | -1.7 to 0.5 | 0.243 | 0.352 |
| EXpm | 1.6 ± 0.5 | 0.8 ± 0.7 | -0.6 | -1.7 to 0.5 | 0.246 | 0.382 |
| **VLDL-3 Free cholesterol, mg/dL** |  |  |  |  |  |  |
| Control | 1.7 ± 1.0 | 1.4 ± 1.0 |  |  |  |  |
| EXam | 1.4 ± 1.1 | 0.8 ± 0.4 | -0.4 | -0.9 to 0.2 | 0.153 | 0.251 |
| EXpm | 0.7 ± 0.2 | 0.4 ± 0.2 | -0.3 | -0.8 to 0.3 | 0.327 | 0.464 |
| **VLDL-3 Phospholipids, mg/dL** |  |  |  |  |  |  |
| Control | 4.2 ± 1.9 | 3.6 ± 1.8 |  |  |  |  |
| EXam | 3.6 ± 1.9 | 2.5 ± 0.9 | -0.7 | -1.6 to 0.3 | 0.146 | 0.250 |
| EXpm | 2.2 ± 0.4 | 1.4 ± 0.6 | -0.5 | -1.4 to 0.4 | 0.263 | 0.392 |
| **VLDL-3 Triglycerides, mg/dL** |  |  |  |  |  |  |
| Control | 11.5 ± 6.2 | 9.3 ± 5.3 |  |  |  |  |
| EXam | 10.2 ± 6.2 | 5.7 ± 3.6 | -2.6 | -5.6 to 0.5 | 0.089 | 0.177 |
| EXpm | 5.4 ± 1.4 | 3.1 ± 2.0 | -1.4 | -4.5 to 1.6 | 0.347 | 0.469 |
| **VLDL-4 Cholesterol, mg/dL** |  |  |  |  |  |  |
| Control | 5.8 ± 2.0 | 4.8 ± 1.4 |  |  |  |  |
| EXam | 4.5 ± 2.0 | 4.0 ± 1.4 | 0.2 | -1.0 to 1.4 | 0.751 | 0.799 |
| EXpm | 3.1 ± 0.9 | 2.6 ± 1.1 | -0.1 | -1.3 to 1.0 | 0.803 | 0.840 |
| **VLDL-4 Free cholesterol, mg/dL** |  |  |  |  |  |  |
| Control | 2.2 ± 0.9 | 2.0 ± 0.6 |  |  |  |  |
| EXam | 1.8 ± 0.8 | 1.7 ± 0.6 | 0.1 | -0.4 to 0.6 | 0.687 | 0.772 |
| EXpm | 1.3 ± 0.5 | 1.1 ± 0.5 | -0.2 | -0.7 to 0.4 | 0.509 | 0.644 |
| **VLDL-4 Phospholipids, mg/dL** |  |  |  |  |  |  |
| Control | 5.3 ± 1.6 | 4.5 ± 1.1 |  |  |  |  |
| EXam | 4.4 ± 1.4 | 4.0 ± 1.1 | 0.3 | -0.6 to 1.1 | 0.483 | 0.582 |
| EXpm | 3.2 ± 0.6 | 2.9 ± 0.8 | 0.1 | -0.8 to 0.9 | 0.817 | 0.842 |
| **VLDL-4 Triglycerides, mg/dL** |  |  |  |  |  |  |
| Control | 10.2 ± 3.6 | 8.5 ± 2.8 |  |  |  |  |
| EXam | 8.0 ± 3.0 | 7.0 ± 2.3 | 0.2 | -1.5 to 2.0 | 0.774 | 0.815 |
| EXpm | 5.7 ± 0.8 | 5.2 ± 1.6 | 0.4 | -1.4 to 2.1 | 0.662 | 0.786 |
| **VLDL-5 Cholesterol, mg/dL** |  |  |  |  |  |  |
| Control | 1.6 ± 0.5 | 1.2 ± 0.4 |  |  |  |  |
| EXam | 1.7 ± 0.5 | 1.3 ± 0.5 | 0.4 | 0.01 to 0.7 | **0.041** | 0.125 |
| EXpm | 1.0 ± 0.3 | 1.1 ± 0.3 | 0.2 | -0.2 to 0.5 | 0.297 | 0.431 |
| **VLDL-5 Free cholesterol, mg/dL** |  |  |  |  |  |  |
| Control | 0.8 ± 0.3 | 0.7 ± 0.4 |  |  |  |  |
| EXam | 0.6 ± 0.4 | 0.5 ± 0.3 | -0.1 | -0.4 to 0.2 | 0.374 | 0.499 |
| EXpm | 0.5 ± 0.2 | 0.4 ± 0.2 | -0.2 | -0.5 to 0.1 | 0.916 | 0.351 |
| **VLDL-5 Phospholipids, mg/dL** |  |  |  |  |  |  |
| Control | 2.0 ± 0.6 | 1.6 ± 0.5 |  |  |  |  |
| EXam | 1.5 ± 0.4 | 1.5 ± 0.5 | 0.3 | -0.1 to 0.6 | 0.175 | 0.269 |
| EXpm | 1.4 ± 0.4 | 1.3 ± 0.3 | 0.1 | -0.3 to 0.4 | 0.760 | 0.826 |
| **VLDL-5 Triglycerides, mg/dL** |  |  |  |  |  |  |
| Control | 3.0 ± 0.7 | 2.6 ± 0.5 |  |  |  |  |
| EXam | 2.5 ± 0.5 | 2.7 ± 0.5 | 0.4 | -0.1 to 0.8 | 0.105 | 0.198 |
| EXpm | 2.4 ± 0.4 | 2.5 ± 0.3 | 0.2 | -0.2 to 0.7 | 0.293 | 0.431 |
| **IDL Apo-B100, mg/dL** |  |  |  |  |  |  |
| Control | 5.9 ± 1.0 | 5.9 ± 1.4 |  |  |  |  |
| EXam | 5.5 ± 1.6 | 5.1 ± 1.5 | -0.5 | -1.4 to 0.4 | 0.260 | 0.371 |
| EXpm | 4.1 ± 0.9 | 3.8 ± 1.4 | -0.7 | -1.6 to 0.2 | 0.115 | 0.249 |
| **IDL Cholesterol, mg/dL** |  |  |  |  |  |  |
| Control | 14.3 ± 3.7 | 14.1 ± 4.6 |  |  |  |  |
| EXam | 12.7 ± 4.4 | 10.9 ± 4.0 | -2.0 | -4.6 to 0.7 | 0.133 | 0.243 |
| EXpm | 8.9 ± 2.5 | 7.4 ± 2.2 | -2.5 | -5.1 to 0.1 | 0.058 | 0.152 |
| **IDL Free Cholesterol, mg/dL** |  |  |  |  |  |  |
| Control | 4.1 ± 1.1 | 4.0 ± 1.4 |  |  |  |  |
| EXam | 3.7 ± 1.4 | 3.1 ± 1.2 | -0.7 | -1.5 to 0.1 | 0.074 | 0.168 |
| EXpm | 2.4 ± 0.8 | 2.0 ± 0.6 | -0.8 | -1.6 to -0.01 | **0.043** | 0.131 |
| **IDL Phospholipids, mg/dL** |  |  |  |  |  |  |
| Control | 8.4 ± 3.3 | 8.0 ± 3.8 |  |  |  |  |
| EXam | 8.0 ± 3.3 | 5.2 ± 2.1 | -2.5 | -4.2 to -0.8 | **0.004** | **0.032** |
| EXpm | 4.7 ± 1.3 | 3.1 ± 0.9 | -1.8 | -3.5 to -0.1 | **0.031** | 0.105 |
| **IDL Triglycerides, mg/dL** |  |  |  |  |  |  |
| Control | 13.7 ± 8.7 | 12.3 ± 9.9 |  |  |  |  |
| EXam | 13.6 ± 11.6 | 5.2 ± 3.0 | -6.9 | -12.4 to -1.5 | **0.013** | 0.056 |
| EXpm | 4.3 ± 1.5 | 1.5 ± 1.8 | -4.0 | -9.5 to 1.5 | 0.144 | 0.300 |
| **LDL Apo-B100, mg/dL** |  |  |  |  |  |  |
| Control | 83 ± 19.4 | 81 ± 19.6 |  |  |  |  |
| EXam | 79 ± 15.7 | 71 ± 16.0 | -7.1 | -15.0 to 0.8 | 0.073 | 0.169 |
| EXpm | 67 ± 11.0 | 60 ± 9.1 | -7.3 | -15.2 to 0.6 | 0.065 | 0.163 |
| **LDL Cholesterol, mg/dL** |  |  |  |  |  |  |
| Control | 130 ± 31 | 131 ± 32 |  |  |  |  |
| EXam | 124 ± 27 | 112 ± 27 | -14 | -29.8 to 2.3 | 0.088 | 0.177 |
| EXpm | 113 ± 20 | 97 ± 18 | -20 | -36.0 to -3.9 | **0.015** | 0.056 |
| **LDL Free Cholesterol, mg/dL** |  |  |  |  |  |  |
| Control | 36.7 ± 7.7 | 37.7 ± 8.1 |  |  |  |  |
| EXam | 35.8 ± 7.5 | 32.8 ± 7.0 | -4.2 | -8.5 to 0.1 | 0.054 | 0.150 |
| EXpm | 33.6 ± 5.4 | 29.6 ± 4.4 | -5.8 | -10.1 to -1.4 | **0.009** | **0.044** |
| **LDL Phospholipids, mg/dL** |  |  |  |  |  |  |
| Control | 70.3 ± 13.9 | 70.8 ± 14.2 |  |  |  |  |
| EXam | 67.9 ± 13.0 | 61.9 ± 13.7 | -6.9 | -13.9 to 0.1 | 0.050 | 0.148 |
| EXpm | 61.9 ± 9.9 | 54.3 ± 8.9 | -9.5 | -16.5 to -2.5 | **0.008** | **0.043** |
| **LDL Triglycerides, mg/dL** |  |  |  |  |  |  |
| Control | 18.6 ± 3.0 | 17.3 ± 3.5 |  |  |  |  |
| EXam | 18.7 ± 5.3 | 17.8 ± 4.5 | 0.4 | -1.2 to 2.1 | 0.661 | 0.752 |
| EXpm | 14.1 ± 2.5 | 14.9 ± 2.7 | 1.7 | -0.02 to 2.4 | **0.049** | 0.139 |
| **LDL-1 Apo-B100, mg/dL** |  |  |  |  |  |  |
| Control | 11.8 ± 1.8 | 12.9 ± 2.3 |  |  |  |  |
| EXam | 12.0 ± 1.9 | 11.3 ± 2.1 | -1.8 | -3.0 to -0.5 | **0.005** | **0.034** |
| EXpm | 11.2 ± 2.7 | 10.0 ± 2.1 | -2.5 | -3.7 to -1.2 | **<0.001** | **0.006** |
| **LDL-1 Cholesterol, mg/dL** |  |  |  |  |  |  |
| Control | 22.6 ± 2.8 | 24.9 ± 4.0 |  |  |  |  |
| EXam | 23.1 ± 4.4 | 21.0 ± 4.5 | -4.3 | -6.9 to -1.7 | **0.002** | **0.020** |
| EXpm | 21.7 ± 5.9 | 18.1 ± 4.8 | -6.1 | -8.7 to -3.5 | **<0.001** | **0.002** |
| **LDL-1 Free cholesterol, mg/dL** |  |  |  |  |  |  |
| Control | 6.7 ± 0.8 | 7.2 ± 1.1 |  |  |  |  |
| EXam | 6.8 ± 1.2 | 6.3 ± 1.2 | -1.0 | -1.8 to -0.2 | **0.011** | 0.051 |
| EXpm | 6.4 ± 1.6 | 5.6 ± 1.1 | -1.5 | -2.3 to -0.7 | **<0.001** | **0.006** |
| **LDL-1 Phospholipids, mg/dL** |  |  |  |  |  |  |
| Control | 12.7 ± 1.7 | 13.8 ± 2.1 |  |  |  |  |
| EXam | 13.0 ± 2.0 | 12.1 ± 2.3 | -1.9 | -3.1 to -0.7 | **0.003** | **0.031** |
| EXpm | 12.3 ± 3.0 | 10.6 ± 2.5 | -2.8 | -4.1 to -1.6 | **<0.001** | **0.002** |
| **LDL-1 Triglycerides, mg/dL** |  |  |  |  |  |  |
| Control | 5.1 ± 1.7 | 5.2 ± 1.6 |  |  |  |  |
| EXam | 5.2 ± 1.8 | 5.1 ± 1.5 | -0.3 | -1.0 to 0.5 | 0.455 | 0.569 |
| EXpm | 3.9 ± 1.1 | 4.3 ± 1.1 | 0.1 | -0.6 to 0.8 | 0.780 | 0.830 |
| **LDL-2 Apo-B100, mg/dL** |  |  |  |  |  |  |
| Control | 9.7 ± 2.2 | 10.8 ± 3.1 |  |  |  |  |
| EXam | 9.9 ± 4.1 | 10.8 ± 1.3 | -0.1 | -2.4 to 2.1 | 0.903 | 0.917 |
| EXpm | 11.0 ± 1.8 | 10.8 ± 1.6 | -0.5 | -2.7 to 1.8 | 0.691 | 0.803 |
| **LDL-2 Cholesterol, mg/dL** |  |  |  |  |  |  |
| Control | 17.2 ± 4.8 | 19.7 ± 6.0 |  |  |  |  |
| EXam | 17.6 ± 8.6 | 19.0 ± 2.6 | -0.9 | -5.5 to 3.8 | 0.712 | 0.778 |
| EXpm | 20.1 ± 3.5 | 19.4 ± 3.3 | -1.4 | -6.0 to 3.2 | 0.550 | 0.687 |
| **LDL-2 Free cholesterol, mg/dL** |  |  |  |  |  |  |
| Control | 5.2 ± 1.2 | 5.9 ± 1.8 |  |  |  |  |
| EXam | 5.4 ± 2.5 | 5.9 ± 0.6 | -0.1 | -1.5 to 1.3 | 0.908 | 0.917 |
| EXpm | 6.2 ± 1.1 | 6.2 ± 1.1 | 0.0 | -1.4 to 1.4 | 0.961 | 0.961 |
| **LDL-2 Phospholipids, mg/dL** |  |  |  |  |  |  |
| Control | 9.3 ± 2.2 | 10.6 ± 3.0 |  |  |  |  |
| EXam | 9.7 ± 4.1 | 10.4 ± 1.3 | -0.4 | -2.6 to 1.9 | 0.749 | 0.799 |
| EXpm | 10.9 ± 1.7 | 10.6 ± 1.6 | -0.6 | -2.9 to 1.6 | 0.579 | 0.706 |
| **LDL-2 Triglycerides, mg/dL** |  |  |  |  |  |  |
| Control | 1.9 ± 0.6 | 2.1 ± 0.6 |  |  |  |  |
| EXam | 2.1 ± 0.5 | 2.2 ± 0.5 | -0.1 | -0.3 to 0.1 | 0.383 | 0.504 |
| EXpm | 1.9 ± 0.4 | 1.9 ± 0.5 | -0.3 | -0.5 to -0.03 | **0.024** | 0.085 |
| **LDL-3 Apo-B100, mg/dL** |  |  |  |  |  |  |
| Control | 9.4 ± 3.5 | 11.1 ± 3.8 |  |  |  |  |
| EXam | 9.6 ± 4.0 | 9.6 ± 3.0 | -1.6 | -3.9 to 0.7 | 0.158 | 0.255 |
| EXpm | 10.6 ± 2.4 | 8.9 ± 2.3 | -2.9 | -5.2 to -0.7 | **0.012** | **0.0498** |
| **LDL-3 Cholesterol, mg/dL** |  |  |  |  |  |  |
| Control | 15.7 ± 6.7 | 19.0 ± 7.3 |  |  |  |  |
| EXam | 16.0 ± 8.0 | 15.8 ± 5.9 | -3.4 | -7.9 to 1.0 | 0.123 | 0.228 |
| EXpm | 18.3 ± 4.7 | 14.9 ± 4.5 | -5.9 | -10.4 to -1.4 | **0.010** | **0.044** |
| **LDL-3 Free cholesterol, mg/dL** |  |  |  |  |  |  |
| Control | 4.7 ± 1.5 | 5.4 ± 1.8 |  |  |  |  |
| EXam | 5.0 ± 2.0 | 4.8 ± 1.4 | -0.8 | -2.0 to 0.3 | 0.149 | 0.252 |
| EXpm | 5.5 ± 1.2 | 4.8 ± 1.2 | -1.2 | -2.3 to -0.04 | **0.041** | 0.130 |
| **LDL-3 Phospholipids, mg/dL** |  |  |  |  |  |  |
| Control | 8.7 ± 3.3 | 10.3 ± 3.6 |  |  |  |  |
| EXam | 8.9 ± 3.8 | 8.8 ± 2.9 | -1.6 | -3.7 to 0.6 | 0.142 | 0.252 |
| EXpm | 10.0 ± 2.3 | 8.4 ± 2.3 | -2.9 | -5.0 to -0.7 | **0.009** | **0.044** |
| **LDL-3 Triglycerides, mg/dL** |  |  |  |  |  |  |
| Control | 2.6 ± 0.3 | 2.5 ± 0.3 |  |  |  |  |
| EXam | 2.5 ± 0.5 | 2.5 ± 0.6 | 0.1 | -0.2 to 0.3 | 0.577 | 0.677 |
| EXpm | 2.2 ± 0.4 | 2.2 ± 0.4 | 0.0 | -0.2 to 0.2 | 0.731 | 0.820 |
| **LDL-4 Apo-B100, mg/dL** |  |  |  |  |  |  |
| Control | 12.4 ± 4.4 | 12.6 ± 4.3 |  |  |  |  |
| EXam | 10.9 ± 4.8 | 10.4 ± 4.8 | -0.9 | -2.9 to 1.2 | 0.392 | 0.510 |
| EXpm | 10.6 ± 3.5 | 7.9 ± 3.4 | -3.1 | -5.2 to -1.1 | **0.003** | **0.020** |
| **LDL-4 Cholesterol, mg/dL** |  |  |  |  |  |  |
| Control | 19.9 ± 7.6 | 20.4 ± 7.4 |  |  |  |  |
| EXam | 17.4 ± 8.2 | 16.3 ± 8.0 | -2.0 | -5.6 to 1.7 | 0.272 | 0.383 |
| EXpm | 17.5 ± 5.8 | 12.5 ± 5.9 | -5.9 | -9.6 to -2.3 | **0.002** | **0.013** |
| **LDL-4 Free cholesterol, mg/dL** |  |  |  |  |  |  |
| Control | 5.5 ± 1.8 | 5.7 ± 1.8 |  |  |  |  |
| EXam | 5.1 ± 1.9 | 4.8 ± 1.8 | -0.5 | -1.5 to 0.5 | 0.303 | 0.421 |
| EXpm | 5.2 ± 1.3 | 4.1 ± 1.4 | -1.3 | -2.2 to -0.3 | **0.013** | 0.050 |
| **LDL-4 Phospholipids, mg/dL** |  |  |  |  |  |  |
| Control | 10.7 ± 3.8 | 11.0 ± 3.6 |  |  |  |  |
| EXam | 9.5 ± 4.1 | 8.9 ± 4.1 | -1.0 | -2.8 to 0.7 | 0.231 | 0.345 |
| EXpm | 9.5 ± 3.0 | 7.0 ± 3.0 | -3.0 | -4.7 to -1.2 | **0.001** | **0.013** |
| **LDL-4 Triglycerides, mg/dL** |  |  |  |  |  |  |
| Control | 2.4 ± 0.6 | 2.0 ± 0.8 |  |  |  |  |
| EXam | 2.3 ± 1.2 | 2.4 ± 0.9 | 0.4 | 0.1 to 0.8 | **0.018** | 0.069 |
| EXpm | 1.8 ± 0.7 | 1.7 ± 0.7 | 0.2 | -0.1 to 0.6 | 0.213 | 0.357 |
| **LDL-5 Apo-B100, mg/dL** |  |  |  |  |  |  |
| Control | 17.7 ± 5.8 | 15.0 ± 5.5 |  |  |  |  |
| EXam | 15.3 ± 4.0 | 13.5 ± 3.9 | 0.7 | -1.2 to 2.6 | 0.475 | 0.579 |
| EXpm | 11.5 ± 1.9 | 10.2 ± 2.0 | 0.9 | -1.0 to 2.8 | 0.355 | 0.473 |
| **LDL-5 Cholesterol, mg/dL** |  |  |  |  |  |  |
| Control | 25.6 ± 8.8 | 21.9 ± 8.3 |  |  |  |  |
| EXam | 21.9 ± 5.9 | 19.3 ± 5.9 | 0.7 | -2.2 to 3.6 | 0.607 | 0.698 |
| EXpm | 17.0 ± 3 | 14.5 ± 3.1 | 0.5 | -2.4 to 3.4 | 0.750 | 0.824 |
| **LDL-5 Free cholesterol, mg/dL** |  |  |  |  |  |  |
| Control | 6.7 ± 2.0 | 5.9 ± 1.9 |  |  |  |  |
| EXam | 5.9 ± 1.5 | 5.3 ± 1.5 | 0.1 | -0.6 to 0.9 | 0.716 | 0.778 |
| EXpm | 4.8 ± 0.7 | 4.3 ± 0.9 | 0.1 | -0.6 to 0.9 | 0.738 | 0.820 |
| **LDL-5 Phospholipids, mg/dL** |  |  |  |  |  |  |
| Control | 13.4 ± 4.2 | 11.5 ± 3.9 |  |  |  |  |
| EXam | 11.7 ± 3.0 | 10.4 ± 2.9 | 0.5 | -0.9 to 1.8 | 0.471 | 0.579 |
| EXpm | 9.2 ± 1.4 | 8.0 ± 1.6 | 0.4 | -1.0 to 1.7 | 0.571 | 0.705 |
| **LDL-5 Triglycerides, mg/dL** |  |  |  |  |  |  |
| Control | 3.0 ± 0.9 | 2.3 ± 1.0 |  |  |  |  |
| EXam | 2.8 ± 1.3 | 2.4 ± 0.8 | 0.3 | -0.2 to 0.8 | 0.200 | 0.303 |
| EXpm | 1.7 ± 0.6 | 1.6 ± 0.5 | 0.5 | 0.01 to 1.0 | **0.042** | 0.130 |
| **LDL-6 Apo-B100, mg/dL** |  |  |  |  |  |  |
| Control | 23.2 ± 10.4 | 20.0 ± 11 |  |  |  |  |
| EXam | 22.5 ± 6.8 | 16.6 ± 4 | -2.9 | -6.4 to 0.7 | 0.101 | 0.198 |
| EXpm | 14.0 ± 2.3 | 13.9 ± 2 | 2.1 | -1.4 to 5.7 | 0.223 | 0.356 |
| **LDL-6 Cholesterol, mg/dL** |  |  |  |  |  |  |
| Control | 29.0 ± 12.8 | 25.2 ± 13 |  |  |  |  |
| EXam | 28.0 ± 7.5 | 20.7 ± 5 | -3.7 | -7.8 to 0.3 | 0.067 | 0.167 |
| EXpm | 18.1 ± 2.9 | 17.4 ± 3 | 1.9 | -2.1 to 6.0 | 0.333 | 0.464 |
| **LDL-6 Free cholesterol, mg/dL** |  |  |  |  |  |  |
| Control | 7.0 ± 2.8 | 6.2 ± 2.7 |  |  |  |  |
| EXam | 7.0 ± 1.5 | 5.3 ± 1.0 | -0.9 | -1.8 to -0.02 | **0.040** | 0.124 |
| EXpm | 4.8 ± 0.9 | 4.8 ± 0.7 | 0.6 | -0.3 to 1.5 | 0.207 | 0.356 |
| **LDL-6 Phospholipids, mg/dL** |  |  |  |  |  |  |
| Control | 15.3 ± 6.1 | 13.5 ± 6.4 |  |  |  |  |
| EXam | 15.0 ± 3.9 | 11.4 ± 2.3 | -1.9 | -4.0 to 0.2 | 0.067 | 0.167 |
| EXpm | 10.1 ± 1.3 | 9.8 ± 1.3 | 1.0 | -1.1 to 3.0 | 0.334 | 0.464 |
| **LDL-6 Triglycerides, mg/dL** |  |  |  |  |  |  |
| Control | 4.4 ± 1.8 | 3.9 ± 1.8 |  |  |  |  |
| EXam | 4.4 ± 1.6 | 3.4 ± 0.9 | -0.4 | -1.1 to 0.2 | 0.160 | 0.255 |
| EXpm | 3.0 ± 0.6 | 3.1 ± 0.7 | 0.6 | -0.1 to 1.2 | 0.084 | 0.201 |
| **HDL Apo-A1, mg/dL** |  |  |  |  |  |  |
| Control | 131 ± 8 | 130 ± 10 |  |  |  |  |
| EXam | 130 ± 8 | 121 ± 10 | -8.7 | -17.0 to -0.5 | **0.038** | 0.122 |
| EXpm | 130 ± 13 | 122 ± 14 | -7.3 | -15.5 to 1.0 | 0.081 | 0.198 |
| **HDL Apo-A2, mg/dL** |  |  |  |  |  |  |
| Control | 31.5 ± 1.5 | 32.3 ± 2.8 |  |  |  |  |
| EXam | 31.5 ± 2.6 | 29.0 ± 2.4 | -3.3 | -5.2 to -1.3 | **0.001** | **0.018** |
| EXpm | 30.8 ± 3.5 | 28.5 ± 3.5 | -3.2 | -5.2 to -1.3 | **0.001** | **0.013** |
| **Total HDL Cholesterol, mg/dL** |  |  |  |  |  |  |
| Control | 48.2 ± 4.0 | 48.8 ± 5.8 |  |  |  |  |
| EXam | 48.5 ± 6.3 | 45.9 ± 4.5 | -3.1 | -7.3 to 1.2 | 0.146 | 0.250 |
| EXpm | 50.1 ± 6.5 | 48.0 ± 5.6 | -2.0 | -6.3 to 2.2 | 0.342 | 0.469 |
| **HDL Free Cholesterol, mg/dL** |  |  |  |  |  |  |
| Control | 9.7 ± 1.0 | 9.8 ± 1.3 |  |  |  |  |
| EXam | 10.5 ± 1.1 | 9.2 ± 1.2 | -1.2 | -2.3 to -0.2 | **0.017** | 0.069 |
| EXpm | 11.2 ± 2.1 | 10.2 ± 1.6 | -0.7 | -1.7 to 0.3 | 0.155 | 0.137 |
| **HDL Phospholipids, mg/dL** |  |  |  |  |  |  |
| Control | 59.2 ± 6.7 | 60.2 ± 9.3 |  |  |  |  |
| EXam | 61.9 ± 5.5 | 57.6 ± 6.0 | -4.4 | -9.4 to 0.6 | 0.083 | 0.177 |
| EXpm | 63.1 ± 8.2 | 59.8 ± 7.5 | -3.1 | -8.1 to 2.0 | 0.221 | 0.359 |
| **HDL Triglycerides, mg/dL** |  |  |  |  |  |  |
| Control | 8.6 ± 1.7 | 7.8 ± 1.4 |  |  |  |  |
| EXam | 8.5 ± 2.9 | 7.3 ± 1.5 | -0.4 | -1.8 to 1.0 | 0.582 | 0.677 |
| EXpm | 6.9 ± 1.5 | 6.1 ± 1.5 | -0.5 | -1.9 to 0.9 | 0.451 | 0.586 |
| **HDL-1 Apo-A1, mg/dL** |  |  |  |  |  |  |
| Control | 11.7 ± 3.3 | 11.3 ± 3 |  |  |  |  |
| EXam | 14.5 ± 6.1 | 15.1 ± 6 | 1.5 | -1.1 to 4.2 | 0.240 | 0.352 |
| EXpm | 11.7 ± 3.3 | 17.3 ± 3 | 3.0 | 0.3 to 5.6 | **0.027** | 0.092 |
| **HDL-1 Apo-A2, mg/dL** |  |  |  |  |  |  |
| Control | 0.7 ± 0.2 | 0.9 ± 0.4 |  |  |  |  |
| EXam | 1.1 ± 0.7 | 1.1 ± 0.5 | -0.1 | -0.4 to 0.2 | 0.542 | 0.645 |
| EXpm | 1.2 ± 0.4 | 1.2 ± 0.4 | 0.01 | -0.3 to 0.3 | 0.957 | 0.961 |
| **HDL-1 Cholesterol, mg/dL** |  |  |  |  |  |  |
| Control | 9.4 ± 2.4 | 10.3 ± 3.3 |  |  |  |  |
| EXam | 11.1 ± 3.5 | 10.2 ± 2.6 | -1.3 | -3.2 to 0.5 | 0.150 | 0.250 |
| EXpm | 11.3 ± 2.3 | 11.4 ± 2.1 | -0.3 | -2.1 to 1.6 | 0.773 | 0.830 |
| **HDL-1 Free cholesterol, mg/dL** |  |  |  |  |  |  |
| Control | 2.4 ± 0.5 | 2.7 ± 0.7 |  |  |  |  |
| EXam | 3.1 ± 0.7 | 2.7 ± 0.5 | -0.5 | -1.0 to 0.1 | 0.087 | 0.177 |
| EXpm | 3.3 ± 0.7 | 3.1 ± 0.6 | -0.2 | -0.7 to 0.3 | 0.428 | 0.563 |
| **HDL-1 Phospholipids, mg/dL** |  |  |  |  |  |  |
| Control | 9.3 ± 2.1 | 10.2 ± 2.9 |  |  |  |  |
| EXam | 11.8 ± 3.9 | 11.4 ± 3.3 | -0.8 | -2.7 to 1.2 | 0.430 | 0.547 |
| EXpm | 12.6 ± 2.6 | 13.1 ± 2.6 | 0.2 | -1.7 to 2.2 | 0.806 | 0.840 |
| **HDL-1 Triglycerides, mg/dL** |  |  |  |  |  |  |
| Control | 1.9 ± 0.8 | 1.7 ± 0.7 |  |  |  |  |
| EXam | 2.0 ± 0.8 | 1.8 ± 0.7 | 0.0 | -0.5 to 0.4 | 0.966 | 0.966 |
| EXpm | 1.5 ± 0.6 | 1.5 ± 0.6 | 0.1 | -0.4 to 0.5 | 0.668 | 0.786 |
| **HDL-2 Apo-A1, mg/dL** |  |  |  |  |  |  |
| Control | 12.2 ± 2.2 | 13.6 ± 3 |  |  |  |  |
| EXam | 14.2 ± 1.8 | 13.2 ± 2 | -1.2 | -2.4 to 0.03 | 0.053 | 0.150 |
| EXpm | 14.2 ± 1.8 | 13.6 ± 2 | -0.7 | -1.9 to 0.5 | 0.214 | 0.357 |
| **HDL-2 Apo-A2, mg/dL** |  |  |  |  |  |  |
| Control | 2.1 ± 0.4 | 2.4 ± 0.6 |  |  |  |  |
| EXam | 2.4 ± 0.9 | 2.2 ± 0.5 | -0.4 | -0.9 to 0.02 | 0.058 | 0.154 |
| EXpm | 2.4 ± 0.6 | 2.2 ± 0.6 | -0.4 | -0.9 to 0.01 | 0.053 | 0.147 |
| **HDL-2 Cholesterol, mg/dL** |  |  |  |  |  |  |
| Control | 5.5 ± 0.9 | 5.5 ± 1.5 |  |  |  |  |
| EXam | 5.9 ± 1.5 | 5.9 ± 1.2 | 0.1 | -0.7 to 0.9 | 0.817 | 0.851 |
| EXpm | 6.6 ± 0.9 | 6.4 ± 0.9 | 0.1 | -0.7 to 0.9 | 0.728 | 0.820 |
| **HDL-2 Free cholesterol, mg/dL** |  |  |  |  |  |  |
| Control | 1.1 ± 0.4 | 1.3 ± 0.5 |  |  |  |  |
| EXam | 1.3 ± 0.3 | 1.4 ± 0.3 | -0.1 | -0.3 to 0.1 | 0.312 | 0.428 |
| EXpm | 1.6 ± 0.4 | 1.6 ± 0.3 | -0.1 | -0.4 to 0.1 | 0.190 | 0.346 |
| **HDL-2 Phospholipids, mg/dL** |  |  |  |  |  |  |
| Control | 7.9 ± 1.9 | 8.0 ± 2.7 |  |  |  |  |
| EXam | 8.7 ± 2.1 | 8.5 ± 1.6 | -0.1 | -1.2 to 1.0 | 0.845 | 0.872 |
| EXpm | 9.6 ± 1.5 | 9.3 ± 1.4 | 0.0 | -1.2 to 1.1 | 0.936 | 0.955 |
| **HDL-2 Triglycerides, mg/dL** |  |  |  |  |  |  |
| Control | 1.2 ± 0.3 | 1.1 ± 0.3 |  |  |  |  |
| EXam | 1.3 ± 0.5 | 1.2 ± 0.4 | 0.1 | -0.1 to 0.3 | 0.357 | 0.483 |
| EXpm | 1.1 ± 0.3 | 1.1 ± 0.3 | 0.1 | -0.1 to 0.3 | 0.507 | 0.644 |
| **HDL-3 Apo-A1, mg/dL** |  |  |  |  |  |  |
| Control | 20.9 ± 3.8 | 21.6 ± 4.3 |  |  |  |  |
| EXam | 21.5 ± 2.0 | 19.4 ± 2.2 | -2.7 | -4.6 to -0.7 | **0.009** | **0.043** |
| EXpm | 22.3 ± 3.7 | 20.1 ± 3.6 | -2.6 | -4.6 to -0.6 | **0.010** | **0.044** |
| **HDL-3 Apo-A2, mg/dL** |  |  |  |  |  |  |
| Control | 5.0 ± 0.8 | 5.4 ± 0.9 |  |  |  |  |
| EXam | 5.4 ± 0.9 | 4.9 ± 0.7 | -0.8 | -1.3 to -0.3 | **0.001** | **0.018** |
| EXpm | 5.3 ± 1.0 | 4.7 ± 1.0 | -0.9 | -1.4 to -0.4 | **<0.001** | **0.006** |
| **HDL-3 Cholesterol, mg/dL** |  |  |  |  |  |  |
| Control | 7.8 ± 1.5 | 8.2 ± 1.8 |  |  |  |  |
| EXam | 8.2 ± 0.7 | 7.6 ± 0.9 | -0.9 | -1.6 to -0.3 | **0.007** | **0.040** |
| EXpm | 8.7 ± 1.6 | 8.0 ± 1.5 | -1.0 | -1.7 to -0.3 | **0.005** | **0.032** |
| **HDL-3 Free cholesterol, mg/dL** |  |  |  |  |  |  |
| Control | 1.7 ± 0.3 | 1.8 ± 0.5 |  |  |  |  |
| EXam | 1.8 ± 0.3 | 1.4 ± 0.2 | -0.5 | -0.8 to -0.2 | **<0.001** | **0.018** |
| EXpm | 1.9 ± 0.6 | 1.6 ± 0.5 | -0.5 | -0.7 to -0.2 | **0.001** | **0.013** |
| **HDL-3 Phospholipids, mg/dL** |  |  |  |  |  |  |
| Control | 12.0 ± 2.7 | 12.6 ± 3.3 |  |  |  |  |
| EXam | 12.7 ± 1.8 | 11.7 ± 1.5 | -1.4 | -2.8 to -0.1 | **0.036** | 0.121 |
| EXpm | 13.0 ± 2.4 | 12.0 ± 2.4 | -1.4 | -2.7 to 0.01 | **0.048** | 0.139 |
| **HDL-3 Triglycerides, mg/dL** |  |  |  |  |  |  |
| Control | 1.7 ± 0.4 | 1.6 ± 0.3 |  |  |  |  |
| EXam | 1.8 ± 0.7 | 1.5 ± 0.3 | -0.1 | -0.4 to 0.2 | 0.711 | 0.779 |
| EXpm | 1.4 ± 0.3 | 1.3 ± 0.4 | -0.1 | -0.4 to 0.2 | 0.643 | 0.774 |
| **HDL-4 Apo-A1, mg/dL** |  |  |  |  |  |  |
| Control | 85.3 ± 3.9 | 83.9 ± 5.3 |  |  |  |  |
| EXam | 80.0 ± 9.9 | 71.9 ± 9.2 | -7.8 | -12.9 to -2.7 | **0.003** | **0.031** |
| EXpm | 77.6 ± 8.7 | 69.9 ± 9.0 | -7.9 | -13.0 to -2.8 | **0.003** | **0.019** |
| **HDL-4 Apo-A2, mg/dL** |  |  |  |  |  |  |
| Control | 22.7 ± 1.3 | 23.0 ± 2.0 |  |  |  |  |
| EXam | 21.6 ± 2.9 | 19.3 ± 2.5 | -2.8 | -4.3 to -1.4 | **<0.001** | **0.018** |
| EXpm | 20.7 ± 2.8 | 18.6 ± 2.7 | -2.8 | -4.2 to -1.4 | **<0.001** | **0.006** |
| **HDL-4 Cholesterol, mg/dL** |  |  |  |  |  |  |
| Control | 23.1 ± 1.3 | 23.1 ± 1.8 |  |  |  |  |
| EXam | 21.5 ± 3.2 | 19.6 ± 2.7 | -2.3 | -4,0 to -0.6 | **0.007** | **0.040** |
| EXpm | 21.4 ± 3.2 | 19.5 ± 3.0 | -2.4 | -4.1 to -0.7 | **0.006** | **0.034** |
| **HDL-4 Free cholesterol, mg/dL** |  |  |  |  |  |  |
| Control | 4.0 ± 0.3 | 4.1 ± 0.7 |  |  |  |  |
| EXam | 3.8 ± 0.7 | 3.1 ± 0.7 | -0.8 | -1.2 to -0.3 | **0.001** | **0.018** |
| EXpm | 3.8 ± 0.8 | 3.2 ± 0.9 | -0.7 | -1.1 to -0.2 | **0.004** | **0.027** |
| **HDL-4 Phospholipids, mg/dL** |  |  |  |  |  |  |
| Control | 29.2 ± 1.5 | 29.2 ± 2.5 |  |  |  |  |
| EXam | 28.0 ± 3.5 | 25.1 ± 3.8 | -3.2 | -5.1 to -1.3 | **0.001** | **0.018** |
| EXpm | 27.2 ± 3.5 | 24.6 ± 3.6 | -3.1 | -5.0 to -1.2 | **0.002** | **0.013** |
| **HDL-4 Triglycerides, mg/dL** |  |  |  |  |  |  |
| Control | 4.0 ± 0.5 | 3.4 ± 0.5 |  |  |  |  |
| EXam | 3.7 ± 1.2 | 3.0 ± 0.7 | -0.5 | -1.0 to 0.1 | 0.085 | 0.187 |
| EXpm | 3.0 ± 0.4 | 2.4 ± 0.7 | -0.5 | -1.0 to 0.04 | 0.065 | 0.163 |

**Supplementary Table 5.**  Evening (postprandial) lipid main- and subfractions and their contents of lipids and lipoproteins after five days of high-fat diet (Visit 2) and at study completion (Visit 3), according to group, in men with overweight/obesity. Values are observed means ± SD. Estimates (E) with 95% CI and *p* values for the interaction between time and group, from linear mixed models, with *q* values from Benjamini-Hochberg correction. EXam = Morning exercise, EXpm = Evening exercise.

|  | **Visit 2** | **Visit 3** | **E** | **95% CI** | ***p* value** | ***q* value** |
| --- | --- | --- | --- | --- | --- | --- |
| **Total Apo-A1, mg/dL** |  |  |  |  |  |  |
| Control | 140 ± 7 | 136 ± 6 |  |  |  |  |
| EXam | 135 ± 9 | 124 ± 13 | -9.7 | -18.8 to -0.7 | **0.035** | 0.281 |
| EXpm | 134 ± 12 | 126 ± 12 | -7.2 | -16.2 to 1.8 | 0.112 | 0.549 |
| **Total Apo-A2, mg/dL** |  |  |  |  |  |  |
| Control | 35.1 ± 3.0 | 32.6 ± 2.3 |  |  |  |  |
| EXam | 30.1 ± 3.8 | 27.4 ± 3.7 | -2.1 | -5.1 to 1.0 | 0.179 | 0.660 |
| EXpm | 29.9 ± 3.6 | 29.0 ± 3.7 | -0.5 | -3.5 to 2.5 | 0.731 | 0.861 |
| **Total Apo-B100, mg/dL** |  |  |  |  |  |  |
| Control | 100 ± 26 | 94 ± 21 |  |  |  |  |
| EXam | 87 ± 24 | 71 ± 19 | -7.4 | -18.1 to 3.3 | 0.160 | 0.642 |
| EXpm | 68 ± 15 | 67 ± 12 | 2.8 | -7.6 to 13.3 | 0.579 | 0.843 |
| **Total Cholesterol, mg/dL** |  |  |  |  |  |  |
| Control | 229 ± 39 | 217 ± 25 |  |  |  |  |
| EXam | 207 ± 38 | 177 ± 39 | -20.2 | -42.0 to 1.6 | 0.064 | 0.425 |
| EXpm | 180 ± 27 | 160 ± 17 | -16.0 | -37.3 to 5.4 | 0.132 | 0.549 |
| **Total Triglycerides, mg/dL** |  |  |  |  |  |  |
| Control | 272 ± 111 | 198 ± 48 |  |  |  |  |
| EXam | 181 ± 56 | 157 ± 67 | 14.4 | -45.2 to 74.0 | 0.625 | 0.962 |
| EXpm | 163 ± 62 | 110 ± 27 | -14.4 | -73.4 to 44.5 | 0.618 | 0.843 |
| **VLDL Apo-B100, mg/dL** |  |  |  |  |  |  |
| Control | 13.1 ± 4.7 | 10.2 ± 2.5 |  |  |  |  |
| EXam | 9.0 ± 3.1 | 8.0 ± 2.2 | 0.7 | -1.6 to 3.0 | 0.524 | 0.962 |
| EXpm | 7.3 ± 1.8 | 6.1 ± 0.9 | 0.2 | -2.0 to 2.5 | 0.826 | 0.925 |
| **VLDL Cholesterol, mg/dL** |  |  |  |  |  |  |
| Control | 38.0 ± 14.6 | 27.9 ± 7.6 |  |  |  |  |
| EXam | 24.7 ± 8.0 | 21.4 ± 8.8 | 2.1 | -5.3 to 9.5 | 0.562 | 0.962 |
| EXpm | 20.9 ± 6.2 | 13.7 ± 3.7 | -1.8 | -9.1 to 5.5 | 0.624 | 0.843 |
| **VLDL Free Cholesterol, mg/dL** |  |  |  |  |  |  |
| Control | 17.1 ± 6.7 | 12.8 ± 3.3 |  |  |  |  |
| EXam | 11.1 ± 3.8 | 9.4 ± 3.6 | 0.6 | -2.8 to 4.0 | 0.725 | 0.962 |
| EXpm | 9.3 ± 3.1 | 6.6 ± 1.2 | -0.6 | -4.0 to 2.7 | 0.708 | 0.861 |
| **VLDL Phospholipids, mg/dL** |  |  |  |  |  |  |
| Control | 39.8 ± 15.2 | 29.9 ± 8.0 |  |  |  |  |
| EXam | 27.0 ± 9.0 | 23.5 ± 8.8 | 1.6 | -6.3 to 9.6 | 0.676 | 0.962 |
| EXpm | 23.3 ± 7.7 | 15.8 ± 3.4 | -2.6 | -10.5 to 5.2 | 0.498 | 0.829 |
| **VLDL Triglycerides, mg/dL** |  |  |  |  |  |  |
| Control | 190 ± 83 | 137 ± 39 |  |  |  |  |
| EXam | 123 ± 44 | 107 ± 51 | 9.8 | -35 to 55 | 0.659 | 0.962 |
| EXpm | 109 ± 49 | 70 ± 19 | -13.1 | -58 to 31 | 0.551 | 0.843 |
| **VLDL-1 Cholesterol, mg/dL** |  |  |  |  |  |  |
| Control | 20.2 ± 9.2 | 13.2 ± 3.9 |  |  |  |  |
| EXam | 12.0 ± 4.7 | 9.4 ± 5.0 | 1.5 | -3.3 to 6.2 | 0.530 | 0.962 |
| EXpm | 10.6 ± 3.3 | 6.4 ± 2.5 | -0.3 | -5.0 to 4.4 | 0.894 | 0.928 |
| **VLDL-1 Free cholesterol, mg/dL** |  |  |  |  |  |  |
| Control | 8.2 ± 3.4 | 5.9 ± 1.9 |  |  |  |  |
| EXam | 5.6 ± 2.0 | 4.6 ± 2.5 | 0.4 | -1.5 to 2.3 | 0.672 | 0.962 |
| EXpm | 4.9 ± 2.1 | 2.7 ± 1.3 | -0.8 | -2.6 to 1.1 | 0.381 | 0.732 |
| **VLDL-1 Phospholipids, mg/dL** |  |  |  |  |  |  |
| Control | 17.9 ± 8.1 | 12.8 ± 4.0 |  |  |  |  |
| EXam | 11.8 ± 4.7 | 10.1 ± 5.0 | 1.2 | 3.0 to 5.5 | 0.555 | 0.962 |
| EXpm | 10.5 ± 4.5 | 6.5 ± 2.3 | -1.1 | -5.3 to 3.1 | 0.597 | 0.843 |
| **VLDL-1 Triglycerides, mg/dL** |  |  |  |  |  |  |
| Control | 123 ± 57 | 84 ± 25 |  |  |  |  |
| EXam | 77 ± 33 | 64 ± 33 | 7.7 | -23 to 39 | 0.613 | 0.962 |
| EXpm | 69 ± 34 | 43 ± 14 | -5.5 | -36 to 25 | 0.712 | 0.861 |
| **VLDL-2 Cholesterol, mg/dL** |  |  |  |  |  |  |
| Control | 5.6 ± 2.5 | 3.9 ± 1.5 |  |  |  |  |
| EXam | 3.4 ± 1.1 | 3.1 ± 1.7 | 0.5 | -0.9 to 1.8 | 0.487 | 0.962 |
| EXpm | 2.8 ± 1.0 | 1.5 ± 0.7 | -0.4 | 1.8 to 0.9 | 0.539 | 0.842 |
| **VLDL-2 Free cholesterol, mg/dL** |  |  |  |  |  |  |
| Control | 2.6 ± 1.3 | 1.8 ± 0.7 |  |  |  |  |
| EXam | 1.5 ± 0.5 | 1.3 ± 0.8 | 0.3 | -0.4 to 0.9 | 0.391 | 0.962 |
| EXpm | 1.3 ± 0.6 | 0.7 ± 0.3 | -0.1 | -0.8 to 0.5 | 0.648 | 0.852 |
| **VLDL-2 Phospholipids, mg/dL** |  |  |  |  |  |  |
| Control | 5.8 ± 2.8 | 4.5 ± 1.7 |  |  |  |  |
| EXam | 4.0 ± 1.4 | 3.7 ± 1.9 | 0.3 | -1.2 to 1.7 | 0.722 | 0.962 |
| EXpm | 3.4 ± 1.2 | 2.1 ± 0.7 | -0.8 | -2.2 to 0.7 | 0.287 | 0.648 |
| **VLDL-2 Triglycerides, mg/dL** |  |  |  |  |  |  |
| Control | 23.7 ± 12.8 | 18.1 ± 6.8 |  |  |  |  |
| EXam | 15.9 ± 6.1 | 15.4 ± 9.5 | 1.3 | -5.7 to 8.3 | 0.701 | 0.648 |
| EXpm | 13.9 ± 6.2 | 8.1 ± 2.9 | -3.6 | -10.6 to 3.2 | 0.289 | 0.648 |
| **VLDL-3 Cholesterol, mg/dL** |  |  |  |  |  |  |
| Control | 5.3 ± 2.6 | 4.2 ± 1.7 |  |  |  |  |
| EXam | 3.1 ± 1.6 | 2.8 ± 1.5 | 0.01 | -1.2 to 1.3 | 0.991 | 0.991 |
| EXpm | 2.4 ± 1.2 | 1.4 ± 0.9 | -0.7 | -1.9 to 0.5 | 0.261 | 0.636 |
| **VLDL-3 Free cholesterol, mg/dL** |  |  |  |  |  |  |
| Control | 2.7 ± 1.6 | 2.0 ± 0.8 |  |  |  |  |
| EXam | 1.4 ± 0.8 | 1.2 ± 0.5 | 0.2 | -0.5 to 0.8 | 0.647 | 0.962 |
| EXpm | 1.0 ± 0.5 | 0.8 ± 0.3 | 0.1 | -0.6 to 0.7 | 0.860 | 0.928 |
| **VLDL-3 Phospholipids, mg/dL** |  |  |  |  |  |  |
| Control | 6.0 ± 2.6 | 4.7 ± 1.6 |  |  |  |  |
| EXam | 4.0 ± 1.5 | 3.7 ± 1.7 | 0.2 | -1.2 to 1.5 | 0.800 | 0.962 |
| EXpm | 3.4 ± 1.3 | 2.2 ± 0.8 | -0.6 | -2.0 to 0.7 | 0.321 | 0.676 |
| **VLDL-3 Triglycerides, mg/dL** |  |  |  |  |  |  |
| Control | 17.3 ± 9.6 | 13.4 ± 5.8 |  |  |  |  |
| EXam | 10.9 ± 5.1 | 11.0 ± 7.8 | 0.8 | -4.6 to 6.4 | 0.754 | 0.962 |
| EXpm | 9.9 ± 5.7 | 5.5 ± 2.9 | -3.0 | -8.5 to 2.4 | 0.261 | 0.636 |
| **VLDL-4 Cholesterol, mg/dL** |  |  |  |  |  |  |
| Control | 1.5 ± 0.4 | 5.2 ± 1.5 |  |  |  |  |
| EXam | 4.5 ± 2.5 | 3.9 ± 1.4 | -0.3 | -1.5 to 0.9 | 0.632 | 0.962 |
| EXpm | 3.2 ± 1.4 | 2.7 ± 0.8 | -0.5 | -1.7 to 0.7 | 0.396 | 0.734 |
| **VLDL-4 Free cholesterol, mg/dL** |  |  |  |  |  |  |
| Control | 3.2 ± 1.1 | 2.6 ± 0.6 |  |  |  |  |
| EXam | 2.2 ± 0.9 | 2.0 ± 0.6 | -0.1 | -0.7 to 0.5 | 0.752 | 0.962 |
| EXpm | 1.8 ± 0.7 | 1.4 ± 0.5 | -0.4 | -1.0 to 0.3 | 0.224 | 0.609 |
| **VLDL-4 Phospholipids, mg/dL** |  |  |  |  |  |  |
| Control | 6.6 ± 1.9 | 5.4 ± 1.2 |  |  |  |  |
| EXam | 5.1 ± 1.8 | 4.9 ± 1.8 | 0.1 | -1.1 to 1.4 | 0.803 | 0.962 |
| EXpm | 4.5 ± 1.4 | 3.3 ± 0.7 | -0.8 | -2.0 to 0.4 | 0.196 | 0.609 |
| **VLDL-4 Triglycerides, mg/dL** |  |  |  |  |  |  |
| Control | 11.8 ± 4.8 | 9.6 ± 2.7 |  |  |  |  |
| EXam | 8.1 ± 3.8 | 7.7 ± 2.9 | 0.1 | -2.5 to 2.7 | 0.932 | 0.980 |
| EXpm | 7.0 ± 2.7 | 5.6 ± 1.6 | -0.7 | -3.3 to 1.9 | 0.573 | 0.843 |
| **VLDL-5 Cholesterol, mg/dL** |  |  |  |  |  |  |
| Control | 1.5 ± 0.4 | 1.6 ± 0.5 |  |  |  |  |
| EXam | 1.3 ± 0.6 | 1.2 ± 0.6 | -0.4 | -0.8 to 0.1 | 0.102 | 0.569 |
| EXpm | 1.0 ± 0.5 | 1.2 ± 0.3 | -0.2 | -0.6 to 0.2 | 0.291 | 0.648 |
| **VLDL-5 Free cholesterol, mg/dL** |  |  |  |  |  |  |
| Control | 1.7 ± 0.9 | 1.0 ± 0.3 |  |  |  |  |
| EXam | 0.9 ± 0.4 | 0.6 ± 0.5 | 0.1 | -0.4 to 0.5 | 0.820 | 0.962 |
| EXpm | 0.9 ± 0.4 | 0.6 ± 0.2 | 0.0 | -0.5 to 0.5 | 0.908 | 0.928 |
| **VLDL-5 Phospholipids, mg/dL** |  |  |  |  |  |  |
| Control | 2.2 ± 0.4 | 2.1 ± 0.4 |  |  |  |  |
| EXam | 1.8 ± 0.5 | 1.6 ± 0.6 | -0.3 | -0.6 to 0.1 | 0.157 | 0.642 |
| EXpm | 1.7 ± 0.3 | 1.5 ± 0.3 | -0.3 | -0.6 to 0.1 | 0.118 | 0.549 |
| **VLDL-5 Triglycerides, mg/dL** |  |  |  |  |  |  |
| Control | 3.8 ± 0.8 | 3.1 ± 0.7 |  |  |  |  |
| EXam | 3.0 ± 0.7 | 2.8 ± 0.6 | 0.1 | -0.4 to 0.6 | 0.707 | 0.962 |
| EXpm | 3.0 ± 0.4 | 2.8 ± 0.3 | 0.2 | -0.3 to 0.6 | 0.510 | 0.836 |
| **IDL Apo-B100, mg/dL** |  |  |  |  |  |  |
| Control | 9.1 ± 2.0 | 6.9 ± 0.7 |  |  |  |  |
| EXam | 6.8 ± 2.1 | 5.8 ± 1.6 | 0.3 | -1.1 to 1.7 | 0.648 | 0.962 |
| EXpm | 5.7 ± 1.6 | 4.3 ± 1.0 | -0.3 | -1.7 to 1.1 | 0.659 | 0.852 |
| **IDL Cholesterol, mg/dL** |  |  |  |  |  |  |
| Control | 20.3 ± 7.6 | 15.3 ± 2.7 |  |  |  |  |
| EXam | 13.8 ± 5.9 | 10.4 ± 4.6 | 0.1 | -4.4 to 4.5 | 0.969 | 0.986 |
| EXpm | 9.4 ± 3.8 | 8.0 ± 2.3 | 0.2 | -4.2 to 4.6 | 0.935 | 0.935 |
| **IDL Free Cholesterol, mg/dL** |  |  |  |  |  |  |
| Control | 6.3 ± 2.1 | 4.9 ± 0.8 |  |  |  |  |
| EXam | 4.3 ± 1.7 | 3.5 ± 1.4 | 0.0 | -1.1 to 1.2 | 0.941 | 0.980 |
| EXpm | 3.3 ± 1.3 | 2.4 ± 0.7 | -0.2 | -1.4 to 0.9 | 0.676 | 0.856 |
| **IDL Phospholipids, mg/dL** |  |  |  |  |  |  |
| Control | 14.3 ± 5.4 | 11.2 ± 2.6 |  |  |  |  |
| EXam | 10.1 ± 3.1 | 8.2 ± 3.7 | 0.0 | -2.7 to 2.7 | 0.976 | 0.986 |
| EXpm | 8.1 ± 2.9 | 5.2 ± 1.6 | -1.1 | -3.8 to 1.5 | 0.395 | 0.734 |
| **IDL Triglycerides, mg/dL** |  |  |  |  |  |  |
| Control | 36.0 ± 19.3 | 24.4 ± 8.1 |  |  |  |  |
| EXam | 21.4 ± 9.0 | 17.5 ± 11.5 | 1.7 | -8.5 to 12.0 | 0.731 | 0.962 |
| EXpm | 18.3 ± 9.3 | 9.6 ± 5.1 | -3.1 | -13.3 to 7.0 | 0.530 | 0.841 |
| **LDL Apo-B100, mg/dL** |  |  |  |  |  |  |
| Control | 74.7 ± 25.9 | 73.8 ± 21.4 |  |  |  |  |
| EXam | 68.9 ± 19.7 | 54.7 ± 18.9 | -8.9 | -19.0 to 1.1 | 0.074 | 0.438 |
| EXpm | 51.6 ± 13.6 | 54.5 ± 11.9 | 2.7 | -7.2 to 12.5 | 0.579 | 0.843 |
| **LDL Cholesterol, mg/dL** |  |  |  |  |  |  |
| Control | 114 ± 36 | 120 ± 29 |  |  |  |  |
| EXam | 113 ± 28 | 94 ± 24 | -20.3 | -36,1 to -4.6 | **0.011** | 0.210 |
| EXpm | 92 ± 19 | 88 ± 19 | -12.0 | -27.4 to 3.5 | 0.119 | 0.549 |
| **LDL Free Cholesterol, mg/dL** |  |  |  |  |  |  |
| Control | 32.5 ± 8.3 | 35.2 ± 8.2 |  |  |  |  |
| EXam | 33.2 ±7.1 | 29.8 ± 6.0 | -5.4 | -9.4 to -1.4 | **0.009** | 0.210 |
| EXpm | 28.9 ± 4.7 | 27.3 ± 4.2 | -4.6 | -8.5 to -0.6 | **0.022** | 0.197 |
| **LDL Phospholipids, mg/dL** |  |  |  |  |  |  |
| Control | 64.2 ± 16.1 | 67.4 ± 13.3 |  |  |  |  |
| EXam | 64.6 ± 13.6 | 55.8 ± 12.6 | -9.8 | -16.7 to -2.9 | **0.005** | 0.200 |
| EXpm | 54.7 ± 9.1 | 50.8 ± 8.9 | -7.2 | -13.9 to -0.5 | **0.033** | 0.274 |
| **LDL Triglycerides, mg/dL** |  |  |  |  |  |  |
| Control | 20.7 ± 5.3 | 18.0 ± 3.6 |  |  |  |  |
| EXam | 17.8 ± 6.7 | 15.9 ± 4.3 | 0.8 | -2.1 to 3.6 | 0.583 | 0.962 |
| EXpm | 14.5 ± 3.2 | 14.6 ± 2.5 | 2.1 | -0.7 to 4.9 | 0.128 | 0.549 |
| **LDL-1 Apo-B100, mg/dL** |  |  |  |  |  |  |
| Control | 14.7 ± 1.7 | 14.5 ± 1.8 |  |  |  |  |
| EXam | 14.8 ± 3.9 | 13.7 ± 3.9 | -0.9 | -2.9 to 1.0 | 0.339 | 0.962 |
| EXpm | 14.0 ± 2.8 | 10.9 ± 1.7 | -3.0 | -5.0 to -1.1 | **0.003** | 0.050 |
| **LDL-1 Cholesterol, mg/dL** |  |  |  |  |  |  |
| Control | 28.9 ± 3.0 | 28.9 ± 3.6 |  |  |  |  |
| EXam | 29.8 ± 8.6 | 27.6 ± 9.5 | -2.4 | -6.8 to 2.0 | 0.273 | 0.882 |
| EXpm | 28.7 ± 6.8 | 20.2 ± 4.3 | -8.3 | -12.6 to -3.9 | **<0.001** | **0.018** |
| **LDL-1 Free cholesterol, mg/dL** |  |  |  |  |  |  |
| Control | 8.4 ± 0.8 | 8.4 ± 0.9 |  |  |  |  |
| EXam | 8.9 ± 2.4 | 8.3 ± 3.0 | -0.7 | -2.1 to 0.7 | 0.324 | 0.962 |
| EXpm | 8.6 ± 1.9 | 6.2 ± 1.2 | -2.2 | -3.6 to -0.9 | **0.002** | **0.039** |
| **LDL-1 Phospholipids, mg/dL** |  |  |  |  |  |  |
| Control | 16.1 ± 1.6 | 15.9 ± 1.9 |  |  |  |  |
| EXam | 16.6 ± 4.2 | 15.7 ± 5.0 | -1.0 | -3.2 to 1.3 | 0.387 | 0.962 |
| EXpm | 16.3 ± 3.6 | 11.8 ± 2.3 | -4.2 | -6.4 to -2.0 | **<0.001** | **0.018** |
| **LDL-1 Triglycerides, mg/dL** |  |  |  |  |  |  |
| Control | 6.7 ± 2.6 | 5.9 ± 1.9 |  |  |  |  |
| EXam | 5.7 ± 2.3 | 5.0 ± 2.2 | 0.2 | -1.0 to 1.4 | 0.760 | 0.962 |
| EXpm | 4.6 ± 1.4 | 4.6 ± 1.0 | 0.5 | -0.7 to 1.7 | 0.368 | 0.722 |
| **LDL-2 Apo-B100, mg/dL** |  |  |  |  |  |  |
| Control | 10.3 ± 2.2 | 12.0 ± 2.6 |  |  |  |  |
| EXam | 11.5 ± 3.3 | 11.7 ± 2.7 | -0.9 | -3.1 to 1.3 | 0.403 | 0.962 |
| EXpm | 11.9 ± 2.2 | 11.2 ± 1.6 | -1.5 | -3.6 to 0.7 | 0.176 | 0.606 |
| **LDL-2 Cholesterol, mg/dL** |  |  |  |  |  |  |
| Control | 19.3 ± 3.7 | 22.4 ± 4.8 |  |  |  |  |
| EXam | 21.8 ± 7.1 | 22.1 ± 6.5 | -1.6 | -6.2 to 2.9 | 0.475 | 0.962 |
| EXpm | 22.9 ± 5.1 | 20.4 ± 3.3 | -3.6 | -8.1 to 0.9 | 0.112 | 0.549 |
| **LDL-2 Free cholesterol, mg/dL** |  |  |  |  |  |  |
| Control | 6.0 ± 0.9 | 6.7 ± 1.6 |  |  |  |  |
| EXam | 6.6 ± 1.9 | 6.7 ± 2.2 | -0.3 | -1.8 to 1.3 | 0.739 | 0.962 |
| EXpm | 7.2 ± 2.0 | 6.5 ± 0.9 | -0.7 | -2.2 to 0.8 | 0.360 | 0.721 |
| **LDL-2 Phospholipids, mg/dL** |  |  |  |  |  |  |
| Control | 10.5 ± 1.9 | 12.2 ± 2.3 |  |  |  |  |
| EXam | 12.0 ± 3.6 | 12.3 ± 3.6 | -0.7 | -2.1 to 1.6 | 0.528 | 0.962 |
| EXpm | 12.8 ± 2.5 | 11.2 ± 1.6 | -2.1 | -4.4 to 0.3 | 0.079 | 0.530 |
| **LDL-2 Triglycerides, mg/dL** |  |  |  |  |  |  |
| Control | 2.7 ± 0.5 | 2.6 ± 0.5 |  |  |  |  |
| EXam | 2.8 ± 1.0 | 2.8 ± 1.0 | 0.0 | -0.4 to 0.4 | 0.859 | 0.965 |
| EXpm | 2.8 ± 0.6 | 2.1 ± 0.4 | -0.5 | -0.9 to -0.1 | **0.011** | 0.110 |
| **LDL-3 Apo-B100, mg/dL** |  |  |  |  |  |  |
| Control | 6.8 ± 4.1 | 10.1 ± 3.7 |  |  |  |  |
| EXam | 8.7 ± 3.7 | 8.4 ± 3.0 | -2.7 | -5.0 to -0.5 | **0.019** | 0.210 |
| EXpm | 8.3 ± 2.1 | 8.1 ± 2.1 | -2.9 | -5.2 to -0.7 | **0.010** | 0.110 |
| **LDL-3 Cholesterol, mg/dL** |  |  |  |  |  |  |
| Control | 10.9 ± 7.4 | 17.3 ± 6.7 |  |  |  |  |
| EXam | 14.7 ± 7.3 | 14.1 ± 5.6 | -5.4 | -9.8 to -1.1 | **0.014** | 0.210 |
| EXpm | 14.1 ± 3.7 | 13.2 ± 4.4 | -6.2 | -10.5 to -1.9 | **0.005** | 0.071 |
| **LDL-3 Free cholesterol, mg/dL** |  |  |  |  |  |  |
| Control | 3.6 ± 1.5 | 4.9 ± 1.7 |  |  |  |  |
| EXam | 4.5 ± 1.8 | 4.4 ± 1.3 | -1.1 | -2.2 to -0.1 | **0.027** | 0.249 |
| EXpm | 4.7 ± 0.8 | 4.3 ± 1.2 | -1.3 | -2.3 to -0.3 | **0.010** | 0.110 |
| **LDL-3 Phospholipids, mg/dL** |  |  |  |  |  |  |
| Control | 6.3 ± 3.6 | 9.6 ± 3.2 |  |  |  |  |
| EXam | 8.4 ± 3.5 | 8.2 ± 2.7 | -2.6 | -4.7 to -0.5 | **0.015** | 0.210 |
| EXpm | 8.3 ± 1.7 | 7.7 ± 2.2 | -3.2 | -5.3 to -1.1 | **0.003** | 0.050 |
| **LDL-3 Triglycerides, mg/dL** |  |  |  |  |  |  |
| Control | 3.0 ± 0.3 | 2.7 ± 0.4 |  |  |  |  |
| EXam | 2.9 ± 0.7 | 2.6 ± 0.7 | 0.1 | -0.2 to 0.3 | 0.641 | 0.962 |
| EXpm | 2.6 ± 0.4 | 2.3 ± 0.3 | -0.1 | -0.3 to 0.2 | 0.524 | 0.841 |
| **LDL-4 Apo-B100, mg/dL** |  |  |  |  |  |  |
| Control | 6.1 ± 5.9 | 8.4 ± 5.8 |  |  |  |  |
| EXam | 5.9 ± 5.1 | 5.3 ± 4.8 | -2.7 | -6.5 to 1.2 | 0.168 | 0.647 |
| EXpm | 3.8 ± 4.4 | 5.1 ± 3.4 | -2.0 | -5.8 to 1.9 | 0.302 | 0.656 |
| **LDL-4 Cholesterol, mg/dL** |  |  |  |  |  |  |
| Control | 8.5 ± 9.7 | 13.0 ± 9.1 |  |  |  |  |
| EXam | 9.0 ± 7.5 | 8.0 ± 7.6 | -4.8 | -11.0 to 1.3 | 0.119 | 0.581 |
| EXpm | 5.9 ± 6.6 | 7.7 ± 5.6 | -3.7 | -9.8 to 2.4 | 0.225 | 0.609 |
| **LDL-4 Free cholesterol, mg/dL** |  |  |  |  |  |  |
| Control | 2.5 ± 2.4 | 3.6 ± 2.5 |  |  |  |  |
| EXam | 2.7 ± 1.8 | 2.3 ± 1.8 | -1.2 | -2.8 to 0.4 | 0.122 | 0.581 |
| EXpm | 1.7 ± 1.8 | 2.7 ± 1.6 | -0.4 | -1.9 to 1.2 | 0.623 | 0.843 |
| **LDL-4 Phospholipids, mg/dL** |  |  |  |  |  |  |
| Control | 4.7 ± 5.0 | 7.2 ± 4.6 |  |  |  |  |
| EXam | 5.1 ± 3.9 | 4.6 ± 4.1 | -2.6 | -5.8 to 0.7 | 0.112 | 0.581 |
| EXpm | 3.4 ± 3.8 | 4.5 ± 2.9 | -1.9 | -5.1 to 1.3 | 0.233 | 0.613 |
| **LDL-4 Triglycerides, mg/dL** |  |  |  |  |  |  |
| Control | 1.2 ± 1.1 | 1.5 ± 1.0 |  |  |  |  |
| EXam | 1.3 ± 1.4 | 1.3 ± 0.9 | -0.2 | -0.9 to 0.6 | 0.661 | 0.962 |
| EXpm | 0.8 ± 1.0 | 1.2 ± 0.7 | 0.1 | -0.6 to 0.8 | 0.859 | 0.928 |
| **LDL-5 Apo-B100, mg/dL** |  |  |  |  |  |  |
| Control | 11.9 ± 6.6 | 10.5 ± 6.6 |  |  |  |  |
| EXam | 9.0 ± 6.8 | 6.3 ± 4.6 | -1.1 | -4.7 to 2.5 | 0.540 | 0.962 |
| EXpm | 4.1 ± 4.0 | 7.1 ± 4.1 | 3.1 | -0.4 to 6.7 | 0.078 | 0.530 |
| **LDL-5 Cholesterol, mg/dL** |  |  |  |  |  |  |
| Control | 16.6 ± 9.6 | 15.0 ± 9.4 |  |  |  |  |
| EXam | 12.5 ± 9.5 | 8.6 ± 6.6 | -2.2 | -7.6 to 3.2 | 0.414 | 0.962 |
| EXpm | 5.6 ± 5.6 | 9.7 ± 6.1 | 3.7 | -1.6 to 9.1 | 0.157 | 0.604 |
| **LDL-5 Free cholesterol, mg/dL** |  |  |  |  |  |  |
| Control | 3.8 ± 2.4 | 3.7 ± 2.5 |  |  |  |  |
| EXam | 3.0 ± 2.5 | 2.2 ± 1.7 | -0.7 | -2.1 to 0.7 | 0.328 | 0.962 |
| EXpm | 1.4 ± 1.4 | 2.8 ± 1.8 | 0.9 | -0.4 to 2.3 | 0.170 | 0.606 |
| **LDL-5 Phospholipids, mg/dL** |  |  |  |  |  |  |
| Control | 8.9 ± 5.1 | 8.3 ± 4.8 |  |  |  |  |
| EXam | 7.3 ± 4.6 | 5.2 ± 3.4 | -1.1 | -3.7 to 1.5 | 0.381 | 0.962 |
| EXpm | 3.6 ± 3.1 | 5.8 ± 2.9 | 2.0 | -0.5 to 4.5 | 0.113 | 0.549 |
| **LDL-5 Triglycerides, mg/dL** |  |  |  |  |  |  |
| Control | 2.7 ± 1.3 | 2.1 ± 1.2 |  |  |  |  |
| EXam | 2.1 ± 1.4 | 1.5 ± 0.9 | 0.1 | -0.6 to 0.8 | 0.833 | 0.962 |
| EXpm | 1.1 ± 0.9 | 1.4 ± 0.6 | -0.7 | 0.04 to 1.4 | **0.036** | 0.276 |
| **LDL-6 Apo-B100, mg/dL** |  |  |  |  |  |  |
| Control | 29.7 ± 9.4 | 20.7 ± 9.1 |  |  |  |  |
| EXam | 21.2 ± 7.6 | 15.6 ± 3.9 | 0.6 | -6.0 to 7.3 | 0.843 | 0.962 |
| EXpm | 15.3 ± 5.7 | 14.0 ± 3.4 | 3.2 | -3.4 to 9.7 | 0.324 | 0.676 |
| **LDL-6 Cholesterol, mg/dL** |  |  |  |  |  |  |
| Control | 34.0 ± 10.2 | 24.1 ± 11.2 |  |  |  |  |
| EXam | 25.5 ± 8.3 | 18.8 ± 4.9 | 0.5 | -7.0 to 8.1 | 0.884 | 0.970 |
| EXpm | 18.0 ± 6.7 | 16.4 ± 4.8 | 3.4 | -4.1 to 10.9 | 0.355 | 0.721 |
| **LDL-6 Free cholesterol, mg/dL** |  |  |  |  |  |  |
| Control | 7.9 ± 2.7 | 6.3 ± 2.8 |  |  |  |  |
| EXam | 6.6 ± 2.5 | 5.4 ± 2.2 | -0.3 | -2.4 to 1.8 | 0.786 | 0.962 |
| EXpm | 5.3 ± 2.1 | 4.7 ± 0.9 | -0.3 | -2.4 to 1.8 | 0.767 | 0.882 |
| **LDL-6 Phospholipids, mg/dL** |  |  |  |  |  |  |
| Control | 18.9 ± 5.3 | 13.8 ± 5.3 |  |  |  |  |
| EXam | 14.5 ± 4.1 | 11.2 ± 2.7 | 0.3 | -3.6 to 4.2 | 0.893 | 0.970 |
| EXpm | 11.0 ± 3.6 | 10.0 ± 2.1 | 1.4 | -2.5 to 5.3 | 0.460 | 0.793 |
| **LDL-6 Triglycerides, mg/dL** |  |  |  |  |  |  |
| Control | 5.1 ± 2.5 | 3.6 ± 1.6 |  |  |  |  |
| EXam | 3.5 ± 1.7 | 2.8 ± 1.0 | 0.7 | -0.4 to 1.9 | 0.185 | 0.660 |
| EXpm | 2.2 ± 1.0 | 3.0 ± 1.1 | 1.9 | 0.8 to 3.0 | **0.001** | **0.039** |
| **HDL Apo-A1, mg/dL** |  |  |  |  |  |  |
| Control | 138 ± 8 | 136 ± 10 |  |  |  |  |
| EXam | 133 ± 10 | 123 ± 11 | -10.9 | -20.3 to -1.4 | **0.025** | 0.249 |
| EXpm | 133 ± 12 | 128 ± 12 | -5.8 | -15.2 to 3.6 | 0.217 | 0.609 |
| **HDL Apo-A2, mg/dL** |  |  |  |  |  |  |
| Control | 35.5 ± 3.2 | 32.6 ± 2.2 |  |  |  |  |
| EXam | 30.0 ± 4.4 | 27.0 ± 4.7 | -1.9 | -5.3 to 1.5 | 0.263 | 0.876 |
| EXpm | 29.3 ± 3.6 | 29.3 ± 3.3 | 0.5 | -2.9 to 3.9 | 0.760 | 0.882 |
| **HDL Cholesterol, mg/dL** |  |  |  |  |  |  |
| Control | 49.5 ± 4.8 | 49.6 ± 4.0 |  |  |  |  |
| EXam | 49.2 ± 7.0 | 44.7 ± 4.2 | -4.3 | -9.0 to 0.4 | 0.070 | 0.435 |
| EXpm | 48.3 ± 6.4 | 49.5 ± 5.6 | 0.8 | -3.9 to 5.5 | 0.732 | 0.861 |
| **HDL Free Cholesterol, mg/dL** |  |  |  |  |  |  |
| Control | 12.8 ± 3.0 | 12.4 ± 2.3 |  |  |  |  |
| EXam | 13.0 ± 2.9 | 13.1 ± 5.6 | -0.2 | -2.9 to 2.5 | 0.882 | 0.970 |
| EXpm | 14.8 ± 4.0 | 11.8 ± 1.7 | -1.9 | -4.6 to 0.7 | 0.137 | 0.549 |
| **HDL Phospholipids, mg/dL** |  |  |  |  |  |  |
| Control | 75.7 ± 8.3 | 73.9 ± 11.1 |  |  |  |  |
| EXam | 73.7 ± 7.4 | 68.8 ± 10.8 | -4.5 | -10.7 to 1.8 | 0.151 | 0.642 |
| EXpm | 77.4 ± 7.6 | 70.4 ± 7.9 | -4.7 | -10.8 to 1.4 | 0.122 | 0.549 |
| **HDL Triglycerides, mg/dL** |  |  |  |  |  |  |
| Control | 20.7 ± 5.3 | 9.4 ± 3.0 |  |  |  |  |
| EXam | 8.4 ± 2.3 | 8.2 ± 1.8 | 0.2 | -1.8 to 2.1 | 0.845 | 0.962 |
| EXpm | 8.7 ± 2.5 | 7.0 ± 1.7 | -1.0 | -2.9 to 0.9 | 0.282 | 0.648 |
| **HDL-1 Apo-A1, mg/dL** |  |  |  |  |  |  |
| Control | 20.6 ± 7.8 | 18.6 ± 9.0 |  |  |  |  |
| EXam | 22.0 ± 8.6 | 21.8 ±10.9 | 0.9 | -4.0 to 5.8 | 0.693 | 0.962 |
| EXpm | 25.2 ± 6.4 | 22.5 ± 3.9 | 0.9 | -3.9 to 5.7 | 0.711 | 0.861 |
| **HDL-1 Apo-A2, mg/dL** |  |  |  |  |  |  |
| Control | 2.5 ± 1.0 | 1.9 ± 1.2 |  |  |  |  |
| EXam | 1.9 ± 0.7 | 1.7 ± 0.7 | 0.1 | -0.6 to 0.8 | 0.753 | 0.962 |
| EXpm | 2.2 ± 0.5 | 2.0 ± 0.5 | 0.2 | -0.4 to 0.9 | 0.489 | 0.828 |
| **HDL-1 Cholesterol, mg/dL** |  |  |  |  |  |  |
| Control | 13.3 ± 4.2 | 12.0 ± 2.6 |  |  |  |  |
| EXam | 14.2 ± 4.6 | 12.8 ± 4.2 | 0.3 | -2.6 to 3.1 | 0.846 | 0.962 |
| EXpm | 14.5 ± 3.7 | 13.5 ± 1.9 | 1.2 | -1.7 to 4.0 | 0.407 | 0.739 |
| **HDL-1 Free cholesterol, mg/dL** |  |  |  |  |  |  |
| Control | 3.7 ± 0.9 | 3.5 ± 1.0 |  |  |  |  |
| EXam | 3.8 ± 1.2 | 3.8 ± 1.6 | 0.0 | -0.8 to 0.9 | 0.930 | 0.980 |
| EXpm | 4.4 ± 1.2 | 3.7 ± 0.5 | -0.2 | -1.1 to 0.7 | 0.632 | 0.843 |
| **HDL-1 Phospholipids, mg/dL** |  |  |  |  |  |  |
| Control | 16.7 ± 5.2 | 15.4 ± 5.7 |  |  |  |  |
| EXam | 17.7 ± 5.9 | 16.9 ± 6.1 | 0.2 | -3.0 to 3.4 | 0.903 | 0.971 |
| EXpm | 19.9 ± 3.8 | 17.3 ± 2.5 | -0.2 | -3.3 to 3.0 | 0.909 | 0.928 |
| **HDL-1 Triglycerides, mg/dL** |  |  |  |  |  |  |
| Control | 2.8 ± 1.3 | 2.2 ± 1.4 |  |  |  |  |
| EXam | 2.0 ± 0.5 | 1.9 ± 0.8 | 0.2 | -0.6 to 1.0 | 0.576 | 0.962 |
| EXpm | 2.2 ± 0.9 | 1.8 ± 0.7 | 0.1 | -0.7 to 0.8 | 0.893 | 0.928 |
| **HDL-2 Apo-A1, mg/dL** |  |  |  |  |  |  |
| Control | 19.9 ± 3.5 | 17.1 ± 4.5 |  |  |  |  |
| EXam | 17.6 ± 2.0 | 15.9 ± 2.9 | 0.2 | -1.9 to 2.4 | 0.828 | 0.962 |
| EXpm | 19.0 ± 2.4 | 16.4 ± 2.2 | -0.1 | -2.2 to 2.0 | 0.934 | 0.935 |
| **HDL-2 Apo-A2, mg/dL** |  |  |  |  |  |  |
| Control | 5.0 ± 1.5 | 3.8 ± 1.4 |  |  |  |  |
| EXam | 3.8 ± 0.9 | 3.3 ± 0.9 | 0.1 | -0.8 to 1.0 | 0.840 | 0.962 |
| EXpm | 4.3 ± 0.9 | 3.2 ± 0.8 | -0.2 | -1.0 to 0.7 | 0.665 | 0.852 |
| **HDL-2 Cholesterol, mg/dL** |  |  |  |  |  |  |
| Control | 8.6 ± 2.7 | 7.6 ± 2.8 |  |  |  |  |
| EXam | 7.9 ± 1.5 | 7.4 ± 1.9 | 0.02 | -1.3 to 1.3 | 0.972 | 0.986 |
| EXpm | 9.1 ± 1.8 | 7.9 ± 0.9 | -0.2 | -1.4 to 1.1 | 0.798 | 0.907 |
| **HDL-2 Free cholesterol, mg/dL** |  |  |  |  |  |  |
| Control | 1.9 ± 0.7 | 1.8 ± 0.7 |  |  |  |  |
| EXam | 1.8 ± 0.4 | 1.7 ± 0.3 | -0.1 | -0.4 to 0.2 | 0.556 | 0.962 |
| EXpm | 2.1 ± 0.3 | 2.0 ± 0.3 | -0.1 | -0.4 to 0.2 | 0.594 | 0.843 |
| **HDL-2 Phospholipids, mg/dL** |  |  |  |  |  |  |
| Control | 14.7 ± 5.2 | 12.7 ± 5.3 |  |  |  |  |
| EXam | 13.2 ± 2.7 | 12.4 ± 3.4 | 0.2 | -2.0 to 2.5 | 0.820 | 0.962 |
| EXpm | 15.4 ± 2.7 | 12.5 ± 1.8 | -0.8 | -3.0 to 1.3 | 0.610 | 0.843 |
| **HDL-2 Triglycerides, mg/dL** |  |  |  |  |  |  |
| Control | 2.0 ± 0.8 | 1.7 ± 0.9 |  |  |  |  |
| EXam | 1.5 ± 0.5 | 1.5 ± 0.4 | 0.1 | -0.3 to 0.5 | 0.620 | 0.962 |
| EXpm | 1.7 ± 0.5 | 1.4 ± 0.4 | -0.1 | -0.5 to 0.3 | 0.610 | 0.843 |
| **HDL-3 Apo-A1, mg/dL** |  |  |  |  |  |  |
| Control | 30.9 ± 5.4 | 27.3 ± 5.6 |  |  |  |  |
| EXam | 26.8 ± 3.4 | 24.2 ± 5.2 | -0.8 | -3.9 to 2.3 | 0.602 | 0.962 |
| EXpm | 29.5 ± 4.5 | 24.6 ± 4.1 | -2.1 | -5.2 to 1.0 | 0.164 | 0.606 |
| **HDL-3 Apo-A2, mg/dL** |  |  |  |  |  |  |
| Control | 7.9 ± 1.6 | 6.7 ± 1.6 |  |  |  |  |
| EXam | 6.3 ± 1.1 | 5.4 ± 1.3 | -0.2 | -1.2 to 0.9 | 0.764 | 0.962 |
| EXpm | 6.6 ± 1.2 | 5.9 ± 1.1 | -0.1 | -1.1 to 0.9 | 0.887 | 0.928 |
| **HDL-3 Cholesterol, mg/dL** |  |  |  |  |  |  |
| Control | 10.7 ± 1.9 | 9.9 ± 1.8 |  |  |  |  |
| EXam | 9.7 ± 1.2 | 8.8 ± 1.4 | -0.5 | -1.6 to 0.5 | 0.296 | 0.926 |
| EXpm | 10.7 ± 1.7 | 9.4 ± 1.5 | -0.6 | -1.7 to 0.4 | 0.214 | 0.609 |
| **HDL-3 Free cholesterol, mg/dL** |  |  |  |  |  |  |
| Control | 1.9 ± 0.4 | 1.9 ± 0.4 |  |  |  |  |
| EXam | 1.6 ± 0.4 | 1.5 ± 0.2 | -0.3 | -0.6 to 0.004 | 0.051 | 0.364 |
| EXpm | 1.8 ± 0.3 | 1.6 ± 0.5 | -0.2 | -0.5 to 0.1 | 0.220 | 0.609 |
| **HDL-3 Phospholipids, mg/dL** |  |  |  |  |  |  |
| Control | 17.8 ± 2.7 | 16.5 ± 3.5 |  |  |  |  |
| EXam | 16.0 ± 2.6 | 14.2 ± 2.9 | -1.1 | -2.8 to 0.7 | 0.212 | 0.733 |
| EXpm | 17.2 ± 2.1 | 15.3 ± 2.4 | -1.0 | -2.7 to 0.7 | 0.240 | 0.615 |
| **HDL-3 Triglycerides, mg/dL** |  |  |  |  |  |  |
| Control | 2.6 ± 0.7 | 2.1 ± 0.7 |  |  |  |  |
| EXam | 1.8 ± 0.7 | 1.7 ± 0.4 | 0.1 | -0.4 to 0.5 | 0.734 | 0.962 |
| EXpm | 1.9 ± 0.5 | 1.7 ± 0.4 | 0.0 | -0.5 to 0.4 | 0.909 | 0.928 |
| **HDL-4 Apo-A1, mg/dL** |  |  |  |  |  |  |
| Control | 66.6 ± 16.6 | 72.3 ± 13.2 |  |  |  |  |
| EXam | 66.1 ± 12.9 | 60.3 ± 11.0 | -9.8 | -18.0 to -1.6 | **0.019** | 0.210 |
| EXpm | 58.4 ± 8.3 | 62.7 ± 8.8 | -3.1 | -11.1 to 5.0 | 0.440 | 0.772 |
| **HDL-4 Apo-A2, mg/dL** |  |  |  |  |  |  |
| Control | 19.0 ± 4.6 | 19.5 ± 3.8 |  |  |  |  |
| EXam | 17.0 ± 4.9 | 15.1 ± 5.1 | -2.1 | -4.8 to 0.7 | 0.128 | 0.581 |
| EXpm | 14.6 ± 3.4 | 16.5 ± 2.6 | 0.5 | -2.2 to 3.2 | 0.727 | 0.861 |
| **HDL-4 Cholesterol, mg/dL** |  |  |  |  |  |  |
| Control | 16.2± 5.9 | 18.8 ± 4.6 |  |  |  |  |
| EXam | 16.6 ± 4.6 | 15.0 ± 4.7 | -3.4 | -6.6 to -0.2 | **0.037** | 0.281 |
| EXpm | 14.0 ± 3.7 | 16.8 ± 3.2 | -0.3 | -3.5 to 2.8 | 0.833 | 0.925 |
| **HDL-4 Free cholesterol, mg/dL** |  |  |  |  |  |  |
| Control | 2.2 ± 1.0 | 3.0 ± 1.2 |  |  |  |  |
| EXam | 2.3 ± 1.0 | 2.1 ± 0.8 | -0.9 | -1.5 to -0.4 | **0.002** | 0.200 |
| EXpm | 2.0 ± 0.8 | 2.6 ± 1.0 | -0.4 | -1.0 to 0.2 | 0.197 | 0.609 |
| **HDL-4 Phospholipids, mg/dL** |  |  |  |  |  |  |
| Control | 25.8 ± 4.8 | 28.2 ± 4.0 |  |  |  |  |
| EXam | 25.7 ± 4.3 | 23.6 ± 4.7 | -4.0 | -6.9 to -1.2 | **0.006** | 0.200 |
| EXpm | 23.6 ± 3.5 | 24.3 ± 3.4 | -2.3 | -5.1 to 0.5 | 0.103 | 0.549 |
| **HDL-4 Triglycerides, mg/dL** |  |  |  |  |  |  |
| Control | 3.9 ± 0.7 | 3.5 ± 0.4 |  |  |  |  |
| EXam | 3.1 ± 1.2 | 3.0 ± 0.7 | 0.1 | -0.4 to 0.6 | 0.784 | 0.962 |
| EXpm | 2.9 ± 0.7 | 2.4 ± 0.6 | -0.3 | -0.8 to 0.2 | 0.223 | 0.609 |

**Supplementary Table 6.**  Lipid main- and subfractions and their contents of lipids and lipoproteins after five days of high-fat diet and at study completion, according to group, in men with overweight/obesity. Estimates with *p* values for the effect of evening exercise (EXpm) compared with morning exercise (EXam), from linear mixed models, with *q* values from Benjamini-Hochberg correction. Fasted samples were obtained in the morning, postprandial samples in the evening.

|  | **Fasted** | | | **Postprandial** | | |
| --- | --- | --- | --- | --- | --- | --- |
|  | **Estimate** | ***p* value** | ***q* value** | **Estimate** | ***p* value** | ***q* value** |
| Total Apo-A1, mg/dL | 0.9 | 0.826 | 0.988 | 2.3 | 0.641 | 0.844 |
| Total Apo-A2, mg/dL | 0.0 | 0.968 | 0.988 | 1.4 | 0.271 | 0.551 |
| Total Apo-B100, mg/dL | 0.2 | 0.960 | 0.988 | 8.9 | 0.115 | 0.383 |
| Total Cholesterol, mg/dL | -4.0 | 0.657 | 0.988 | 1.9 | 0.857 | 0.927 |
| Total Triglycerides, mg/dL | 5.1 | 0.779 | 0.988 | -33.6 | 0.135 | 0.384 |
| VLDL Apo-B100, mg/dL | -0.2 | 0.849 | 0.988 | -1.0 | 0.276 | 0.551 |
| VLDL Cholesterol, mg/dL | -1.4 | 0.647 | 0.988 | -5.3 | 0.074 | 0.337 |
| VLDL Free Cholesterol, mg/dL | -0.2 | 0.868 | 0.988 | -1.8 | 0.170 | 0.459 |
| VLDL Phospholipids, mg/dL | -0.3 | 0.922 | 0.988 | -5.4 | 0.087 | 0.353 |
| VLDL Triglycerides, mg/dL | 5.4 | 0.684 | 0.988 | -27.4 | 0.126 | 0.383 |
| VLDL-1 Cholesterol, mg/dL | 0.0 | 0.976 | 0.988 | -2.0 | 0.201 | 0.502 |
| VLDL-1 Free cholesterol, mg/dL | 0.1 | 0.826 | 0.988 | -1.4 | 0.092 | 0.353 |
| VLDL-1 Phospholipids, mg/dL | 0.8 | 0.549 | 0.988 | -2.5 | 0.116 | 0.383 |
| VLDL-1 Triglycerides, mg/dL | 5.5 | 0.519 | 0.988 | -14.4 | 0.199 | 0.502 |
| VLDL-2 Cholesterol, mg/dL | -0.1 | 0.806 | 0.988 | -1.5 | **0.016** | 0.337 |
| VLDL-2 Free cholesterol, mg/dL | -0.1 | 0.671 | 0.988 | -0.6 | **0.032** | 0.337 |
| VLDL-2 Phospholipids, mg/dL | 0.2 | 0.768 | 0.988 | -1.4 | **0.031** | 0.337 |
| VLDL-2 Triglycerides, mg/dL | 1.1 | 0.607 | 0.988 | -6.4 | **0.041** | 0.337 |
| VLDL-3 Cholesterol, mg/dL | -0.3 | 0.556 | 0.988 | -1.0 | 0.073 | 0.337 |
| VLDL-3 Free cholesterol, mg/dL | -0.1 | 0.716 | 0.988 | -0.3 | 0.265 | 0.551 |
| VLDL-3 Phospholipids, mg/dL | -0.1 | 0.762 | 0.988 | -1.2 | 0.050 | 0.337 |
| VLDL-3 Triglycerides, mg/dL | 0.2 | 0.901 | 0.988 | -4.9 | 0.062 | 0.337 |
| VLDL-4 Cholesterol, mg/dL | -0.6 | 0.350 | 0.988 | -0.5 | 0.455 | 0.700 |
| VLDL-4 Free cholesterol, mg/dL | -0.4 | 0.173 | 0.988 | -0.4 | 0.181 | 0.477 |
| VLDL-4 Phospholipids, mg/dL | -0.4 | 0.392 | 0.988 | -1.1 | 0.074 | 0.337 |
| VLDL-4 Triglycerides, mg/dL | -0.3 | 0.697 | 0.988 | -1.4 | 0.266 | 0.551 |
| VLDL-5 Cholesterol, mg/dL | -0.2 | 0.220 | 0.988 | 0.1 | 0.577 | 0.790 |
| VLDL-5 Free cholesterol, mg/dL | -0.1 | 0.532 | 0.988 | 0.0 | 0.824 | 0.918 |
| VLDL-5 Phospholipids, mg/dL | -0.2 | 0.194 | 0.988 | 0.0 | 0.823 | 0.918 |
| VLDL-5 Triglycerides, mg/dL | -0.1 | 0.466 | 0.988 | 0.0 | 0.805 | 0.918 |
| IDL Apo-B100, mg/dL | -0.2 | 0.625 | 0.988 | -0.7 | 0.228 | 0.518 |
| IDL Cholesterol, mg/dL | -0.8 | 0.553 | 0.988 | -0.1 | 0.924 | 0.953 |
| IDL Free Cholesterol, mg/dL | -0.2 | 0.653 | 0.988 | -0.4 | 0.404 | 0.700 |
| IDL Phospholipids, mg/dL | 0.2 | 0.850 | 0.988 | -1.4 | 0.208 | 0.502 |
| IDL Triglycerides, mg/dL | 0.5 | 0.880 | 0.988 | -5.5 | 0.136 | 0.384 |
| LDL Apo-B100, mg/dL | -0.9 | 0.816 | 0.988 | 10.6 | 0.050 | 0.337 |
| LDL Cholesterol, mg/dL | -7.5 | 0.371 | 0.988 | 6.3 | 0.441 | 0.700 |
| LDL Free Cholesterol, mg/dL | -1.8 | 0.429 | 0.988 | 0.2 | 0.910 | 0.948 |
| LDL Phospholipids, mg/dL | -2.9 | 0.419 | 0.988 | 1.9 | 0.586 | 0.790 |
| LDL Triglycerides, mg/dL | 1.3 | 0.175 | 0.988 | 1.2 | 0.326 | 0.627 |
| LDL-1 Apo-B100, mg/dL | -0.7 | 0.294 | 0.988 | -2.1 | 0.057 | 0.337 |
| LDL-1 Cholesterol, mg/dL | -1.8 | 0.197 | 0.988 | -5.8 | **0.023** | 0.337 |
| LDL-1 Free cholesterol, mg/dL | -0.5 | 0.282 | 0.988 | -1.5 | **0.048** | 0.337 |
| LDL-1 Phospholipids, mg/dL | -0.9 | 0.186 | 0.988 | -3.2 | **0.014** | 0.337 |
| LDL-1 Triglycerides, mg/dL | 0.3 | 0.394 | 0.988 | 0.3 | 0.429 | 0.700 |
| LDL-2 Apo-B100, mg/dL | -0.2 | 0.861 | 0.988 | -0.5 | 0.592 | 0.790 |
| LDL-2 Cholesterol, mg/dL | -0.3 | 0.911 | 0.988 | -1.9 | 0.390 | 0.700 |
| LDL-2 Free cholesterol, mg/dL | 0.2 | 0.795 | 0.988 | -0.4 | 0.576 | 0.790 |
| LDL-2 Phospholipids, mg/dL | -0.2 | 0.894 | 0.988 | -1.3 | 0.259 | 0.551 |
| LDL-2 Triglycerides, mg/dL | -0.2 | 0.104 | 0.988 | -0.6 | **0.017** | 0.337 |
| LDL-3 Apo-B100, mg/dL | -1.2 | 0.301 | 0.988 | -0.2 | 0.872 | 0.927 |
| LDL-3 Cholesterol, mg/dL | -2.2 | 0.346 | 0.988 | -0.7 | 0.738 | 0.918 |
| LDL-3 Free cholesterol, mg/dL | -0.3 | 0.606 | 0.988 | -0.2 | 0.717 | 0.908 |
| LDL-3 Phospholipids, mg/dL | -1.2 | 0.296 | 0.988 | -0.5 | 0.591 | 0.790 |
| LDL-3 Triglycerides, mg/dL | 0.0 | 0.839 | 0.988 | -0.1 | 0.291 | 0.571 |
| LDL-4 Apo-B100, mg/dL | -2.3 | **0.033** | 0.552 | 0.5 | 0.794 | 0.918 |
| LDL-4 Cholesterol, mg/dL | -3.9 | **0.045** | 0.559 | 0.8 | 0.788 | 0.918 |
| LDL-4 Free cholesterol, mg/dL | -0.8 | 0.156 | 0.988 | 0.7 | 0.367 | 0.680 |
| LDL-4 Phospholipids, mg/dL | -1.9 | **0.041** | 0.559 | 0.5 | 0.750 | 0.918 |
| LDL-4 Triglycerides, mg/dL | -0.2 | 0.252 | 0.988 | 0.2 | 0.589 | 0.790 |
| LDL-5 Apo-B100, mg/dL | -0.1 | 0.917 | 0.988 | 3.6 | 0.067 | 0.337 |
| LDL-5 Cholesterol, mg/dL | -0.7 | 0.607 | 0.988 | 4.9 | 0.089 | 0.353 |
| LDL-5 Free cholesterol, mg/dL | -0.1 | 0.728 | 0.988 | 1.4 | 0.071 | 0.337 |
| LDL-5 Phospholipids, mg/dL | -0.3 | 0.672 | 0.988 | 2.7 | 0.052 | 0.337 |
| LDL-5 Triglycerides, mg/dL | 0.1 | 0.575 | 0.988 | 0.6 | 0.124 | 0.383 |
| LDL-6 Apo-B100, mg/dL | 4.1 | **0.022** | 0.437 | -0.8 | 0.768 | 0.918 |
| LDL-6 Cholesterol, mg/dL | 4.7 | **0.019** | 0.437 | -1.2 | 0.702 | 0.900 |
| LDL-6 Free cholesterol, mg/dL | 1.2 | **0.015** | 0.437 | -0.7 | 0.436 | 0.700 |
| LDL-6 Phospholipids, mg/dL | 2.4 | **0.018** | 0.437 | -0.9 | 0.589 | 0.790 |
| LDL-6 Triglycerides, mg/dL | 0.9 | **0.008** | 0.437 | 1.1 | **0.012** | 0.337 |
| HDL Apo-A1, mg/dL | 1.4 | 0.749 | 0.988 | 4.8 | 0.345 | 0.652 |
| HDL Apo-A2, mg/dL | 0.0 | 0.994 | 0.994 | 2.2 | 0.115 | 0.383 |
| Total HDL Cholesterol, mg/dL | 1.2 | 0.612 | 0.988 | 5.1 | **0.038** | 0.337 |
| HDL Free Cholesterol, mg/dL | 0.5 | 0.323 | 0.988 | -1.7 | 0.216 | 0.503 |
| HDL Phospholipids, mg/dL | 1.5 | 0.579 | 0.988 | -0.1 | 0.983 | 0.983 |
| HDL Triglycerides, mg/dL | -0.2 | 0.804 | 0.988 | -1.3 | 0.138 | 0.384 |
| HDL-1 Apo-A1, mg/dL | 1.4 | 0.302 | 0.988 | -0.1 | 0.970 | 0.980 |
| HDL-1 Apo-A2, mg/dL | 0.1 | 0.542 | 0.988 | 0.1 | 0.699 | 0.900 |
| HDL-1 Cholesterol, mg/dL | 1.1 | 0.252 | 0.988 | 1.0 | 0.422 | 0.700 |
| HDL-1 Free cholesterol, mg/dL | 0.3 | 0.290 | 0.988 | -0.2 | 0.588 | 0.790 |
| HDL-1 Phospholipids, mg/dL | 1.0 | 0.320 | 0.988 | -0.3 | 0.818 | 0.918 |
| HDL-1 Triglycerides, mg/dL | 0.1 | 0.760 | 0.988 | -0.2 | 0.571 | 0.790 |
| HDL-2 Apo-A1, mg/dL | 0.4 | 0.502 | 0.988 | -0.2 | 0.826 | 0.918 |
| HDL-2 Apo-A2, mg/dL | 0.0 | 0.963 | 0.988 | -0.3 | 0.445 | 0.700 |
| HDL-2 Cholesterol, mg/dL | 0.1 | 0.813 | 0.988 | -0.1 | 0.866 | 0.927 |
| HDL-2 Free cholesterol, mg/dL | 0.0 | 0.946 | 0.988 | 0.0 | 0.800 | 0.918 |
| HDL-2 Phospholipids, mg/dL | 0.2 | 0.770 | 0.988 | -0.9 | 0.416 | 0.700 |
| HDL-2 Triglycerides, mg/dL | 0.0 | 0.784 | 0.988 | -0.2 | 0.253 | 0.551 |
| HDL-3 Apo-A1, mg/dL | 0.2 | 0.875 | 0.988 | -1.2 | 0.450 | 0.700 |
| HDL-3 Apo-A2, mg/dL | -0.1 | 0.713 | 0.988 | 0.1 | 0.888 | 0.934 |
| HDL-3 Cholesterol, mg/dL | 0.0 | 0.978 | 0.988 | 0.0 | 0.941 | 0.960 |
| HDL-3 Free cholesterol, mg/dL | 0.1 | 0.690 | 0.988 | 0.1 | 0.399 | 0.700 |
| HDL-3 Phospholipids, mg/dL | 0.1 | 0.851 | 0.988 | 0.2 | 0.835 | 0.918 |
| HDL-3 Triglycerides, mg/dL | 0.0 | 0.929 | 0.988 | -0.1 | 0.562 | 0.790 |
| HDL-4 Apo-A1, mg/dL | -0.2 | 0.952 | 0.988 | 6.2 | 0.120 | 0.383 |
| HDL-4 Apo-A2, mg/dL | 0.0 | 0.962 | 0.988 | 2.5 | 0.077 | 0.337 |
| HDL-4 Cholesterol. mg/dL | 0.0 | 0.958 | 0.988 | 3.0 | 0.061 | 0.337 |
| HDL-4 Free cholesterol. mg/dL | 0.1 | 0.629 | 0.988 | 0.6 | 0.077 | 0.337 |
| HDL-4 Phospholipids. mg/dL | 0.1 | 0.960 | 0.988 | 1.7 | 0.211 | 0.502 |
| HDL-4 Triglycerides. mg/dL | 0.0 | 0.937 | 0.988 | -0.4 | 0.116 | 0.383 |

**Supplementary Table 7.** Composition of experimental diets and breakdown of meals provided to participants for 11 days. Data are means ± standard deviation.

|  | **Total day** | **Breakfast** | **Lunch** | **Dinner** |
| --- | --- | --- | --- | --- |
| Energy (kJ) | 12408 ± 956 | 4151±375 | 4017 ± 316 | 4236 ± 376 |
| Carbohydrate (g) | 118 ± 9 | 40 ± 4 | 39 ± 3 | 40 ± 4 |
| Sugars (g) | 61 ± 5 | 17 ± 2 | 28 ± 3 | 16 ± 1 |
| Carbohydrate (% Total energy intake) | 15 ± 0 | 15 ± 0 | 15 ± 0 | 15 ± 0 |
| Protein (g) | 134 ± 12 | 43 ± 4 | 41 ± 3 | 44 ± 4 |
| Protein (% Total energy intake) | 20 ± 0 | 20 ± 0 | 20 ± 0 | 20 ± 0 |
| Fat (g) | 223 ± 18 | 75 ± 7 | 72 ± 6 | 77 ± 7 |
| Fat (% Total energy intake) | 65 ± 0 | 65 ± 0 | 65 ± 0 | 65 ± 0 |
| Saturated fat (g) | 116 ± 9 | 40 ± 3 | 47 ± 4 | 29 ± 2 |
| Polyunsaturated fat (g) | 12 ± 1 | 6 ± 0 | 3 ± 1 | 3 ± 1 |
| Monounsaturated fat (g) | 49 ± 4 | 19 ± 3 | 10 ± 1 | 20 ± 3 |
| Fibre (g) | 24 ± 2 | 9 ± 1 | 3 ± 1 | 12 ± 1 |

**Supplementary Table 8.** Example of meals provided during the high-fat diet.

| **Breakfast** | **Lunch** | **Dinner** |
| --- | --- | --- |
| 1   - Almond butter pancakes - Peanut butter - Greek yoghurt | 1   - Cauliflower and cheese bake - Coconut almond flour bread - White bread | 1   - Beef sausages - Mixed vegetables - Chocolate brownie |
| 2   - Scrambled eggs - Bacon - White bread - Butter | 2   - Coconut lemon balls - Cheese and chive muffins - Greek yoghurt (coconut) | 2   - Chorizo and cauliflower casserole - Mixed vegetables - White rice - Chocolate mousse |

**Supplementary Figures**

**Supplementary Fig. 1.** Principal component analysis of lipid subfraction variables before and after 5 days of high-fat diet in morning (fasted) (**A-B**) and evening (postprandial) (**C-D**) samples. Numbers on the plots are participant numbers.

**Supplementary Fig. 2.** Cholesterol and Apolipoprotein-B100 (Apo-B) in LDL measured in the postprandial state in participants (*n* = 24) before (habitual diet) and after five days of high-fat diet (**A**) Total serum cholesterol, (**B**) Cholesterol in LDL subfraction 1-6, (**C**) Total serum Apo-B, (**D**) Change in Apo-B in LDL subfraction 1-6, as percentage of total Apo-B in LDL, after five days of high-fat diet. Bars show means, error bars are SD, and symbols show individual values. **p* < 0.05

**Supplementary Fig. 3.** Scores and loading plots from ASCA for discriminating lipoprotein profiles at Visit 3 (after exercise/no exercise) from Visit 2 in the postprandial state. (**A**) Scores for changes between Visit 2 and Visit 3 in the control group, (**B**) Loadings for changes between Visit 2 and Visit 3 in the control group, (**C**) Scores for changes after morning exercise (EXam) and evening exercise (EXpm) between Visit 2 and Visit 3, compared to changes in the control group, (**D**), Loadings for changes after morning exercise (EXam) and evening exercise (EXpm) between Visit 2 and Visit 3, compared with changes in the control group. CH = cholesterol, FC = free cholesterol, PL = phospholipids, TG = triglycerides, AB = Apolipoprotein-B100, A1 = Apolipoprotein A-1, A2 = Apolipoprotein A-2.
